# Sequential Targeting of CD52 and TNF Allows Early Minimization Therapy in Kidney Transplantation: From a Biomarker to Targeting in a Proof-Of-Concept Trial

**DOI:** 10.1371/journal.pone.0169624

**Published:** 2017-01-13

**Authors:** Ondrej Viklicky, Petra Hruba, Stefan Tomiuk, Sabrina Schmitz, Bernhard Gerstmayer, Birgit Sawitzki, Patrick Miqueu, Petra Mrazova, Irena Tycova, Eva Svobodova, Eva Honsova, Uwe Janssen, Hans-Dieter Volk, Petra Reinke

**Affiliations:** 1 Department of Nephrology, Institute for Clinical and Experimental Medicine, Prague, Czech Republic; 2 Transplant Laboratory, Institute for Clinical and Experimental Medicine, Prague, Czech Republic; 3 Miltenyi Biotec GmbH, Bergisch Gladbach, Germany; 4 Institute of Medical Immunology, Charité University Medicine Berlin, Germany; 5 Berlin-Brandenburg Center for Regenerative Medicine (BCRT), Charité University Medicine Berlin, Germany; 6 Institut National de la Santé et de la Recherche Médicale INSERM U1064, France; 7 Institut de Transplantation Urologie Néphrologie du Centre Hospitalier Universitaire Hôtel Dieu, Nantes, France; 8 Department of Immunogenetics, Institute for Clinical and Experimental Medicine, Prague, Czech Republic; 9 Department of Clinical and Transplant Pathology, Institute for Clinical and Experimental Medicine, Prague, Czech Republic; 10 Department of Nephrology and Intensive Care Medicine, Charité University Medicine Berlin, Germany; Universidad de Navarra, SPAIN

## Abstract

**Background:**

There is high medical need for safe long-term immunosuppression monotherapy in kidney transplantation. Selective targeting of post-transplant alloantigen-(re)activated effector-T cells by anti-TNF antibodies after global T cell depletion may allow safe drug minimization, however, it is unsolved what might be the best maintenance monotherapy.

**Methods:**

In this open, prospective observational single-centre trial, 20 primary deceased donor kidney transplant recipients received 2x20 mg Alemtuzumab (d0/d1) followed by 5 mg/kg Infliximab (d2). For 14 days all patients received only tacrolimus, then they were allocated to either receive tacrolimus (TAC, n = 13) or sirolimus (SIR, n = 7) monotherapy, respectively. Protocol biopsies and extensive immune monitoring were performed and patients were followed-up for 60 months.

**Results:**

TAC-monotherapy resulted in excellent graft survival (5yr 92%, 95%CI: 56.6–98.9) and function, normal histology, and no proteinuria. Immune monitoring revealed low intragraft inflammation (urinary IP-10) and hints for the development of operational tolerance signature in the TAC- but not SIR-group. Remarkably, the TAC-monotherapy was successful in all five presensitized (ELISPOT+) patients. However, recruitment into SIR-arm was stopped (after n = 7) because of high incidence of proteinuria and acute/chronic rejection in biopsies. No opportunistic infections occurred during follow-up.

**Conclusions:**

In conclusion, our novel fast-track TAC-monotherapy protocol is likely to be safe and preliminary results indicated an excellent 5-year outcome, however, a full–scale study will be needed to confirm our findings.

**Trial Registration:**

EudraCT Number: 2006-003110-18

## Introduction

Minimization of immunosuppression is a major task for improving long-term outcome and decreasing direct and indirect costs after kidney transplantation [1]. Minimization however increases the risk of rejection, particularly in high-responder patients [2, 3]. Recent research focusses on biomarkers for identifying patients who need less immunosuppression in order to allow biomarker-driven safe minimization (www.biodrim.eu) [4, 5]. Several groups demonstrated that the occurrence of high levels of donor-reactive memory/effector T cells as detected by Elispot-analysis is associated with poorer outcome [6–8]. Very recent data suggest, stratification of patients based on the pretransplant Elispot seems to allow safe CNI-free immunosuppression in some kidney transplant patients [9]. However, as this approach is limited to the subset of “low-responder” patients only, novel therapeutic strategies are needed to convert the majority of patients into “low responders” allowing minimization of immunosuppression. A robust protocol reaching this goal is not available [10].

Minimization of immunosuppression seems to be supported by profound peri-transplant immune cell depletion as result of reduced clonal size of alloreactive T/B cells. However, controversial outcome on minimized immunosuppression after depletional induction has been reported [11, 12]. Beside profound depletion/control of T cells, particularly early post-transplant, long-term control of alloresponse is dependent on active regulatory mechanisms [13–15], which may be further enhanced by mTOR inhibitors such as sirolimus [16].

Induction therapy with depleting biologics (polyclonal rabbit antithymocyte globulin or alemtuzumab) has been shown to be associated with expansion of regulatory cells [17, 18]. However, depleting agent alone was not enough for successful minimization to tacrolimus monotherapy, even in preselected patients [19]. Possible explanation for the conflicting results is the relative resistance of memory/effector T/B cells to depleting antibodies in presensitized patients and their preferential (alloantigen-driven) expansion in the lymphopenic recipient [20]. So donor-specific Teff cells represent not only a biomarker for patients´ stratification but also a promising therapeutical target.

TNF plays a key role in activating innate and adaptive immune response. In its soluble form, TNF-trimers can trigger multiple inflammatory reactions on multiple receptor-bearing target cells [21]. It was previously shown that memory and / or effector T cells express membrane bound TNF and are susceptible to anti-TNF antibody mediated complement-dependent lysis [22]. In addition, TNF monomers, dimers, and trimers are transiently detectable as transmembrane molecules on recently activated T and innate immune cells [23]. In contrast to the TNF-receptor fusion protein, ethernacept, that binds only the soluble TNF, anti-TNF antibodies, like infliximab, bind also strongly to transmembrane TNF (tmTNF+) on (re)activated immune cells and induce apoptosis of targeted tmTNF + cells both *in vitro* and *in vivo* [22, 24, 25], own unpublished observations). As the tmTNF expression is very transient following (re)activation, targeting of tmTNF+ cells is relatively selective for very recently activated effector cells.

Therefore, we hypothesized that few days after transplantation alloantigen-(re)activated memory/effector cells can be specifically targeted. To test the hypothesis that anti-TNF mAb if given at right time post-transplantation might allow safe monotherapy in almost all patients, we performed a Proof-of-Concept (PoC) trial, supported by the European Programs (RISET and BIO-DrIM networks). Primary deceased donor kidney transplant recipients received sequential induction therapy with alemtuzumab and infliximab followed either by tacrolimus or sirolimus monotherapy. The data from 5 year follow-up support our concept and suggest safety and efficacy of new induction approach with early tacrolimus monotherapy that was associated with regulatory B-cell gene signature and control of intrarenal inflammation.

## Methods

### Study design and patients

**Fig 1:**
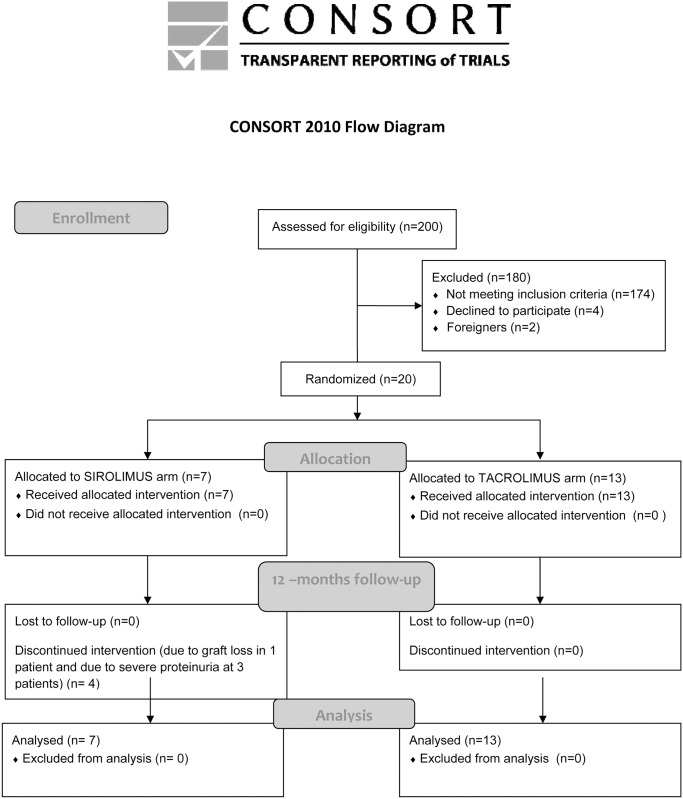
Consort 2010 flow diagram.

The study was originally planned as prospective 12 months open label single centre PoC study, and approved by the IRB of the Institute for Clinical and Experimental Medicine, Prague, the State Institute for Drug (1012/06) and Healthcare products Regulatory Agency (European Union Drug Regulating Authorities Clinical Trials [EudraCT] Number 2006-003110-18) under the umbrella of the 6^th^ Frame Program of the European Union “Reprogramming the Immune System for the Establishment of Tolerance (RISET)” project (clinicaltrials.gov register entry: NCT02711202). Follow-up analyses were supported by the 7^th^ Frame Program of the EU “Biomarker-driven Immunosuppression (Bio-DrIM)” project. The Ethics Committee of the Institute for Clinical and Experimental Medicine approved the study protocol and all patients signed informed consent to participate in the study (No. 1012/06). The primary endpoint of the study was patient and graft survival at 12 months. The secondary endpoints were as follows: graft function estimated by serum creatinine, frequency of biopsy proven rejection at 1 year, intrarenal and peripheral blood expression of rejection- or tolerance-associated genes. None of the transplant donors were from vulnerable population and all donors or next of kin provided consent that was freely given. We enrolled 20 primary low risk kidney transplant recipients (Table 1), the inclusion and exclusion criteria are described in detail below. The first patient was enrolled on January 2007 and the last one on March 2009. According to recommendation for studies dealing with minimization strategies [26], the patient enrollment was slow to allow careful patient monitoring, this means not all available patients were included in the study. Protocol safety and efficacy was evaluated repeatedly by DSMB. Participants were enrolled consecutively by physician and assigned according to patient order (odd and even) when entering the study by study coordinator. After the treatment allocation of the first 14 patients, the last 6 patients were assigned only into the TAC-group because of limited efficacy observed in the SIR-group (Fig 1). All but one tacrolimus monotherapy treated patients have continued until M60, while at M36, all sirolimus monotherapy assigned patients were off scheduled therapy (S1 Table). Prospective immune monitoring was performed before transplantation and at POD21, M2, M3, M6 and M12. Protocol kidney graft biopsies were performed at POD21 (a week after conversion to sirolimus in the conversion arm), and at 3M and 12M. Clinical data were followed post-study 12M period until M60 to better describe safety and efficacy of this protocol. To compare the outcome of study groups with standard of care triple immunosuppression treated patients, 174 patients fulfilling the same inclusion criteria were involved.

**Table 1:** Clinical and demographic characteristics of kidney transplant recipients in TAC- and SIR-group.

	Tacrolimus	Sirolimus	P value
**N**	13	7	
Patient age, y*	55 [20; 63]	46 [26; 55]	0.03
Donor age, y*	53 [26; 63]	53 [41; 62]	0.55
**HLA mismatch** *	3 [1; 4]	3 [2; 3]	0.81
Peak PRA*	2 [0; 8]	2 [0; 8]	0.27
**Cold ischemia**, hours, range	18.9 (13.5–25)	19.2 (16.1–23.5)	0.84
**Cause of renal failure**			
IgA nephropathy	2	2	
Glomerulonephritis	3	1	
Diabetic or ischemic nephropathy	0	1	
TIN	2	1	
Nephrosclerosis	4	2	
Others	2	0	

* median [min; max]

### Inclusion and exclusion criteria

The inclusion criteria were as follows: first deceased-donor kidney transplantation, age >18 years, donor <65 years, CMV/EBV seropositivity, panel reactive antibody (PRA) <10% and written consent with the participation in the study.

The exclusion criteria were as follows: previous transplantation, combined transplantation, immunosuppression less than 6 months prior transplantation, induction therapy with antibodies, leukopenia < 4000, thrombocytopenia < 100 000, haemoglobin < 80 g/l, previous therapy with ATG, anti-CD3 monoclonal antibody or anti-TNF-α monoclonals, tuberculosis history, positivity of anti-HCV positivity, HBsAg, HIV, history of malignancy, allergy to study medication, fertile women without contraception, pregnancy, and breastfeeding mothers.

### Immunosuppression

Alemtuzumab (Genzyme Europe B.V. Naarden, The Netherlands) 20 mg diluted in saline was given in two slow i.v. infusions doses before reperfusion of the allograft and on the first day as described by others [27]. Thirty minutes before each alemtuzumab application, patients received methylprednisolone 500mg i.v.

Infliximab (Janssen Biologics B.V. Leiden, The Netherlands) was given as a single dose at 5mg/kg diluted in 500 mL of saline at d2 and before application patients had received hydrocortisone 100mg as a local praxis. lnfliximab dose was chosen as recommended for the Crohn’s disease therapy.

Before the surgery, patients were randomized to receive either long-term tacrolimus or sirolimus monotherapy from day 15 postoperation while the first 14 days all patients received tacrolimus monotherapy. The reason for delayed switch to sirolimus was the negative impact of rapamycin on wound healing and the recent observation of higher antibody-mediated rejection rates in patients treated with sirolimus monotherapy immediately after Alemtuzumab induction [28]. Tacrolimus (Prograf^®^, Astellas) was given in standard doses 0,1mg/kg bid, the first dose was given before surgery. Two days post operation, the whole blood trough levels were evaluated and target levels were 10–15 ng/mL for the first month, 5–15 ng/ml for the first 3 months and 5-10ng/ml later on.

Sirolimus (Rapamune^®^, Wyeth), 4 mg morning dose, was given from day 15 on. In this arm, the last tacrolimus dose was given 24 hours before. Long-term sirolimus trough levels were 5–10 ng/mL. In patients with early acute rejection within the first 14 days the study protocol allowed later sirolimus switch.

In a case of case biopsy-confirmed clinically relevant acute or chronic rejections, patients got additionally mycophenolate mofetil and/or steroids maintenance therapy as appropriate.

### Monitoring viral infections and anti-infective prophylaxis

BK Polyomavirus (BKV) loads were determined in the urine and blood while cytomegalovirus (CMV) and Epstein-Barr virus (EBV) were determined in the blood using PCR at POD14, M1, 2 and 3.

All patients received CMV prophylaxis with intravenous ganciclovir for the first week followed for 90 days oral valganciclovir prophylaxis. According to local IRB recommendation each patient received nindrazid 300 mg daily prophylaxis for 6 months along with vitamin B6 substitution. CMV/EBV seronegative recipients were excluded from this study.

### Biopsies

Protocol kidney graft biopsies were performed routinely on d21, M3 and M12 and case biopsies were indicated in clinical suspicion of rejection or significant proteinuria. Renal biopsies were obtained under ultrasound guidance (Toshiba, Power Vision 6000) using a 14-gauge Tru-Cut needle (Uni-Cut Nadeln, Angiomed, Germany). Most of the renal tissue was processed for conventional histology. Histological examination was interpreted according to the 2005 Banff classification criteria. The residual portion (2mm) of the cortical or juxtamedullary zone of the renal tissue were immediately placed in RNA later (Ambion Corporation, Austin, TX), snap frozen and stored at -80°C until RNA extraction.

### Immune monitoring

#### Flow cytometry

Peripheral blood mononuclear cells (PBMC) were isolated by density gradient centrifugation and flow cytometric analysis was performed according to standard protocols using the following antibodies (clone): anti-CD4-PE (13B8.2), anti-CD8-ECD (SFCl21Thy2D3), anti-CD19-ECD (J3-119) or anti-CD3-PC5 (UCHT1). Following staining, samples were analyzed using an FC 500 flow cytometer (Beckman Coulter, Brea, CA, USA) and CxP and Kaluza software (Beckman Coulter, Brea, CA, USA).

#### Quantification of urinary IP-10 protein expression

Urinary samples were collected (2x2ml) and frozen at -80°C until measurement in bulk analyses using an Enzyme-linked immunosorbent assay (IP-10 Elisa Hycult HK311). Based on recent data, normal levels are <100 pg/ml and levels of >200 pg/ml indicate intragraft inflammation, mostly related to alloreactivity [29].

#### ELISpot

ELISpot was performed as described elsewhere [4]. Briefly, liquid nitrogen-frozen peripheral blood mononuclear cells (PBMC (3x10^5^ cells/well) were stimulated for 24 hours with donor cells (1:1), CMV-pp65/IE-1 peptide pool, SEB (positive control), or culture media (negative control). Spots were counted using the AID-Reader. Relevant donor response was defined at > 20 spots/300,000 PBMC and high-risk patients at > 200 Spots/300, 000 PBMC [9]. Because of profound depletion, only pretransplant samples were analyzed.

#### Antibody testing

The specificity of HLA and MICA antibodies was defined by LABScreen Mixed and Single Antigen (SAB) class I and class II beads (OneLambda Inc.) in serum of patients at M36 posttransplant according to the manufacturer’s protocol. Samples were analyzed by the Luminex 200 flowanalyzer (One Lambda Inc.) using the HLA Fusion software (version no.2). Beads with normalized mean fluorescence intensity (MFI) values >1000MFI (class I) and 2000MFI (class II) were considered to be DSA positive.

### Whole blood microarray

#### RNA isolation

RNA was isolated from peripheral blood, collected before Tx, and at W3, M2, M3, M6 and M12 posttransplant, using the PAXgene Blood RNA kit (Qiagen, Hilden, Germany) according to manufacturer´s instructions. Peripheral vein blood was drawn directly into PAXgene Blood RNA tubes (Qiagen, Hilden, Germany) and stored at -20°C until analysis. Quality and integrity of PAXgene RNA were determined using the Agilent RNA 6000 Nano Kit on the Agilent 2100 Bioanalyzer (Agilent Technologies). Samples with RNA integrity number below five were excluded from microarray hybridization.

#### RNA amplification and labeling

The protocol of RNA amplification and labeling was slightly modified to enable processing of samples in a 96 well format. Briefly, 150 ng of each total RNA in 1.5 μl bidest H20 was pipetted in the respective well of a 96 well plate (4titude). To each well, 2μl Spike-In and 1.8 μl T7 Primer (both Agilent Technologies) were added, the plate was sealed and samples were incubated for 10 min. at 65°C, followed by a subsequent cooling step to 4°C. 2 μl 5x First Strand Buffer, 1 μl 0.1 M DTT, 0.5 μl 10 mM dNTP and 1.2 μl Affinity Script RNAse Block mix were added and incubated for another 120 min at 40°C, followed by 15 min at 65°C and then cooled down the plate to 4°C. The amplification and labeling step was performed by adding 0.75 μl bidest H20, 3.2 μl 5x transcription buffer, 0.6 μl 0.1 M DTT, 1 μl NTP mix, 0.21 μl T7 RNA polymerase and 0.24 μl Cyanine 3-CTP and incubating the samples for 120 min at 40°C, followed by cooling the plate to 4°C. For purification of labeled cRNAs, to each well 84 μl bidest H_2_0 was added and the entire 100 μl were transferred to a 96 deep well plate (Greiner Bio One, Ref. 780271). To this plate 350 μl RLT buffer and 250 μl ETOH were added, mixed and 700 μl per labeled cRNA was transferred to the 96 sample plate (Qiagen, Hilden, Germany) and further processed according to [30]. Yields of cRNA and the dye incorporation rate were measured with the ND-1000 Spectrophotometer (Thermo Scientific).

#### Hybridization of RISET 2.0 Agilent custom microarrays

Hybridization *of RISET 2*.*0 Agilent custom microarrays* was performed as described elsewhere in detail [31]. Briefly, 0.6 μg Cy3-labeled fragmented cRNA in hybridization buffer was hybridized overnight (17 hours, 65°C) to RISET 2.0 microarrays [32] and subsequently washed.

#### Scanning and data analysis

Scanning and data analysis were performed using Agilent’s Microarray Scanner System (Agilent Technologies Inc.). The Agilent Feature Extraction Software (FES version 10.5.1.1) was used to read out and process the microarray image files. FES-derived output data files were further processed using the Rosetta Resolver gene expression data analysis system (version 7.1.0.2., Rosetta Inpharmatics LLC). The data have been deposited in NCBI’s Gene Expression Omnibus [33] and are accessible through GEO Series accession number GSE39299 (http://www.ncbi.nlm.nih.gov/geo/query/acc.cgi?acc=GSE39299). Heatmaps of top-ranked differentially expressed genes were constructed using MultiExperiment Viewer 4.6.0 (TM4, Boston, MA) [34].

#### Microarray statistical analysis

After inter-array quantile normalization, two-tailed t-tests (unequal variance) were conducted in order to identify genes with statistically significant expression differences (p≤0.05) between two sample groups. The statistical test was complemented by a non-statistical quantification of the median expression difference.

Lists of genes found to be discriminatory between different sample groups were analyzed for a statistically significant enrichment of biological pathway annotation terms in comparison to the complete RISET 2.0 microarray configuration. Term enrichment relative to the expected background distribution was scored using Fisher’s exact test. Annotations were derived from different sources, e.g., Gene Ontology (GO, www.geneontology.org), signaling pathway membership, sequence motifs, chromosomal proximity, literature keywords, and cell-specific marker genes.

### RT-qPCR analysis

The most differently expressed genes revealed by microarray analysis of blood samples using the RISET microarray platform and genes associated with transplantation tolerance published elsewhere [31] were next validated by RT-qPCR analysis. For RT-qPCR analysis the following probes were used (CD79B-Hs00236881_m1, CD200-Hs01033303_m1, CD247-Hs00167901_m1, C4A; LOC100293534; C4B-Hs00246758_m1, FN1-Hs01549976_m1, FCRL1-Hs00364705_m1, FCRL2-Hs00229156_m1, HS3ST1-Hs01099196_m1, IL8-Hs99999034_m1, IRF5-Hs00158114_m1, MS4A1-Hs00174849_m1, PNOC-Hs00918595_m1, SH2D1B-Hs01592483_m1, SLC8A1-Hs01062258_m1, TCL1A-Hs00951350_m1, TLR5-Hs00152825_m1, CCR2-Hs00356601_m1, CCL2-Hs00234140_m1, CCR5-Hs00152917_m1, CCR7-Hs99999080_m1). RNA was isolated from the whole blood as described above for microarray analysis or from protocol biopsies (W3, M3 and M12) using the RNeasy Fibrous Tissue Mini Kit (Qiagen, Hilden, Germany). RNA obtained from the whole blood (1–2 μg) or protocol biopsies (2μg) was reverse transcribed using Superscript Reverse transcriptase II (Invitrogen) or QuantiTect Reverse Transcription Kit (Qiagen, Hilden, Germany), respectively. The synthetized cDNA was subjected to RT-PCR analysis. Quantitative RT-PCR was performed using inventoried or custom-made Taqman assays in the whole blood or custom-made TaqMan low density array (Applied Biosystems) to analyze 19 genes in the biopsies RNA. Real-time RT-qPCR data were quantified using the SDS 2.4 software package (Applied Biosystems) and relative gene expression values were determined using the comparative 2^-ΔΔCt^ method of the Relative quantification (RQ) Manager Software v 1.2.1 (Applied Biosystems) with normalization to endogenous control (GAPDH-Hs99999905_m1 and PGK1-Hs99999906_m1) in the case of gene expression from biopsies and to HPRT1-Hs01003267_m1 in the case of the whole blood gene expression. Probe PNOC was due to high number of missing values (37%) excluded from the analysis. All investigated mRNAs were measured in triplicates for each sample.

### Statistics

The comparisons of tacrolimus (n = 13) and sirolimus arm (n = 7) of the study were performed according to the intention to treat principle. Normality of the data was tested using Kolmogorov-Smirnov normality test. Two-tailed Mann-Whitney U test was used to compare medians between patient groups. Data are presented as means ± SEM, or medians (interquartile ranges). Two-sided P values were considered as statistically significant when it was less than 0.05. The rejection (both acute and chronic rejections) and proteinuria (defined as proteinuria > 1g/day) free intervals were determined using Kaplan–Meier estimates and groups were compared using the log-rank test.

Statistical significance of gene expression differences, revealed by RT-qPCR, in particular time points and overall difference within the study period was evaluated by GLMM (Generalized linear mixed model) model of SPSS that enables the analysis of variance when the same measurement is made several times on each subject and the target can have a non-normal distribution. The missing values were calculated as medians of particular gene expression for the same time points (the missing values represented maximally 10% of all measured values). Due to non-normal distribution of positively skewed data, we used gamma regression of the dependent variable. Statistical analyses were performed using SPSS v.20.0 (SPSS, Inc., Chicago, IL) and GraphPad InStat v. 3. 05 for Windows (GraphPad software, San Diego, CA).

## Results

### Patient and graft survival

**Fig 2:**
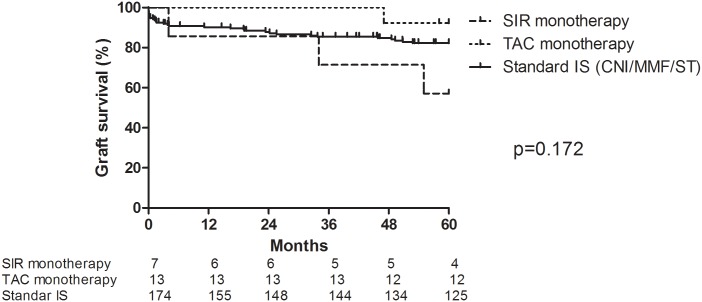
Graft survival in study groups and standard of care group. Patients received induction treatment with Alemtuzumab/Infliximab and long-term tacrolimus (TAC, n = 13) or sirolimus (SIR, n = 7) monotherapy. Standard of care group were patients with same inclusion criteria and from the same time period treated with CNI-based (tacrolimus or cyclosporine, mycophenolate mofetil and steroids) immunosuppression (control, n = 174). Log-rank test for comparison of all three groups (p = 0.172).

One patient each from SIR-group and TAC-group died at M35 and M48 due to peritonitis/sepsis and myocardial infarction with functional graft, respectively. One patient in the sirolimus arm (SIR-group) lost the graft at M4. There were two additional graft losses at M46 due to chronic AMR (TAC-group) and at M56 due to IgA nephropathy recurrence (SIR-group) (Fig 2). Comparing both study groups with standard of care group (low risk primary graft transplant cohort treated with CNI-based triple immunosuppression regimen), TAC-group exhibited similar 60M graft survival (Fig 2) comparing to other groups.

### Safety data

One patient from each arm had transiently BKV DNAemia at M2 and M3, respectively, without any sequelae. There was no detectable CMV DNAemia while 1 patient from SIR-group developed short-term weakly positive EBV-PCR at M3. One patient in TAC-group developed cancer duplicity (breast cancer at M24, stomach adenocarcinoma at M56). Detailed list of complications is given in Table 2. SIR-group received higher cumulative steroid dose per patient as compared to TAC-group (3931.1 vs. 2164.5 mg/patient/year).

**Table 2:** Severe adverse effects within 12 and 60M of follow-up in SIR- and TAC groups.

*Event*, *% (n)*	*12 months follow-up*		*12–60 months follow-up*	
	*Tacrolimus (n = 13)*	*Sirolimus (n = 7)*	*Tacrolimus (n = 13)*	*Sirolimus (n = 6)*
**Infections**				
Urinary tract infection	46.2 (6)	42.9 (3)	23.1(3)	16.7 (1)
Pneumonia		14.3 (1)	7.7 (1)	
Viral gastroenteritis	7.7 (1)			
Viral infection	7.7 (1)			
Pyelonephritis			7.7 (1)	
Peritonitis/septic shock				16.7 (1)
BKV	7.7(1)	14.3 (1)		
**Cardio vascular disorders**				
Ischemic cardiac disease	15.4 (2)	28.6 (2)	7.7 (1)	
Stroke	7.7 (1)		7.7 (1)	
**Renal and urinary disorders**				
Delayed graft function	15.4 (2)	14.3 (1)		
Hydrocele		14.3 (1)	7.7 (1)	
**Metabolism and nutrition disorders**				
Diabetes mellitus	15.4 (2)	57.1 (4)*	7.7 (1)	
Hyperparathyreosis	30.8 (4)	28.6 (2)	7.7 (1)	33.3 (2)
**Blood and lymphocyte disorders**				
Leucopenia	69.2 (9)	85.7 (6)		
**Cancer**				
Breast			7.7 (1)	
Stomach			7.7 (1)	

* one patient with preexisting diabetes

### Renal function and proteinuria

**Fig 3:**
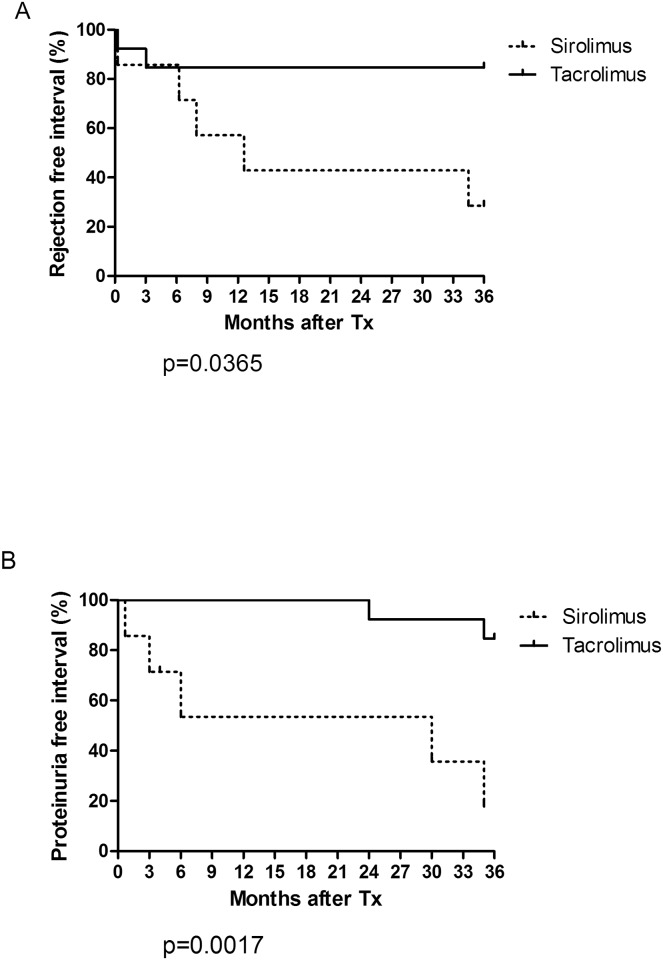
Rejection-free interval (A), defined as the interval between the time of transplantation and the first biopsy proven allograft rejection event (acute T-cell mediated rejection or acute/chronic humoral rejection) and proteinuria-free interval (B), as the interval between the time of transplantation and the first observed proteinuria >1g/24 hours shown in days by treatment group. P values were determined by log-rank analysis.

Mild proteinuria occurred in two patients from TAC-group at the 3^rd^ year. Contrary, 5/6 sirolimus patients (3 of them within 6M) developed severe proteinuria (Fig 3). After conversion to tacrolimus/MMF therapy the proteinuria significantly decreased in 4/5 patients. The TAC-group showed excellent serum creatinine levels (115.2 +/-51.7 and 127.3 +/-54.5μmol/L at M12 and 60, respectively). There were no significant differences in kidney graft function among groups (The comparison of mean serum creatinine levels of TAC- and SIR- groups with patients with standard of care immunosuppression (CNI/MMF/ST) is given in Supplementary S1 Fig). Trough levels of tacrolimus and sirolimus in individual patients are given in S2 Table.

### Rejections and histology

Taken into account any type of biopsy-proven rejection (humoral, cellular, mixed), both with (case biopsy) or without clinical signs (protocol biopsy), we observed within M60 follow-up 8 rejection episodes; in 6/7 sirolimus and 2/13 tacrolimus patients (Fig 3). Detailed histologic findings of all patients are given in S3 Table.

### Immune monitoring

#### Peripheral leucocyte counts

**Fig 4:**
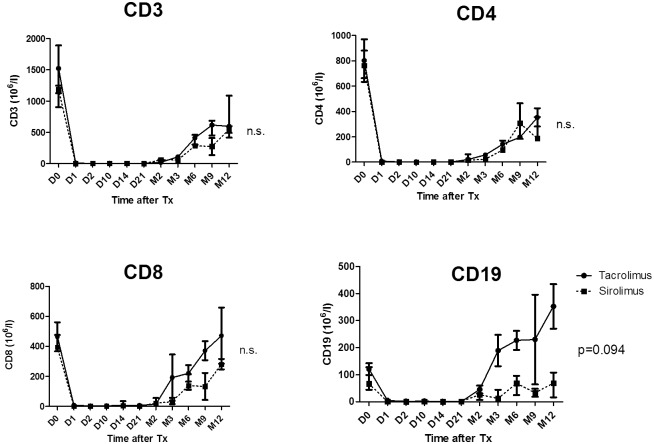
The effect of an initial aggressive immunosuppression (Alemtuzumab, anti-CD 52 therapeutical antibody targeting mature lymphocytes; Infliximab, anti-TNF-therapeutical antibody targeting T-lymphocytes and Methylprednisolone, shortly before and after Tx, followed by two weeks of Tacrolimus treatment) on T and B cells depletion and their subsequent recovery within 12 months in TAC- and SIR-group. CD3^+^ for T cells, CD4+ for TH lymphocytes, CD8+ for cytotoxic lymphocytes and CD19^+^ for B cells. Data are presented as means and SEM.

Lymphodepletion after Alemtuzumab induction was deep and lasted until the end of the first year as monitored in blood using flow cytometry. Interestingly, there was a tendency towards faster B cell repopulation in TAC-group as compared with sirolimus one (p = 0.09) (Fig 4).

#### Donor-reactive memory/effector T cells

**Fig 5:**
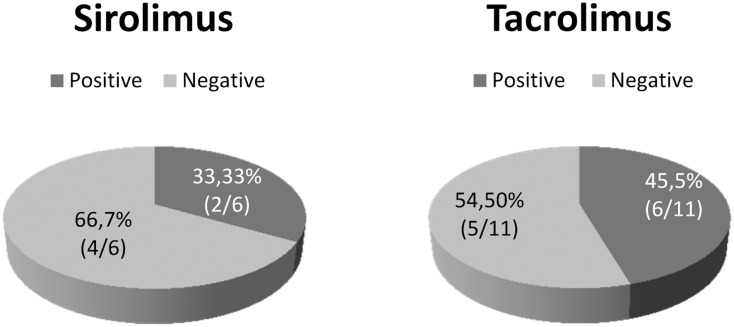
Distribution of patients in SIR and TAC groups depending on the pretransplant donor specific T-cell alloimmune response assessed by IFN-γ Elispot. Negative group: <20 spots/ 300 000 PBMC; positive group: >20 spots/ 300 000 PBMC.

Pre-transplant donor-reactive memory/effector T cell levels were detected above the cut-off for low/high responder [9] of 20 spots/300,000 PBMC in 5/11 and 2/6 patients analyzed by Elispot in the TAC- and SIR-group, respectively; four of them even at high-risk levels (≥200 spots/300,000) (S3 Table, Fig 5). All five T-cell presensitized patients of the TAC-group could be kept on monotherapy for the M60 follow-up period with excellent graft function. However, two of them showed histological signs of chronic AMR in M12 and M36 biopsy, respectively. In contrast, both high-responder patients of the SIR-group had to be switched to standard therapy within first year because of proteinuria and signs of chronic TCMR and AMR at M12 and M36 biopsy, respectively.

#### Urinary IP-10 revealed intragraft immune silence in tacrolimus monotherapy group

The urinary IP-10 (CXCL10) levels were monitored until M3 as indicator of tubular endothelial cell injury [35] and intragraft inflammation [29]. Remarkably, after normalization of IP-10 levels within the first week post-Tx, it kept at low level (<200 pg/ml) at M3 in 10/12 of all tacrolimus monotherapy patients, one each developed borderline or significantly enhanced levels (S3 Table). By contrast, 4/7 sirolimus patients developed strongly enhanced levels (p<0.05, Fisher-Yates test). The patient with peak levels of >10,000 pg/ml at M1 in SIR-group showed chronic TCMR within M12. Interestingly, its pre-Tx Elispot level was also highest by >1,000 spots/300,000 PBMC. The other three patients with enhanced IP-10 levels in SIR-group developed severe proteinuria.

#### Donor-specific antibodies

At M36, donor specific antibodies (DSA) were analyzed in 17 patients. Long-term post-transplant presence of DSA was found in 3 out of 17 serum samples (range 1,000–2,000 MFI). Two of them (one each group) developed late chronic AMR and the third one (TAC-group) had excellent graft function and histology.

### Gene expression analysis revealed significant differences between the study groups

The RISET 2.0 custom microarray with 5,069 different probes, designed especially for transplantation research, was used to analyze the peripheral blood gene expression.

#### a) Early changes (W3): altered lineage composition as result of induction protocol and differential reconstitution between the groups

At first, pre-Tx and W3 samples were compared to evaluate the influence of the initial immunosuppressive treatment.

The 441 reporters differentially expressed between pre-Tx and W3 samples with an at least three-fold differential expression (S4 and S5 Tables) were subjected to an annotation enrichment analysis (S6 Table). As expected, markers related to T/B/NK cell lineages dominated the top-ranked differentially expressed genes, dropping from pre-Tx to W3 up to almost 500-fold. Interestingly, the IL-7 receptor, CCR7, and TCL1A genes that are strongly expressed on naïve lymphocytes, belonged to the top genes down-regulated by factor 492-, 189-, and 62-fold, respectively, suggesting a preferential reduction of naive compared to the memory/effector compartment).

At W3, after only one week of treatment allocation, 93 reporters were found to be upregulated and 151 downregulated in the comparison of TAC- vs. SIR -group (S7 and S8 Tables). Remarkably, the TAC -group revealed enhanced expression of B-cell related genes (*TCL1A*, *BLK)* and downregulation of inflammation related genes (*IL-8*, *MMP-8)* and dysregulation of those genes continued up to M12 (S9 and S10 Tables).

#### b) Patients on TAC-monotherapy had a tolerance-like signature

Then the samples taken at M2, M3, M6, and M12 were compared between the two study arms. The >1.5-fold differentially expressed genes found (S9 and 10 Tables) were subjected to an annotation enrichment analysis.

**Fig 6:**
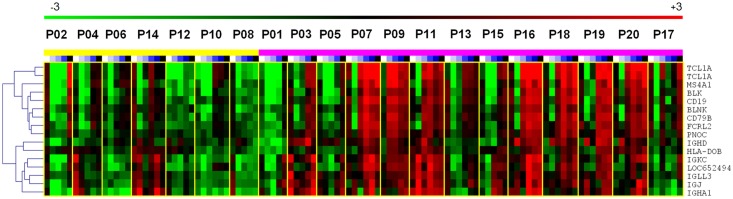
Heat map of B-cell specific genes differentially expressed between TAC (violet) and SIR-group (yellow) with stronger expression in TAC samples.

Most interestingly, the top genes highly expressed in the TAC-group belong to B cell (*CD79A*, *BLK*, *FCRL2*, *TCL1A*) and Ig-related categories (*IGHM*, *IGLC*, *IGKC*, *IGHG1*, *CD200*) (Fig 6, Table 3). The pattern is similar to the recently published signature in operational tolerant patients [31, 36–38]. This might indicate a faster regeneration of B cells in the TAC-group after the initial cell depletion.

**Table 3:** List of B cell associated genes found to be differentially expressed within the particular group comparison. Annotation data are from http://www.uniprot.org/ and http://www.genecards.org/. In Tacrolimus/Sirolimus comparison fold change was calculated from gene expression medians of all measured time-points in particular groups. In rejecting and non-rejecting patients only samples collected at later time points (M2, M3, M6 and M12) were used to calculate medians of gene expression.

Name	Median fold change			Description	Relevant features
	Tac x Sir	Rej x non-rej.	Pre-Tx x W3		
**TCL1A**	13.8	-4.8	62.9	T-cell leukemia/lymphoma 1A	Involved in B cell receptor pathway. Expressed in naive B cells more than in memory B cells. Overexpression of TCL1A prolongs naive B cell survival.
**IGHM**	7.9	-4.8	9.29	immunoglobulin heavy constant mu	Encodes the C region of the mu heavy chain of the IgM isotype. Expressed by naive B cells.
**CD79A**	6.6	-4.7	22.57	CD79a molecule, immunoglobulin-associated alpha	Required in cooperation with CD79B for initiation of the signal transduction cascade activated by binding of of antigen to the B-cell antigen receptor complex (BCR) which leads to antigen presentation. Also required for BCR surface expression and for efficient differentiation of pro- and pre-B-cells.
**IGLC**	6.2	-5.3	1.52	immunoglobulin lambda constant group	Encodes the Ig domain.
**CD200**	6.2	-6.3	3.21	CD200 molecule	Encodes the Ig domain.
**IGHG1IGHG2IGHG3IGHG4**	5.8	-3.3	1.06	immunoglobulin heavy constant gamma 1	Encodes the Ig domain.
**IGKC**	5.6	-3.9	2.67	immunoglobulin kappa constant	Encodes the Ig domain.
**BLK**	5.3	-3.7	10.98	B lymphoid tyrosine kinase	Encodes a nonreceptor tyrosine-kinase involved in B-cell receptor signaling.
**FCRL2**	5.2	-3.1	3.33	Fc receptor-like 2	Membrane protein belonging to FCRL family, expressed preferentially by memory B cells.
**IGHA1**	5.2	-4.3	1.28	immunoglobulin heavy constant alpha 1	Ig domain.
**MS4A1**	4.9	-5.0	15.9	membrane-spanning 4-domains, subfamily A, member 1	B-lymphocyte specific, cell-surface molecule involved in B cell activation and differentiation.
**CD19**	3.4	-3.9	4.27	CD19 molecule	Assembles with the antigen receptor of B-lymphocytes in order to decrease the threshold for antigen receptor-dependent stimulation.

Another interesting group of genes up-regulated in the TAC-group was described to be overexpressed on monocytes following IL-10 treatment [39] suggesting the occurrence of regulatory monocytes in this group (S11 Table).

Among the top genes up-regulated in the SIR-group were genes involved in inflammation *FN1* (fibronectin 1), *IL-8* (interleukin 8), *MMP-8* (matrix metallopeptidase 8), *TNNT1* (troponin T type 1), *C4A/C4B (*complement component 4A / 4B), and TGM2 (tissue transglutaminase) (S10 Table).

#### c) The special molecular signature of the TAC-group revealed by microarray could be validated by qRT-PCR in blood samples

Differential gene expressions revealed by microarray, were validated by qRT-PCR of 19 genes in blood samples. These genes were selected as highly up-regulated (*TCL1A*, *CD79A*, *CD200*, *MS4A1*, *FCRL2*, *HS3ST1* and *CCR7*) or down-regulated (*FN-1*, *IL-8*, *C4A*) in the TAC-group. *CD247*, *CCR2*, and *CCL2* were up-regulated in patients with graft rejection and proteinuria. *CCR5* and *FCRL1* genes were chosen as counterparts for CCR2 and FCRL2, respectively. In addition, we analyzed gene expression of recently described tolerance associated markers (*IRF5*, *TLR5*, *SLC8A1*, *SH2D1B* [31]).

**Fig 7:**
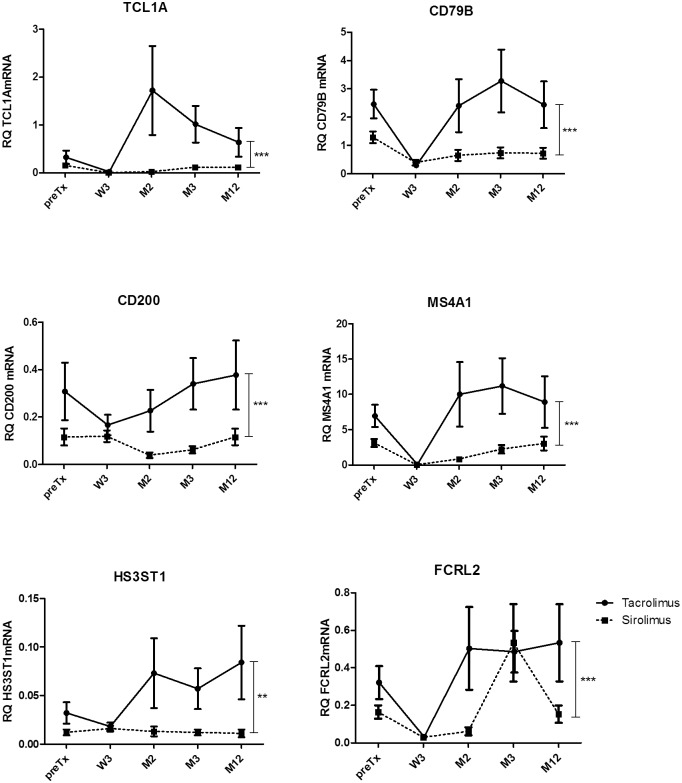
Validation of microarray analysis by qRT-PCR of blood samples. The comparison of TAC- and SIR- group of patients. All data are presented as mean±SEM. Statistically significant difference in gene expression within the whole study period was calculated by GLM mixed model. ** p<0.05, *** p<0.001.

In fact, qRT-PCR confirmed the statistically significant higher expression of B-cell related genes (*CD79B*, *FCRL1*, *FCRL2*, *MS4A1*, *TCL1A*), cytokine receptors *CCR5* and *CCR7*, *CD200*, the transcription factor *IRF5*, the toll-like receptor *TLR5*, *HS3ST1* (heparan sulfate 3-O-sulfotransferase 1), and *SH2D1B*, a gene playing a role in signal transduction of antigen-presenting cells in the TAC-group (Fig 7). Additionally, the relative up-regulation of FN1, a gene involved in fibrosis, was validated in the SIR-group.

Finally, the flow cytometric data confirm the faster B-cell recovery in TAC-group (Fig 4).

#### e) Lower intragraft expression of inflammation related genes confirm immune silence

**Fig 8:**
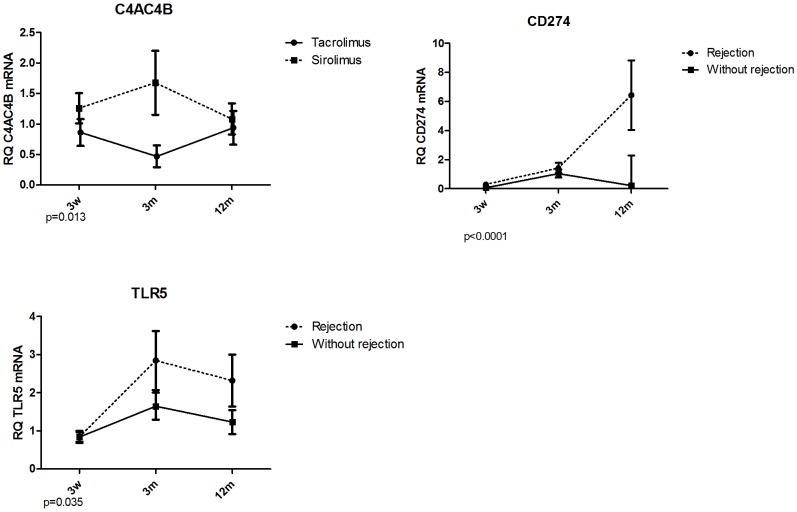
Validation of microarray analysis of blood samples by qRT-PCR of graft biopsies. The comparison of TAC- and SIR- group of patients and of patients with /without rejection event within 12 months posttransplant. All data are presented as mean±SEM. P values shown under the graphs indicate statistically significant difference in gene expression calculated by GLM mixed model.

The transcripts patterns from peripheral blood were further evaluated in protocol biopsies. Statistically significant differences in intrarenal gene expression were verified for down-regulation of *C4A/C4B* (part of complement cascade, mediator of inflammation) in the TAC-group (Fig 7). Moreover, non-rejecting grafts (mostly TAC-group) showed down-regulation of *CD274* (*PD1-L*) and *TLR5* (activated macrophages) compared to grafts with rejection within 12M (Fig 8).

## Discussion

Based on recent biomarker studies that revealed a key role of donor-specific Teff in relation to poor allograft outcome and need for high-dose multiple-drug immunosuppression[7–9], we designed a novel induction protocol that sequentially targets CD52 to reduce (alloreactive) clonal size and TNF to inhibit/destroy tmTNF-expressing donor-specific Teff recently (re)activated by the allograft. The PoC-trial confirms our hypothesis that this protocol might be a promising novel option to achieve safe monotherapy in almost all kidney transplant patients as early as post-d2, even in those with enhanced levels of pre-transplant T-cell sensitization. The excellent graft survival and function during 5yr follow-up in the TAC-group were associated with a particular biomarker profile: i) B-cell signature reported to be associated with operational tolerance [31, 36, 40], ii) almost no signs of inflammation in the graft and the blood, iii) some up-regulation of regulatory genes, and iv) control of humoral alloresponse in almost all patients. These data suggest that the short-term and sequential induction protocol followed by low/intermediate-dose tacrolimus monotherapy not only controls undesired donor-reactive alloresponsive T/B-cells but might also supports some regulation (Suppl. Tab. 3) allowing long-term minimized immunosuppression. Thus this protocol was also successful in most of the patients with enhanced T-cell presensitization that have commonly poorer outcome even under standard triple-drug immunosuppression.

Despite the very promising data in the TAC-group, the poor outcome of the SIR-group was very surprising. This study was originally planned to prove the superiority of SIR-monotherapy after dual induction therapy, alemtuzumab and infliximab, as an insufficient targeting of donor-reactive Teff has been discussed as reason for the high AMR rate reported in patients who received SIR-monotherapy after alemtuzumab induction [41]. Moreover, it was suggested that SIR support regulatory T cells [16]. However more late acute and chronic rejections occurred in SIR-group during the first 12 months of therapy. Frequent rejection and proteinuria were reasons to stop enrollment into this arm after first 7 patients. During the follow-up all patients of SIR-group were converted to standard immunosuppression because of abnormal histology along with side effects. It is known that sirolimus is less effective than calcineurin inhibitors (CNI) when given de-novo after kidney transplantation [42]. Similarly, other studies have shown higher rejection rate and poorer graft outcome if CNI-free regimen was used [43] despite better renal function in patients who continued with CNI-free immunosuppression. However, it is not clear why sirolimus monotherapy in our double-induction protocol looks worse compared to the combination with alemtuzumab alone as reported elsewhere [12, 28]. Here we could better control AMR but less good TCMR and proteinuria. We cannot rule out that particularly the latter might be related to the combination of targeting TNF and mTOR.

Most interestingly, the biomarker studies confirm the superiority of the TAC-group vs. the SIR-group at all parameters. The differences at the biomarker signatures were seen already long before the clinical picture of rejection and/or proteinuria in the SIR-group was visible. There is still the textbook statement in the air that CNI prevent tolerance induction. This is based on biased preclinical studies using non-physiologically high CNI blood level. Recently low-dose CNI was shown to support tolerance in a rat kidney transplant model [44]. Moreover, all three tolerance induction protocols (Boston, Stanford, Chicago) related to chimerism with donor hematopoietic stem cells use CNI for > 6 months to stabilize tolerance induction phase [10, 45, 46] and similarly kidney transplant patients who are free of rejection under CNI standard therapy exhibited a particular molecular signature [37]. Remarkably, our stable patients in the TAC-group developed a comparable signature.

As expected, alemtuzumab induction was initially associated with profound downregulation of many immune-related genes, however, as early as at W3 (one week after stratification into the two groups) differences between both treatment arms had become obvious. Especially B cell-related transcripts were significantly spared in TAC-group. It was shown that after initial B-cell depletion caused by alemtuzumab, peripheral B cells repopulate from W6 and after M6 even exceeded base line levels and result in long-term shift toward naïve/transitory B cells that have been associated with operational tolerance [47]. In this study we clearly showed, that this observation is true in tacrolimus treated patients while not in sirolimus patients. Similarly, our group [37] and later Heidt et al [48] have shown that expression levels of B-cell related markers *TCL1A*, *CD79B* and *MS4A1* were increased in stable and rejection-free patients but decreased at the time of acute rejection, respectively. Similarly, intrarenal B-cell signatures were observed in grafts with better control of acute rejection [49].

There are also some limitations of this study. Although the study was originally planned as open label randomized trial, due to methodological limitations in randomization, small sample size, and lack of power to assess the primary endpoint, it is scientifically correct to present our data as prospective observational trial. The failure to allocate last 7 patients to sirolimus treatment was only ethical one. Also-DSA monitoring clearly lacks pretransplant values as sera were not available.

Sirolimus inferiority was obvious and in line with others [42] and thus confirmatory larger prospective trial is not advocated. Contrary, the concept of tacrolimus monotherapy after profound depletive regimen should be further validated in a randomized multicenter trial as recently prepared by the BioDrim consortium.

In summary, our sequential double induction protocol followed by tacrolimus monotherapy showed very promising 5yr-outcome, even in T-cell presensitized patients in regards of particular biomarker profile of specific B-cell signature, other regulatory and tolerance-associated genes and inhibition of inflammation-related genes.

## Supporting Information

S1 Data Log files for analysis of gene expression data using GLMM model of SPSS. (DOCX)

S1 Fig Mean serum creatinine levels in study groups and standard of care group. Patients received induction treatment with Alemtuzumab followed by Infliximab and tacrolimus (TAC, n = 13) or sirolimus (SIR, n = 7) monotherapy. Standard of care group were patients with same inclusion criteria and from the same time period treated with CNI-based (tacrolimus or cyclosporine, mycophenolate mofetil and steroids) immunosuppression (control, n = 174).
(TIF)

S1 File Consort 2010 Checklist. (DOC)

S2 File IRB study protocol. (PDF)

S1 Table Change in immunosuppressive protocol within 60M follow up and comparison of graft function of kidney transplant recipients in TAC- and SIR-group evaluated by serum creatinine level. (DOCX)

S2 Table Tacrolimus and Sirolimus trough levels in individual patients during follow-up. (DOCX)

S3 Table Demographic and clinical variables in each of patients included in the study. (DOCX)

S4 Table List of genes significantly up-regulated in pre-Tx samples compared to samples at 3 weeks after Tx. Complete list of 262 probes ranked according to median fold change (only fold changes ≥3 were included) with corresponding p values (two-tailed t test) and microarray probe ID.
(DOCX)

S5 Table List of genes significantly down-regulated in pre-Tx samples compared to samples at 3 weeks after Tx. Complete list of 179 probes ranked according to median fold change (only fold changes ≥3 were included) with corresponding p values (two-tailed t test) and microarray probe ID.
(DOCX)

S6 Table Annotation enrichment of genes with significantly higher differential expression in pre-Tx samples compared with samples collected three weeks after Tx. Only the first 15 annotations with the highest statistical significance are shown.
(DOCX)

S7 Table List of genes significantly up-regulated in W3 samples of patients from Tacrolimus group compared to patients from Sirolimus group. Complete list of 93 probes ranked according to median fold change (only fold changes ≥1.5 were included) with corresponding p values (two-tailed t test) and microarray probe ID.
(DOCX)

S8 Table List of genes significantly down-regulated in W3 samples of patients from Tacrolimus group compared to patients from Sirolimus group. Complete list of 151 probes ranked according to median fold change (only fold changes ≥1.5 were included) with corresponding p values (two-tailed t test) and microarray probe ID.
(DOCX)

S9 Table List of genes significantly up-regulated in M2-M12 samples of patients from tacrolimus group compared to patients from Sirolimus group. Complete list of 134 probes ranked according to median fold change (only fold changes ≥1.5 were included) with corresponding p values (two-tailed t test) and microarray probe ID.
(DOCX)

S10 Table List of genes significantly down-regulated in M2-M12 samples of patients from tacrolimus group compared to patients from Sirolimus group. Complete list of 136 probes ranked according to median fold change (only fold changes ≥1.5 were included) with corresponding p values (two-tailed t test) and microarray probe ID.
(DOCX)

S11 Table Annotation enrichment analysis of genes with significantly higher differential expression in M2-M12 samples of patients treated with Tacrolimus relative to the Sirolimus group. Only the first 15 annotations with the highest statistical significance are shown.
(DOCX)

## Abbreviations

AMR: Antibody Mediated Rejection
BKV: BK Viremia
CMV: Cytomegalovirus
CNI: Calcineurin Inhibitors
d: day(s)
DSA: Donor Specific Antibodies
EBV: Epstein-Barr virus
i.v.: intravenous(ly)
M: month(s)
MMF: Mycophenolate Mofetil
PBMC: Peripheral Blood Mononuclear Cell
PCR: Polymerase Chain Reaction
PoC: Proof-of-Concept
POD: postoperative day
SIR: Sirolimus
TAC: Tacrolimus
Teff: T effector cell
TCMR: T Cell Mediated Rejection
tm: transmembrane
TNF: Tumor Necrosis Factor
Tx: Transplantation
W: week(s)
yr: year(s)
