# Supplementary material for: Sequential Targeting of CD52 and TNF Allows Early Minimization Therapy in Kidney Transplantation: From a Biomarker to Targeting in a Proof-Of-Concept Trial
Source: PLoS One. 2017 Jan 13;12(1):e0169624. doi: 10.1371/journal.pone.0169624 (PMC5234822; doi:10.1371/journal.pone.0169624)
Supplement: S1 Data — (DOCX) [file pone.0169624.s001.docx]

S1 Data. Log files for analysis of gene expression data using GLMM model of SPSS.

*Generalized Linear Mixed Models.

GENLINMIXED

/DATA_STRUCTURE SUBJECTS=ID REPEATED_MEASURES=odber COVARIANCE_TYPE=DIAGONAL

/FIELDS TARGET=CD79B TRIALS=NONE OFFSET=NONE

/TARGET_OPTIONS DISTRIBUTION=GAMMA LINK=LOG

/FIXED EFFECTS=odber*Lecba odber*rejekce odber*proteinurie Lecba rejekce proteinurie USE_INTERCEPT=TRUE

/BUILD_OPTIONS TARGET_CATEGORY_ORDER=ASCENDING INPUTS_CATEGORY_ORDER=ASCENDING MAX_ITERATIONS=100 CONFIDENCE_LEVEL=95 DF_METHOD=RESIDUAL COVB=MODEL

/EMMEANS TABLES=odber*Lecba COMPARE=Lecba CONTRAST=PAIRWISE

/EMMEANS TABLES=odber*rejekce COMPARE=rejekce CONTRAST=PAIRWISE

/EMMEANS TABLES=odber*proteinurie COMPARE=proteinurie CONTRAST=PAIRWISE

/EMMEANS TABLES=Lecba COMPARE=Lecba CONTRAST=PAIRWISE

/EMMEANS TABLES=rejekce COMPARE=rejekce CONTRAST=PAIRWISE

/EMMEANS TABLES=proteinurie COMPARE=proteinurie CONTRAST=PAIRWISE

/EMMEANS_OPTIONS SCALE=ORIGINAL PADJUST=LSD.
