# Supplementary figures and images for: Sequential Targeting of CD52 and TNF Allows Early Minimization Therapy in Kidney Transplantation: From a Biomarker to Targeting in a Proof-Of-Concept Trial
Source: PLoS One. 2017 Jan 13;12(1):e0169624. doi: 10.1371/journal.pone.0169624 (PMC5234822; doi:10.1371/journal.pone.0169624)

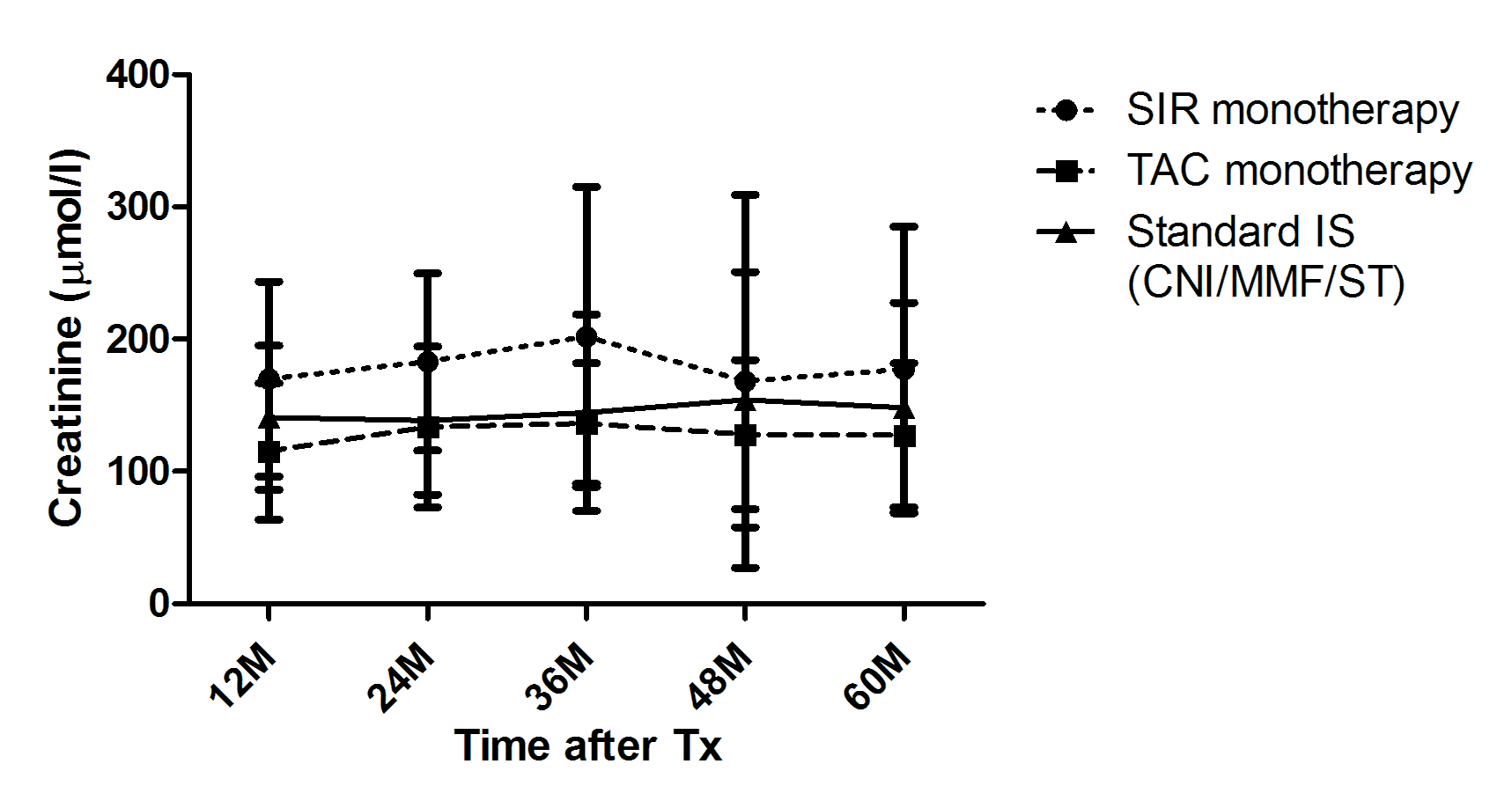

Supplement: S1 Fig — Patients received induction treatment with Alemtuzumab followed by Infliximab and tacrolimus (TAC, n = 13) or sirolimus (SIR, n = 7) monotherapy. Standard of care group were patients with same inclusion criteria and from the same time period treated with CNI-based (tacrolimus or cyclosporine, mycophenolate mofetil and steroids) immunosuppression (control, n = 174). (TIF) [file pone.0169624.s002.tif]
