# Supplementary material for: Sequential Targeting of CD52 and TNF Allows Early Minimization Therapy in Kidney Transplantation: From a Biomarker to Targeting in a Proof-Of-Concept Trial
Source: PLoS One. 2017 Jan 13;12(1):e0169624. doi: 10.1371/journal.pone.0169624 (PMC5234822; doi:10.1371/journal.pone.0169624)
Supplement: S1 Table — (DOCX) [file pone.0169624.s005.docx]

Supplemental Table S1 **.** Change in immunosuppressive protocol within 60M follow up and comparison of graft function of kidney transplant recipients in TAC- and SIR-group evaluated by serum creatinine level.

.

|  | Tacrolimus (n=13) | Sirolimus (n=7) |  |  |  |
| --- | --- | --- | --- | --- | --- |
| On therapy at |  |  |  |  |  |
| M3 | 13/13 (100%) | 6/7 (85.7%) |  |  |  |
| M6 | 12/13 (92.3%) | 5/6 (83.3%) |  |  |  |
| M12 | 13/13 (100%) | 3/6 (50%) |  |  |  |
| M24 | 13/13 (100%) | 1/6 (16.7%) |  |  |  |
| M36 | 12/13 (92.3%) | 0/5 (0%) |  |  |  |
| M60 | 11/12 (91.8%) | 0/5 (0%) |  |  |  |
| Creatinine (µmol/L), median [min; max] |  |  |  | P value |  |
| W3 | 188.8 [101; 321] | 180 [116; 263] |  | 0.251 |  |
| M3 | 137.9 [99; 295] | 142.6 [102; 168] |  | 0.322 |  |
| M12 | 110 [80; 200] | 134.2 [86; 271] |  | 0.161 |  |
| M36 | 120 [80; 212] | 180.3 [115; 339] |  | 0.054 |  |
| M48 | 128.4 [86; 230]) | 198.3 [81; 228] |  | 0.140 |  |
| M60 | 120.2 [70; 223] | 167.6 [103; 271] |  | 0.192 |  |

At M36, one patient from TAC-group developed chronic mixed TCMR/AMR rejection and due to nonadherence MMF was added. In SIR-group one patient was converted to tacrolimus/MMF combination due to oedema and skin lesion at M4. Three patients developed significant proteinuria and case biopsies revealed moderate to severe interstitial fibrosis and tubular atrophy along with chronic TCMR and glomerulitis, respectively. In these patients, the therapy was converted to tacrolimus/MMF combination at M6, M8 and M12, respectively. Two remaining patients from SIR-group were converted to tacrolimus/MMF at M13 and M33 due to chronic TCMR and severe oedema, respectively.
