# Supplementary material for: Sequential Targeting of CD52 and TNF Allows Early Minimization Therapy in Kidney Transplantation: From a Biomarker to Targeting in a Proof-Of-Concept Trial
Source: PLoS One. 2017 Jan 13;12(1):e0169624. doi: 10.1371/journal.pone.0169624 (PMC5234822; doi:10.1371/journal.pone.0169624)
Supplement: S2 Table — (DOCX) [file pone.0169624.s006.docx]

|  | Patient ID | P02 | P04 | P06 | P08 | P10 | P12 | P14 | P01 | P03 | P05 | P07 | P09 | P11 | P13 | P15 | P16 | P17 | P18 | P19 | P20 |
| --- | --- | --- | --- | --- | --- | --- | --- | --- | --- | --- | --- | --- | --- | --- | --- | --- | --- | --- | --- | --- | --- |
|  | **Group** | S | S | S | S | S | S | S | T | T | T | T | T | T | T | T | T | T | T | T | T |
| **TAC levels** | **D14** | 9.7 | 8.9 | 10.2 | 14.8 | 25.6 | 14.3 | 2.4 | 24.2 | 13.4 | 18 | 7.4 | 9.5 | 7.5 | 14.1 | 10.8 | 12 | 6.5 | 11.2 | 11.5 | 8.3 |
|  | **W3** | 8.4 |  |  |  |  |  |  | 6.5 | 4.7 | 22.5 | 6.1 | 13.9 | 10.7 | 20.7 | 3.4 | 9 | 4.2 | 9.6 | 10.7 | 4.3 |
|  | **M3** | 0 |  |  |  |  |  |  | 4.3 | 16.3 | 11.1 | 9.7 | 7.9 | 10.1 | 12.7 | 6.7 | 6.5 | 9.1 | 5.2 | 3.9 | 6.2 |
|  | **M6** |  |  |  |  |  | 5.9 |  | 5.9 | 7.1 | 5.9 | 7.4 | 4.5 | 9.2 | 5.2 | 6.8 | 10.6 | 6.5 | 3.6 | 2.9 |  |
|  | **M9** |  |  | 10.8 |  |  | 4.1 | 4.1 | 7.6 | 9.5 | 6.6 | 12.5 | 9.8 |  | 6 | 6.3 | 4.4 | 12.3 | 7.3 | 4.1 | 4 |
|  | **M12** |  |  | 11.4 | 18.3 |  | 5.4 | 5.8 | 9.5 | 7.6 | 7.6 | 9.2 | 5.9 | 9 | 7 | 12 | 6.6 | 10.4 | 6.7 | 7.9 | 5.5 |
|  | **M18** |  | 7.1 | 8.9 | 2.4 |  | 6.2 | 4.5 | 11.7 | 3.9 | 9.7 | 4.4 | 5.7 | 5.6 | 6.3 | 11.9 | 4.1 | 6.4 | 5.5 | 7 | 5 |
|  | **M24** |  | 5.6 | 9.7 | 2 |  | 2.4 | 4.4 | 4.2 | 4.5 | 8.3 | 5.3 | 5.8 | 5.9 | 2 | 8 | 4.2 | 6.6 | 4.8 | 4.2 | 4.8 |
|  | **M30** |  | 8.7 | 7.3 | 2.3 |  | 3 | 6.7 | 5.5 | 8.6 | 10.8 | 4.2 | 6.2 | 3.9 | 5.3 | 4.4 | 3.2 | 4.7 | 5.4 | 4.8 | 4.1 |
| **SIR levels** | **D14** |  |  |  |  |  |  |  |  |  |  |  |  |  |  |  |  |  |  |  |  |
|  | **W3** |  | 3.4 | 10.1 | 0 | 12 | 30 | 7.3 |  |  |  |  |  |  |  |  |  |  |  |  |  |
|  | **M3** |  | 6.2 |  | 8.6 | 8.4 | 13.1 | 6.5 |  |  |  |  |  |  |  |  |  |  |  |  |  |
|  | **M6** |  | 7.8 | 5.2 | 7.3 | 5.6 |  | 5.9 |  |  |  |  |  |  |  |  |  |  |  |  |  |
|  | **M9** |  | 6.5 |  | 9.1 | 8.4 |  |  |  |  |  |  |  |  |  |  |  |  |  |  |  |
|  | **M12** |  | 5.5 |  | 7.8 | 7.4 |  |  |  |  |  |  |  |  |  |  |  |  |  |  |  |
|  | **M18** |  |  |  |  | 8.6 |  |  |  |  |  |  |  |  |  |  |  |  |  |  |  |
|  | **M24** |  |  |  |  | 8.4 |  |  |  |  |  |  |  |  |  |  |  |  |  |  |  |
|  | **M30** |  |  |  |  | 8.4 |  |  |  |  |  |  |  |  |  |  |  |  |  |  |  |

**Target values for tacrolimus were 10-15 ng/ml until M3. then 5-10 ng/ml and for sirolimus 8-12 ng/kg until M3. then 4-8 ng/ml.*

Table S2. Tacrolimus and Sirolimus through levels in in individual patients during follow-up.
