# Supplementary material for: Sequential Targeting of CD52 and TNF Allows Early Minimization Therapy in Kidney Transplantation: From a Biomarker to Targeting in a Proof-Of-Concept Trial
Source: PLoS One. 2017 Jan 13;12(1):e0169624. doi: 10.1371/journal.pone.0169624 (PMC5234822; doi:10.1371/journal.pone.0169624)
Supplement: S3 Table — (DOCX) [file pone.0169624.s007.docx]

Supplemental Table S3 Demographic and clinical variables in each of patients included in the study.

n.d. not done, xxx-not performed due to graft failure or patients death

| **Patient** | | | **P02** | **P04** | **P06** | **P08** | **P10** | **P12** | **P14** | **P01** | **P03** | **P05** | **P07** | **P09** | **P11** | **P13** | **P15** | **P16** | **P17** | **P18** | **P19** | **P20** |
| --- | --- | --- | --- | --- | --- | --- | --- | --- | --- | --- | --- | --- | --- | --- | --- | --- | --- | --- | --- | --- | --- | --- |
| **Group** | | | **S** | **S** | **S** | **S** | **S** | **S** | **S** | **T** | **T** | **T** | **T** | **T** | **T** | **T** | **T** | **T** | **T** | **T** | **T** | **T** |
| **Recipient age, years** | | | 46 | 29 | 46 | 49 | 55 | 48 | 26 | 60 | 63 | 56 | 47 | 33 | 51 | 55 | 70 | 56 | 20 | 51 | 55 | 63 |
| **Donor age, years** | | | 59 | 60 | 50 | 53 | 41 | 62 | 45 | 54 | 50 | 57 | 63 | 53 | 38 | 51 | 20 | 54 | 46 | 26 | 54 | 58 |
| **HLA mismatch** | | | 3 | 3 | 3 | 3 | 3 | 2 | 3 | 4 | 3 | 3 | 3 | 2 | 3 | 3 | 1 | 4 | 3 | 1 | 3 | 2 |
| **Peak PRA** | | | 8 | 2 | 0 | 0 | 0 | 2 | 2 | 8 | 2 | 2 | 0 | 0 | 4 | 6 | 4 | 2 | 0 | 2 | 7 | 4 |
| **Cold ischemia (hours)** | | | 18,48 | 23,04 | 16,56 | 16,08 | 18,24 | 23,76 | 18,48 | 21,36 | 17,28 | 18,72 | 24,72 | 13,92 | 18,48 | 15,12 | 21,36 | 24,96 | 24,48 | 16,8 | 13,68 | 16,08 |
| **Creatinine** (µmol/L) | | **W3** | 576,2 | 262,9 | 143,8 | 323,4 | 116,3 | 139 | 216,3 | 198,1 | 101 | 163,5 | 233 | 190,6 | 132,4 | 117,4 | 252,3 | 188,8 | 320,8 | 142,1 | 127,3 | 124,5 |
|  |  | **M3** | 206,7 | 168,4 | 128,3 | 167 | 102,4 | 133,9 | 156,9 | 121 | 100,7 | 99 | 137,9 | 147 | 116,1 | 11,8 | 295,4 | 165,3 | 246,1 | 146,3 | 133 | 133,3 |
|  |  | **M12** | xxx | 145 | 123,5 | 249,8 | 86 | 143,8 | 271,1 | 99,5 | 79,8 | 93,2 | 129,1 | 124 | 94,6 | 187 | 110 | 173 | 200,2 | 95,8 | 111,3 | 105,7 |
|  |  | **M36** | xxx | 219,8 | 140,7 | xxx | 114,7 | 194,4 | 339 | 121,5 | 79,6 | 95,2 | 145,5 | 151,1 | 120,2 | 238,7 | 120,4 | 153,3 | 212,3 | 96,1 | 111 | 126,7 |
|  |  | **M48** | xxx | 185,1 | 211,6 | xxx | 80,7 | 412,9 | 228 | 127,7 | 110,2 | 86,9 | 128,4 | 164,2 | 135,8 | xxx | 102,4 | 171,9 | 230,3 | 85,7 | 130,1 | 187,8 |
|  |  | **M60** | xxx | 198,2 | 137,0 | xxx | 102,7 | xxx | 271,2 | 122,6 | 70,2 | 89,6 | 141,1 | 152,1 | 120,2 | xxx | 98,6 | 165,7 | 222,5 | 98,4 | 119,1 | xxx |
| **Proteinuria** (g/24 hours) | | **W3** | 0,20 | 0,77 | 0,52 | 0,48 | 0,34 | 0,26 | 1,75 | 0,34 | 0,50 | 0,12 | 0,34 | 0,20 | n.d. | 0,61 | 0,76 | 0,25 | n.d. | n.d. | 0,53 | 0,18 |
|  |  | **M3** | 0,33 | 0,33 | 7,09 | 0,22 | n.d. | 0,2 | 0,46 | 0,33 | 0,78 | n.d. | 0,10 | 0,09 | 0,15 | 0,39 | 0,28 | 0,11 | 0,25 | 0,1 | 0,09 | 0,07 |
|  |  | **M12** | xxx | 3,84 | 4,75 | 0,3 | 0,22 | 0,3 | 4,87 | 0,37 | 0,23 | 0,23 | 0,22 | 0,24 | 0,39 | 0,39 | 0,14 | 0,16 | 0,4 | n.d. | 1,04 | n.d. |
|  |  | **M36** | xxx | 0,47 | 1,07 | xxx | 4,83 | 0,83 | 0,44 | 0,23 | 0,68 | 0,24 | n.d. | 0,46 | 0,24 | 4,64 | n.d. | n.d. | n.d. | n.d. | n.d. | 2,28 |
|  |  | **M48** | xxx | 5,28 | 0,36 | xxx | 1,32 | n.d. | 0,34 | 0,23 | 0.88 | 0,26 | n.d. | 2,12 | 0,21 | xxx | 0,21 | n.d. | 0,68 | n.d. | n.d. | 11,46 |
|  |  | **M60** | xxx | 1,72 | 2,27 | xxx | n.d. | xxx | 0,38 | 0,33 | 1,5 | 0,26 | n.d. | 1,08 | n.d. | xxx | n.d. | n.d. | n.d. | n.d. | 0,44 | xxx |
| **IP10** (pg/ml) | | **W3** | 40,0 | 93,9 | 40,0 | 20095,7 | 20,0 | 20,0 | 20,0 | 40,0 | 618,2 | 40,0 | 40,0 | 147,3 | 20,0 | 20,0 | 164,6 | 153,5 | 59,9 | 233,0 | 40,0 | 40,0 |
|  |  | **M2** | 141,5 | 176,5 | 570,5 | 490,5 | 20,0 | 20,0 | 2548,1 | 40,0 | 97,8 | 40,0 | 144,3 | 20,0 | 20,0 | 145,3 | 427,8 | n.d. | 893,0 | 40,0 | 286,8 | 143,8 |
|  |  | **M3** | 81,0 | 769,2 | 395,9 | 538,5 | 20,0 | 40,0 | 747,8 | 40,0 | 113,3 | 40,0 | 40,0 | 42,5 | 40,0 | 40,0 | 194,4 | 40,0 | 258,2 | 40,0 | 1322,4 | 128,0 |
| **Pre-transplant IFN-γ Elispot** | | | neg. | neg. | neg. | >1000 | 740 | neg. | n.d. | 568 | 163 | n.d. | neg. | neg. | 31 | n.d. | neg. | 538 | neg. | neg. | neg. | 121 |
| **Graft failure (postTx months)** | | | 4 |  |  |  |  | 56 |  |  |  |  |  |  |  | 46 |  |  |  |  |  |  |
| **Death (postTx months)** | | |  |  |  | 35 |  |  |  |  |  |  |  |  |  |  |  |  |  |  |  | 48 |
| **Case biopsy** | **4POD-M3** | | ATCMR IIA, AMR, ATN | ATN | ATN | ATN | ATN | ATN |  | AMR, mild ATN, transfer of nefroangiosclerosis, drug toxitis | ATN |  | ATN |  |  |  |  | ATN |  | ATN, BL | Marginal sample, normal | Arteriolopathy |
| **Protocol biopsy** | **W3** | | weighty ATN | ATN | normal | ATN | normal | normal | ATN | ATN,transfer of nefroangiosclerosis | CAN II,insudat vaskulopatie, tx glomerulopathy | ATN | mild ATN | x | ATN | ATN | ATN | normal | normal | IF/TA 1 | normal | focal mild fibrosis |
|  | **M3** | | normal | ATN | BL changes | ATN | normal | normal | normal | n.d. | mild ATN, nephroangiosclerosis | normal | mild ATN | normal | normal | TCMR IB /AMR | normal | normal | BL changes, IF/TA2 | IF/TA 1 | normal | IF/TA 1 |
|  | **M12** | | n.d. | IF/TA 3 grade,BL changes | BL changes | Chronic TCMR, mild ATN, IF/TA 2 | IF/TA 1 grade | marginal sample, normal | n.d. | chronic AMR | nephroangiosclerosis | normal | IgA GN reccurence | IgA GN reccurence | IF/TA 1 grade | Chronic, active TCMR, IF/TA I | marginal sample | IF/TA I | BL changes, IF/TA1 | BL changes | BL changes | IF/TA 1 |
| **Case biopsy** | **M6-12** | |  |  | Chronic AMR,BL changes with glomerulitis | BL |  |  | Chronic TCMR, IgA glomerulonephritis |  |  |  |  |  |  | ATN,probably bacterial etiol. |  | BL changes, IF/TA2 |  |  |  |  |
|  | **M12-M60** | |  | chronic TCMR, IgA reccurence |  |  | chronic AMR | chronic/active TCMR |  |  |  |  |  |  |  |  |  |  |  |  |  | chronic AMR |
