# Supplementary material for: Sequential Targeting of CD52 and TNF Allows Early Minimization Therapy in Kidney Transplantation: From a Biomarker to Targeting in a Proof-Of-Concept Trial
Source: PLoS One. 2017 Jan 13;12(1):e0169624. doi: 10.1371/journal.pone.0169624 (PMC5234822; doi:10.1371/journal.pone.0169624)
Supplement: S4 Table — Complete list of 262 probes ranked according to median fold change (only fold changes ≥3 were included) with corresponding p values (two-tailed t test) and microarray probe ID. (DOCX) [file pone.0169624.s008.docx]

| Supplemental Table S4.List of genes significantly up-regulated in pre-Tx samples compared to samples at 3 weeks after Tx. Complete list of 262 probes ranked according to median fold change (only fold changes ≥3 were included) with corresponding p values (two-tailed t test) and microarray probe ID. | | | | |
| --- | --- | --- | --- | --- |
| **Rank** | **Gene Name** | **Probe ID** | **p** | **fold change** |
| 1 | IL7R | A_23_P404494_riset1 | 9,03E-14 | 492,37 |
| 2 | CCR7 | A_23_P343398_riset1 | 1,33E-24 | 188,76 |
| 3 | TRA@ | A_23_P258504_riset1 | 2,19E-20 | 74,69 |
| 4 | TCF7 | A_23_P7582_riset1 | 1,27E-21 | 73,32 |
| 5 | TCL1A | MIL_PPPID394307780_riset1 | 1,34E-09 | 62,09 |
| 6 | CD3D | MIL_PPPID394307868_riset1 | 2,80E-10 | 56,96 |
| 7 | NELL2 | A_23_P10025_riset1 | 4,73E-17 | 46,11 |
| 8 | ANK3 | A_32_P33304_riset1 | 1,06E-20 | 39,35 |
| 9 | CD3G | CD3G_riset2 | 7,73E-14 | 38,08 |
| 10 | LEF1 | A_23_P213045_riset1 | 4,55E-20 | 34,35 |
| 11 | ZNF683 | A_23_P318380_riset1 | 3,53E-15 | 28,75 |
| 12 | ANK3 | A_23_P202269_riset1 | 2,26E-16 | 27,21 |
| 13 | CD28 | A_23_P91095_riset1 | 1,31E-17 | 26,69 |
| 14 | TRAT1 | A_23_P212568_riset1 | 3,10E-12 | 26,56 |
| 15 | KRT72 | A_32_P48825_riset2 | 2,15E-10 | 23,91 |
| 16 | DPP4 | A_23_P39885_riset1 | 3,59E-15 | 22,99 |
| 17 | FAM135A;FAM135B | A_24_P280465_riset1 | 3,68E-16 | 22,73 |
| 18 | CD79A | CD79A_riset2_piqor | 6,68E-13 | 22,57 |
| 19 | AQP3 | A_23_P112481_riset1 | 1,54E-10 | 22,33 |
| 20 | CD6 | A_23_P311875_riset1 | 3,30E-15 | 21,45 |
| 21 | TCL1A | A_23_P357717_riset1 | 7,00E-09 | 19,54 |
| 22 | FCRL5 | A_23_P201211_riset1 | 9,91E-12 | 18,50 |
| 23 | IL23A | A_23_P76078_riset1 | 1,96E-13 | 18,46 |
| 24 | VMO1 | A_23_P55356_riset1 | 4,85E-10 | 17,08 |
| 25 | LOC100129122 | A_32_P231493_riset1 | 9,22E-16 | 16,96 |
| 26 | PLCG1 | MIL_PPPID397416315_riset1 | 1,03E-10 | 16,87 |
| 27 | MS4A1 | MIL_PPPID394453510_riset1 | 5,44E-08 | 15,88 |
| 28 | KIF5C | A_32_P154473_riset1 | 1,69E-21 | 15,51 |
| 29 | GZMK | A_23_P156216_riset1 | 1,11E-11 | 15,15 |
| 30 | CD52 | A_23_P85800_riset1 | 6,11E-13 | 15,07 |
| 31 | MDGA1 | A_23_P310460_riset1 | 8,72E-05 | 15,07 |
| 32 | CD2 | A_23_P161076_riset1 | 5,38E-15 | 14,58 |
| 33 | ICOS | MIL_PPPID399200278_riset1 | 2,91E-18 | 14,44 |
| 34 | CTLA4 | MIL_PPPID394453530_riset1 | 4,29E-15 | 14,22 |
| 35 | CD8A | A_23_P68110_riset1 | 2,54E-10 | 14,09 |
| 36 | CXCR3 | A_23_P114299_riset1 | 3,33E-12 | 13,79 |
| 37 | CD5 | A_24_P364221_riset1 | 8,85E-09 | 13,66 |
| 38 | CCR6 | A_24_P234921_riset1 | 3,25E-19 | 13,32 |
| 39 | AMIGO1 | A_24_P302506_riset1 | 8,68E-17 | 12,12 |
| 40 | THC2385462 | A_32_P137819_riset1 | 2,91E-15 | 11,62 |
| 41 | FCGBP | A_23_P21495_riset1 | 1,05E-11 | 11,29 |
| 42 | BLK | BLK_riset2 | 1,15E-11 | 10,98 |
| 43 | IGFBP3 | A_23_P215634_riset1 | 2,52E-11 | 10,90 |
| 44 | KIAA0888 | A_23_P423074_riset1 | 1,60E-08 | 10,44 |
| 45 | IL2RA | A_24_P230563_riset1 | 2,11E-09 | 10,08 |
| 46 | MAL | MIL_PPPID394307800_riset1 | 3,04E-08 | 9,88 |
| 47 | AF116678 | A_24_P927090_riset1 | 3,15E-12 | 9,83 |
| 48 | SLAMF1 | SLAMF1_riset2 | 3,37E-12 | 9,63 |
| 49 | IGHM | A_24_P417352_riset1 | 2,51E-09 | 9,29 |
| 50 | TNFRSF25 | TNFRSF25_riset2 | 4,62E-19 | 9,25 |
| 51 | COL6A2 | MIL_PPPID394307828_riset1 | 5,00E-08 | 9,24 |
| 52 | RASGRP1 | A_23_P124642_riset1 | 2,47E-13 | 8,88 |
| 53 | IL2RA | A_23_P127288_riset1 | 1,40E-08 | 8,58 |
| 54 | DTX1 | A_24_P290751_riset1 | 3,87E-18 | 8,54 |
| 55 | LY9 | MIL_PPPID397416255_riset1 | 2,58E-12 | 8,25 |
| 56 | AA789123 | A_32_P485325_riset1 | 2,51E-09 | 8,19 |
| 57 | THC2391454 | A_32_P62371_riset1 | 5,80E-09 | 8,19 |
| 58 | KLK1 | A_23_P16252_riset1 | 6,51E-09 | 8,09 |
| 59 | LCK | LCK_riset2 | 1,27E-13 | 7,93 |
| 60 | CD96 | A_23_P44154_riset1 | 8,91E-09 | 7,92 |
| 61 | IL32 | A_23_P15146_riset1 | 2,33E-16 | 7,64 |
| 62 | KLRG1 | A_23_P64898_riset1 | 2,76E-14 | 7,45 |
| 63 | C6ORF105 | A_23_P156826_riset1 | 2,96E-12 | 7,39 |
| 64 | CD79B | A_23_P207201_riset1 | 1,06E-14 | 7,14 |
| 65 | FLT3LG | A_23_P78742_riset1 | 2,94E-18 | 6,99 |
| 66 | IGLV3-19 | A_24_P161764_riset1 | 4,72E-07 | 6,94 |
| 67 | BEX5 | A_32_P69849_riset1 | 3,66E-15 | 6,85 |
| 68 | ABLIM1 | A_23_P202520_riset1 | 1,75E-19 | 6,73 |
| 69 | TRBV20-1;TRBV20OR9-2 | A_23_P399604_riset1 | 3,20E-10 | 6,67 |
| 70 | GPR174 | A_23_P11070_riset1 | 8,32E-12 | 6,64 |
| 71 | GATA3 | A_23_P75056_riset1 | 4,03E-14 | 6,62 |
| 72 | GPR171 | A_32_P442899_riset1 | 9,35E-14 | 6,54 |
| 73 | IL28RA | IL28RA_riset2 | 2,54E-10 | 6,49 |
| 74 | NR3C2 | A_23_P392470_riset2 | 6,76E-09 | 6,44 |
| 75 | CD22 | CD22_riset2 | 2,09E-08 | 6,43 |
| 76 | TRAF5 | TRAF5_riset2 | 1,53E-08 | 6,32 |
| 77 | SLC41A1 | A_24_P122732_riset1 | 5,55E-10 | 6,29 |
| 78 | RORA | RORA_riset2 | 1,94E-11 | 6,06 |
| 79 | BCL2 | MIL_PPPID394453506_riset1 | 1,56E-10 | 6,01 |
| 80 | THC2438936 | A_32_P71876_riset1 | 1,79E-07 | 5,95 |
| 81 | PTPRCAP | A_23_P98173_riset1 | 3,47E-13 | 5,90 |
| 82 | CD40LG | A_23_P62220_riset1 | 7,17E-14 | 5,83 |
| 83 | LAT | A_23_P44105_riset1 | 1,85E-17 | 5,82 |
| 84 | RPS21 | A_23_P120660_riset1 | 1,57E-11 | 5,76 |
| 85 | RPL35;LOC390876;LOC440737;HCG_1983332 | A_24_P187023_riset1 | 1,07E-11 | 5,76 |
| 86 | IKZF3 | A_23_P376060_riset1 | 2,52E-07 | 5,74 |
| 87 | THC2374442 | A_32_P56037_riset1 | 3,73E-09 | 5,74 |
| 88 | TDRKH | A_23_P46351_riset1 | 1,42E-09 | 5,72 |
| 89 | AGMAT | A_24_P936171_riset1 | 3,81E-05 | 5,68 |
| 90 | TRGV9 | TRGV9_riset2 | 2,07E-08 | 5,68 |
| 91 | C12ORF57 | A_23_P350551_riset1 | 1,35E-16 | 5,66 |
| 92 | EDG1 | A_23_P160117_riset1 | 4,63E-14 | 5,66 |
| 93 | ITK | ITK_riset2 | 4,22E-13 | 5,63 |
| 94 | WDR86 | A_32_P138396_riset1 | 8,55E-10 | 5,62 |
| 95 | FHIT | FHIT_riset2 | 1,58E-09 | 5,55 |
| 96 | HCG_1984468;LOC388524;RPSA | MIL_PPPID399806188_riset1 | 3,56E-14 | 5,52 |
| 97 | CDKN1C | A_32_P78613_rev_riset1 | 4,31E-11 | 5,51 |
| 98 | AF269286 | A_24_P136379_riset1 | 1,33E-09 | 5,47 |
| 99 | PLEKHA1 | A_23_P115792_riset1 | 3,97E-11 | 5,32 |
| 100 | LAG3 | A_23_P116942_riset1 | 1,53E-09 | 5,24 |
| 101 | B3GNT1 | A_23_P86900_riset1 | 2,13E-10 | 5,23 |
| 102 | TBC1D4 | A_23_P88095_riset1 | 3,58E-13 | 5,22 |
| 103 | TRA@ | MIL_PPPID399200166_riset1 | 8,85E-07 | 5,18 |
| 104 | CDKN1C | A_23_P428129_riset1 | 2,90E-12 | 5,18 |
| 105 | ALDH8A1 | A_23_P214944_riset1 | 3,79E-05 | 5,16 |
| 106 | HLA-DOA | A_32_P356316_riset1 | 8,51E-10 | 5,15 |
| 107 | HLF | A_23_P356585_riset1 | 1,79E-10 | 5,06 |
| 108 | ACVRL1 | A_24_P945113_riset1 | 9,87E-05 | 5,03 |
| 109 | MYBL1 | A_24_P308139_riset1 | 5,52E-07 | 5,02 |
| 110 | C17ORF69 | A_24_P305038_riset1 | 1,21E-10 | 5,01 |
| 111 | ROBO3 | A_23_P356581_riset1 | 6,06E-12 | 4,99 |
| 112 | PVRL3 | A_23_P401547_riset1 | 5,97E-12 | 4,98 |
| 113 | BC011455 | A_24_P918891_riset1 | 7,13E-12 | 4,97 |
| 114 | EMR4 | MIL_PPPID399200170_riset1 | 5,39E-03 | 4,95 |
| 115 | RPS27 | A_23_P74629_riset1 | 1,25E-08 | 4,91 |
| 116 | CFH;CFHR1 | A_23_P114740_riset1 | 4,31E-08 | 4,87 |
| 117 | CHI3L2 | A_23_P12082_riset1 | 2,04E-04 | 4,79 |
| 118 | RHOH | A_23_P58132_riset1 | 6,55E-14 | 4,77 |
| 119 | PURA | MIL_PPPID394307936_riset1 | 1,07E-09 | 4,68 |
| 120 | TGFB3 | A_23_P88404_riset1 | 2,93E-09 | 4,67 |
| 121 | LDHB | A_23_P53476_riset1 | 6,03E-12 | 4,67 |
| 122 | C5ORF39 | A_23_P431591_riset1 | 2,43E-12 | 4,65 |
| 123 | PIK3IP1 | A_24_P134488_riset1 | 3,39E-09 | 4,64 |
| 124 | PCNXL2 | A_23_P200260_riset1 | 1,17E-10 | 4,58 |
| 125 | STAG3 | A_23_P145657_riset1 | 8,95E-15 | 4,55 |
| 126 | SCARNA17 | A_32_P37592_riset1 | 7,19E-12 | 4,52 |
| 127 | AFF3 | A_23_P373464_riset1 | 6,17E-09 | 4,50 |
| 128 | CCR4 | A_23_P72989_riset1 | 5,37E-11 | 4,50 |
| 129 | PRKCQ | A_23_P1374_riset1 | 4,25E-13 | 4,49 |
| 130 | OCIAD2 | A_23_P121702_riset1 | 2,82E-18 | 4,48 |
| 131 | MYBL1 | A_24_P367227_riset1 | 2,29E-06 | 4,47 |
| 132 | STARD9 | A_32_P130641_riset1 | 4,53E-07 | 4,47 |
| 133 | WDR54 | A_23_P68072_riset1 | 5,78E-17 | 4,46 |
| 134 | MGC24039 | A_23_P366559_riset1 | 9,35E-06 | 4,43 |
| 135 | AK000144 | A_24_P459522_riset1 | 1,99E-05 | 4,39 |
| 136 | LTBP3 | A_32_P72340_riset1 | 8,80E-14 | 4,38 |
| 137 | ENST00000360623 | A_24_P24053_riset1 | 1,04E-03 | 4,33 |
| 138 | SLC30A4 | A_32_P21255_riset1 | 2,27E-11 | 4,31 |
| 139 | CD19 | CD19_riset2 | 2,75E-07 | 4,27 |
| 140 | BTLA | MIL_PPPID399200354_riset1 | 2,06E-07 | 4,27 |
| 141 | AUTS2 | A_23_P122906_riset1 | 2,35E-07 | 4,26 |
| 142 | GATM | A_23_P129064_riset1 | 2,53E-03 | 4,24 |
| 143 | CTSF | A_23_P24433_riset1 | 1,90E-13 | 4,23 |
| 144 | UBE2CBP | A_24_P626931_riset1 | 3,01E-10 | 4,23 |
| 145 | EBF1 | A_32_P197561_riset1 | 3,38E-08 | 4,22 |
| 146 | ZBTB32 | A_23_P131024_riset1 | 2,11E-06 | 4,21 |
| 147 | RPL14 | A_23_P18294_riset1 | 3,21E-17 | 4,18 |
| 148 | RCAN2 | A_32_P156851_riset1 | 9,14E-05 | 4,18 |
| 149 | IL21R | IL21R_riset2 | 2,16E-12 | 4,18 |
| 150 | HLA-DQA2 | A_23_P42302_riset1 | 3,81E-02 | 4,15 |
| 151 | RPS6 | A_23_P123563_riset1 | 2,95E-11 | 4,15 |
| 152 | MTA3 | A_23_P411431_riset1 | 2,70E-17 | 4,12 |
| 153 | BLNK | A_24_P64344_riset1 | 4,94E-07 | 4,10 |
| 154 | ZNF573 | A_23_P339079_riset1 | 1,68E-18 | 4,09 |
| 155 | PTCH1 | PTCH1_riset2 | 1,10E-06 | 3,96 |
| 156 | IQCK | A_23_P324523_riset1 | 3,78E-05 | 3,96 |
| 157 | KIF21A | A_24_P366315_riset1 | 3,33E-10 | 3,94 |
| 158 | HOMER1 | A_23_P41917_riset1 | 3,32E-09 | 3,94 |
| 159 | FLJ11171 | A_24_P410378_riset1 | 1,01E-05 | 3,91 |
| 160 | CYORF15B | A_24_P307993_riset1 | 8,60E-03 | 3,90 |
| 161 | SAMD3 | A_23_P397937_riset1 | 1,97E-05 | 3,90 |
| 162 | TYW1 | A_24_P883109_riset1 | 1,91E-04 | 3,88 |
| 163 | STAT4 | A_23_P68031_riset1 | 6,24E-10 | 3,88 |
| 164 | CAND1 | A_24_P93656_riset1 | 8,50E-07 | 3,88 |
| 165 | ZAP70 | ZAP70_riset2 | 4,66E-09 | 3,88 |
| 166 | LOC441641 | A_24_P307443_riset1 | 2,20E-07 | 3,86 |
| 167 | WFS1 | A_23_P121499_riset1 | 1,33E-07 | 3,85 |
| 168 | FCER2 | A_23_P164773_riset1 | 2,64E-05 | 3,81 |
| 169 | MLL | A_24_P281913_riset1 | 4,79E-15 | 3,79 |
| 170 | CCR5 | A_23_P412321_riset1 | 2,34E-10 | 3,78 |
| 171 | SFTPD | A_23_P46785_riset1 | 2,43E-06 | 3,76 |
| 172 | THC2440435 | A_32_P230398_riset1 | 1,96E-06 | 3,76 |
| 173 | IFFO2 | A_23_P418031_riset1 | 2,50E-14 | 3,72 |
| 174 | CD70 | A_23_P119202_riset1 | 1,68E-08 | 3,70 |
| 175 | DHX33 | A_23_P130141_riset1 | 1,31E-03 | 3,65 |
| 176 | PTPN13 | A_23_P18493_riset1 | 3,23E-10 | 3,64 |
| 177 | SPTBN1 | A_23_P339095_riset1 | 2,19E-07 | 3,63 |
| 178 | KANK1 | A_23_P500130_riset1 | 2,98E-04 | 3,63 |
| 179 | C21ORF122 | A_23_P143514_riset1 | 4,42E-09 | 3,60 |
| 180 | RPL9 | A_32_P153100_riset1 | 2,38E-06 | 3,59 |
| 181 | LINGO2 | A_23_P157926_riset1 | 1,04E-02 | 3,59 |
| 182 | PTPLAD1;LOC732402 | A_23_P99927_riset1 | 1,08E-14 | 3,58 |
| 183 | RPL23 | A_23_P26713_riset1 | 1,20E-06 | 3,56 |
| 184 | DOCK3 | A_24_P153643_riset1 | 3,63E-07 | 3,56 |
| 185 | PYHIN1 | A_23_P365834_riset1 | 8,10E-05 | 3,55 |
| 186 | STAT4 | STAT4_riset2_piqor | 2,38E-09 | 3,54 |
| 187 | LOC645638 | A_24_P691826_riset1 | 3,00E-08 | 3,51 |
| 188 | THC2317111 | A_32_P82218_riset1 | 1,99E-05 | 3,49 |
| 189 | C14ORF56 | A_23_P413991_riset1 | 5,98E-06 | 3,48 |
| 190 | EEF1A1;EEF1AL7 | MIL_PPPID399806198_riset1 | 6,55E-10 | 3,46 |
| 191 | TUBE1 | A_23_P145053_riset1 | 6,38E-07 | 3,46 |
| 192 | PRSS23 | A_24_P937405_riset1 | 4,97E-03 | 3,44 |
| 193 | KIAA1641 | A_23_P253622_riset1 | 2,85E-10 | 3,43 |
| 194 | BI836739 | A_32_P194704_riset1 | 2,31E-08 | 3,43 |
| 195 | ADAMTS4 | A_23_P360754_riset1 | 9,65E-07 | 3,42 |
| 196 | EBI2 | A_23_P25566_riset1 | 3,75E-13 | 3,41 |
| 197 | IGKV3-20 | A_23_P21800_riset1 | 9,34E-03 | 3,41 |
| 198 | CARD11 | A_23_P82324_riset1 | 2,40E-07 | 3,41 |
| 199 | PCDH9 | A_24_P187218_riset1 | 4,67E-08 | 3,40 |
| 200 | C6ORF108 | A_23_P167951_riset1 | 4,15E-11 | 3,39 |
| 201 | RAD50 | A_24_P226198_riset1 | 2,35E-06 | 3,38 |
| 202 | PDCD7 | A_24_P159181_riset1 | 6,26E-15 | 3,38 |
| 203 | FANK1 | A_23_P115785_riset1 | 1,50E-04 | 3,38 |
| 204 | NR4A1 | A_23_P128230_riset1 | 6,55E-05 | 3,36 |
| 205 | LAX1 | A_24_P291278_riset1 | 3,12E-13 | 3,36 |
| 206 | RTTN | A_24_P108301_riset1 | 9,44E-08 | 3,35 |
| 207 | ZBTB10 | A_24_P64071_riset1 | 9,57E-06 | 3,33 |
| 208 | TRIM16L | A_24_P8304_riset1 | 1,56E-09 | 3,33 |
| 209 | FCRL2 | A_23_P160751_riset1 | 3,69E-06 | 3,33 |
| 210 | CLCF1 | A_23_P138760_riset1 | 3,79E-07 | 3,32 |
| 211 | FYN | MIL_PPPID402610592_riset2 | 1,75E-14 | 3,31 |
| 212 | NR1D1 | A_23_P420873_riset1 | 1,31E-12 | 3,31 |
| 213 | LOC283788 | A_24_P497437_riset1 | 5,28E-09 | 3,31 |
| 214 | TYW3 | A_24_P396327_riset1 | 5,12E-08 | 3,31 |
| 215 | DJ222E13.2 | A_24_P365679_riset1 | 2,45E-02 | 3,30 |
| 216 | C1QA | C1QA_riset2 | 7,33E-06 | 3,28 |
| 217 | C10ORF2 | A_24_P258073_riset1 | 1,03E-08 | 3,27 |
| 218 | HLTF_readthrough_after_exon21 | MIL_PPPID394307771_riset1 | 3,26E-07 | 3,26 |
| 219 | HS3ST1 | A_23_P121657_riset1 | 5,84E-07 | 3,25 |
| 220 | BCKDHB | A_23_P93464_riset1 | 3,32E-05 | 3,25 |
| 221 | OXCT1 | A_32_P63848_riset1 | 7,23E-07 | 3,25 |
| 222 | CKB | A_23_P25674_riset1 | 1,62E-08 | 3,24 |
| 223 | THC2378994 | A_32_P117666_riset1 | 5,41E-10 | 3,22 |
| 224 | CDC23 | A_24_P913227_riset1 | 2,06E-10 | 3,21 |
| 225 | AK025360 | A_24_P674443_riset1 | 2,89E-06 | 3,21 |
| 226 | EBF1 | A_24_P156501_riset1 | 2,46E-05 | 3,21 |
| 227 | CD200 | A_23_P121480_riset1 | 1,28E-02 | 3,21 |
| 228 | RPS27A | MIL_PPPID397416175_riset1 | 7,68E-09 | 3,20 |
| 229 | ITGB7 | ITGB7_riset2_piqor | 1,66E-08 | 3,19 |
| 230 | LOC100129637 | A_24_P8075_riset1 | 7,43E-10 | 3,18 |
| 231 | C1QA | C1QA_riset2_piqor | 1,08E-04 | 3,18 |
| 232 | POLRMT | A_23_P131096_riset1 | 5,23E-12 | 3,17 |
| 233 | SPTAN1 | A_23_P20832_riset1 | 1,68E-13 | 3,17 |
| 234 | TMEM30B | A_32_P129752_riset1 | 1,58E-06 | 3,17 |
| 235 | PRPF19 | A_23_P52978_riset1 | 1,61E-09 | 3,16 |
| 236 | NDUFA5 | A_23_P412041_riset1 | 4,88E-04 | 3,15 |
| 237 | HLCS | A_23_P304991_riset1 | 1,28E-12 | 3,15 |
| 238 | ALMS1 | A_23_P312174_riset1 | 2,94E-13 | 3,15 |
| 239 | NEFL | A_24_P18137_riset1 | 7,32E-09 | 3,15 |
| 240 | DENND2D | A_23_P85952_riset1 | 2,20E-10 | 3,14 |
| 241 | RAI16 | A_23_P157620_riset1 | 4,44E-13 | 3,14 |
| 242 | CCT3 | A_23_P160631_riset1 | 2,86E-10 | 3,14 |
| 243 | NUBPL | A_23_P170906_riset1 | 8,26E-08 | 3,14 |
| 244 | MCPH1 | A_23_P84350_riset1 | 5,57E-06 | 3,14 |
| 245 | DKK3 | A_23_P162047_riset1 | 4,29E-07 | 3,13 |
| 246 | NOP5/NOP58 | A_23_P154447_riset1 | 5,27E-09 | 3,12 |
| 247 | PDE7B | MIL_PPPID399200234_riset1 | 8,68E-04 | 3,12 |
| 248 | AKR1B1 | MIL_PPPID394308032_riset1 | 2,84E-12 | 3,11 |
| 249 | STAMBPL1 | A_23_P385322_riset1 | 3,42E-07 | 3,10 |
| 250 | LTB | A_23_P93348_riset1 | 2,37E-07 | 3,09 |
| 251 | WDR52 | A_24_P14932_riset1 | 9,85E-07 | 3,08 |
| 252 | PRKCZ | A_24_P924462_riset1 | 5,06E-08 | 3,07 |
| 253 | RORC | A_23_P324107_riset1 | 2,05E-09 | 3,05 |
| 254 | NFATC2 | NFATC2_riset2 | 1,66E-08 | 3,04 |
| 255 | ADAM23 | A_23_P351667_riset1 | 1,27E-05 | 3,03 |
| 256 | ZNF331 | A_23_P50376_riset1 | 6,81E-13 | 3,02 |
| 257 | FCRL3 | A_23_P358438_riset1 | 3,59E-05 | 3,02 |
| 258 | RCAN2 | MIL_PPPID394307944_riset1 | 1,70E-05 | 3,01 |
| 259 | MAGEH1 | A_23_P34144_riset1 | 4,76E-08 | 3,01 |
| 260 | BC030106 | A_24_P307963_riset1 | 2,28E-07 | 3,01 |
| 261 | IL29 | IL29_riset2 | 4,25E-07 | 3,00 |
| 262 | AKAP1 | AKAP1_riset2 | 9,94E-07 | 3,00 |
