# Supplementary material for: Sequential Targeting of CD52 and TNF Allows Early Minimization Therapy in Kidney Transplantation: From a Biomarker to Targeting in a Proof-Of-Concept Trial
Source: PLoS One. 2017 Jan 13;12(1):e0169624. doi: 10.1371/journal.pone.0169624 (PMC5234822; doi:10.1371/journal.pone.0169624)
Supplement: S5 Table — Complete list of 179 probes ranked according to median fold change (only fold changes ≥3 were included) with corresponding p values (two-tailed t test) and microarray probe ID. (DOCX) [file pone.0169624.s009.docx]

| Supplemental Table S5. List of genes significantly down-regulated in pre-Tx samples compared to samples at 3 weeks after Tx. Complete list of 179 probes ranked according to median fold change (only fold changes ≥3 were included) with corresponding p values (two-tailed t test) and microarray probe ID. | | | | |
| --- | --- | --- | --- | --- |
| **Rank** | **Gene Name** | **Probe ID** | **p** | **Fold change** |
| 1 | RNF8 | RNF8_riset2 | 5,04E-03 | -13,30 |
| 2 | FERMT2 | A_23_P88339_riset1 | 1,79E-03 | -11,20 |
| 3 | C10ORF109 | A_32_P29954_riset1 | 1,04E-03 | -10,44 |
| 4 | RARA | A_23_P207842_riset1 | 5,89E-03 | -9,16 |
| 5 | TEAD1 | A_23_P362893_riset1 | 9,24E-04 | -8,95 |
| 6 | SCGB1A1 | A_23_P150583_riset1 | 5,51E-03 | -8,75 |
| 7 | TEK | A_23_P374695_riset1 | 1,24E-04 | -8,45 |
| 8 | IFI27 | A_24_P270460_riset2 | 1,08E-02 | -8,12 |
| 9 | NTNG1 | A_23_P201547_riset1 | 1,96E-03 | -7,86 |
| 10 | ACHE | A_24_P60845_riset1 | 3,37E-03 | -7,53 |
| 11 | MUC6 | MUC6_riset2 | 1,28E-02 | -6,82 |
| 12 | CXCL10 | A_24_P303091_riset1 | 4,87E-07 | -6,57 |
| 13 | GGTLA1 | A_24_P297182_riset1 | 5,54E-03 | -6,49 |
| 14 | PPARGC1A | A_23_P18443_riset1 | 2,15E-07 | -6,39 |
| 15 | AIFM2 | A_23_P138567_riset1 | 3,95E-03 | -6,15 |
| 16 | HSP90B1 | A_23_P2601_riset1 | 3,13E-02 | -6,12 |
| 17 | OLR1 | A_24_P124624_riset1 | 1,26E-03 | -5,89 |
| 18 | CAV1 | MIL_PPPID394453438_riset1 | 2,82E-05 | -5,88 |
| 19 | THC2378360 | A_32_P19466_riset1 | 8,11E-04 | -5,85 |
| 20 | KCNK7 | A_23_P86874_riset1 | 2,34E-02 | -5,81 |
| 21 | CLPS | A_23_P8142_riset1 | 1,44E-03 | -5,78 |
| 22 | GPR31 | A_23_P42112_riset1 | 1,82E-03 | -5,75 |
| 23 | MAPK7 | MAPK7_riset2_piqor | 2,17E-03 | -5,68 |
| 24 | C17ORF65 | A_32_P448360_riset1 | 1,19E-03 | -5,58 |
| 25 | IL22RA1 | IL22RA1_riset2 | 3,06E-03 | -5,50 |
| 26 | FOXQ1 | FOXQ1_riset2 | 1,75E-02 | -5,42 |
| 27 | DLD | MIL_PPPID399806226_riset1 | 9,44E-04 | -5,39 |
| 28 | CLEC9A | A_23_P105685_riset1 | 2,86E-08 | -5,26 |
| 29 | CDS1 | A_23_P7245_riset1 | 8,99E-04 | -5,25 |
| 30 | CNTNAP1 | A_23_P411112_riset1 | 3,59E-03 | -5,15 |
| 31 | C14ORF139 | A_23_P14302_riset1 | 2,29E-02 | -5,14 |
| 32 | LOC284440 | A_24_P375168_riset1 | 2,49E-04 | -5,10 |
| 33 | IL8 | A_32_P87013_riset1 | 2,49E-02 | -5,07 |
| 34 | OR4D1 | A_23_P66392_riset1 | 8,27E-03 | -5,05 |
| 35 | CALD1 | A_24_P921366_riset1 | 4,29E-07 | -4,96 |
| 36 | NFATC4 | MIL_PPPID394307964_riset1 | 1,49E-03 | -4,92 |
| 37 | DUSP4 | A_23_P134935_riset1 | 3,64E-03 | -4,86 |
| 38 | CD80 | A_24_P320033_riset1 | 8,65E-04 | -4,86 |
| 39 | RGR | A_24_P103911_riset1 | 1,58E-04 | -4,84 |
| 40 | STC1 | A_23_P314755_riset1 | 9,83E-05 | -4,78 |
| 41 | DPT | A_23_P200741_riset1 | 7,65E-04 | -4,64 |
| 42 | TYMS | A_23_P50096_riset1 | 2,14E-05 | -4,64 |
| 43 | ORAI2 | ORAI2_riset2 | 6,63E-03 | -4,62 |
| 44 | ETV7 | A_23_P42353_riset1 | 1,90E-06 | -4,62 |
| 45 | IL17A | A_23_P332820_riset1 | 1,02E-04 | -4,59 |
| 46 | IL28B | IL28B_riset2 | 5,66E-04 | -4,58 |
| 47 | VSIG10 | A_23_P72059_riset1 | 8,58E-04 | -4,55 |
| 48 | WASF3 | A_24_P176079_riset1 | 4,26E-06 | -4,55 |
| 49 | MUC8 | MUC8_riset2 | 4,20E-04 | -4,54 |
| 50 | GNAO1 | GNAO1_2_riset2 | 1,18E-03 | -4,53 |
| 51 | MAOB | A_23_P85008_riset1 | 1,18E-03 | -4,52 |
| 52 | PCP4 | A_23_P109322_riset1 | 2,75E-03 | -4,48 |
| 53 | CNN1 | A_23_P16239_riset1 | 9,80E-07 | -4,47 |
| 54 | FAS | A_23_P63896_riset1 | 2,51E-11 | -4,46 |
| 55 | SLC22A23 | A_24_P252223_riset1 | 2,81E-03 | -4,44 |
| 56 | IFNA1 | IFNA1_riset2 | 8,89E-04 | -4,44 |
| 57 | EMP1 | A_23_P76488_riset1 | 4,42E-04 | -4,43 |
| 58 | PGM5 | A_24_P120907_riset1 | 3,69E-02 | -4,39 |
| 59 | NAV3 | A_23_P13740_riset1 | 6,17E-03 | -4,37 |
| 60 | IFIT1 | IFIT1_riset2_piqor | 9,61E-05 | -4,36 |
| 61 | CLCA3 | A_23_P34382_riset1 | 1,01E-03 | -4,33 |
| 62 | TRY6 | MIL_PPPID399806180_riset1 | 5,89E-03 | -4,32 |
| 63 | NKX2-5 | A_23_P396135_riset1 | 1,14E-03 | -4,32 |
| 64 | KEL | A_23_P252758_riset1 | 2,00E-05 | -4,32 |
| 65 | HIST2H2BE | A_32_P143496_rev_riset1 | 4,71E-09 | -4,31 |
| 66 | FOXH1 | FOXH1_riset2 | 1,40E-03 | -4,30 |
| 67 | THC2423232 | A_32_P235289_riset1 | 2,18E-03 | -4,29 |
| 68 | TMPRSS2 | A_23_P29067_riset1 | 3,49E-04 | -4,25 |
| 69 | GBP1 | A_23_P62890_riset1 | 2,70E-08 | -4,23 |
| 70 | GGTL4 | MIL_PPPID399200290_riset1 | 2,98E-02 | -4,19 |
| 71 | NGFR | A_23_P389894_riset1 | 1,53E-02 | -4,14 |
| 72 | EGR2 | A_23_P46936_riset1 | 4,80E-03 | -4,13 |
| 73 | COL12A1 | A_23_P214168_riset1 | 1,99E-04 | -4,13 |
| 74 | CD274 | A_23_P338479_riset1 | 1,08E-05 | -4,10 |
| 75 | SNIP | A_24_P248083_riset1 | 4,14E-04 | -4,09 |
| 76 | TJP1 | A_23_P205828_riset1 | 9,08E-06 | -4,07 |
| 77 | ADRB3 | A_23_P168993_riset1 | 1,10E-03 | -4,05 |
| 78 | SPTA1 | A_23_P63158_riset1 | 3,71E-05 | -4,04 |
| 79 | TNFRSF10B | TNFRSF10B_riset2 | 3,88E-04 | -4,03 |
| 80 | SOCS1 | A_23_P420196_riset1 | 6,43E-06 | -4,01 |
| 81 | FAM148B | MIL_PPPID399200342_riset1 | 1,08E-03 | -3,99 |
| 82 | IFNA13 | IFNA13_riset2 | 2,09E-04 | -3,94 |
| 83 | LTBP1 | A_23_P43810_riset1 | 1,78E-05 | -3,93 |
| 84 | SH3BGRL2 | A_24_P209171_riset1 | 2,18E-05 | -3,92 |
| 85 | NOV | A_23_P82929_riset1 | 4,48E-07 | -3,92 |
| 86 | INHBA | A_23_P122924_riset1 | 6,09E-03 | -3,92 |
| 87 | ELA2B | A_23_P51711_riset1 | 1,39E-05 | -3,88 |
| 88 | CYP4F2 | MIL_PPPID399806210_riset1 | 3,14E-02 | -3,86 |
| 89 | GPX2 | A_23_P3038_riset1 | 4,94E-04 | -3,86 |
| 90 | VIL1 | A_23_P16866_riset1 | 1,33E-05 | -3,85 |
| 91 | C1S | A_23_P2492_riset1 | 5,05E-03 | -3,83 |
| 92 | PDLIM4 | A_23_P144796_riset1 | 4,00E-04 | -3,82 |
| 93 | PDE5A | MIL_PPPID399200142_riset1 | 1,27E-06 | -3,81 |
| 94 | ELOVL7 | A_23_P359277_riset1 | 8,07E-08 | -3,80 |
| 95 | LMX1A | A_23_P34690_riset1 | 2,28E-05 | -3,76 |
| 96 | F10 | A_23_P205172_riset1 | 5,68E-04 | -3,76 |
| 97 | LOC282997 | A_24_P153642_riset1 | 2,46E-06 | -3,75 |
| 98 | ACADL | A_23_P28097_riset1 | 6,37E-04 | -3,75 |
| 99 | APOE | A_23_P164650_riset1 | 1,29E-03 | -3,74 |
| 100 | COL20A1 | A_32_P185644_riset1 | 2,40E-03 | -3,74 |
| 101 | SYTL4 | A_24_P122337_riset1 | 3,78E-04 | -3,70 |
| 102 | OR8J1 | A_23_P13244_riset1 | 9,32E-04 | -3,69 |
| 103 | GGNBP1 | A_24_P606538_riset1 | 7,62E-04 | -3,66 |
| 104 | WFDC2 | A_23_P218675_riset1 | 4,68E-09 | -3,65 |
| 105 | INHA | A_23_P51036_riset1 | 3,34E-04 | -3,63 |
| 106 | FSTL1 | A_23_P212696_riset1 | 2,52E-06 | -3,62 |
| 107 | ZNF516 | A_24_P39894_riset1 | 1,35E-06 | -3,56 |
| 108 | SPRED2 | A_32_P225846_riset1 | 4,76E-03 | -3,56 |
| 109 | PSMD8 | MIL_PPPID402610908_riset2 | 1,68E-02 | -3,54 |
| 110 | SFRP1 | A_23_P10127_riset1 | 2,34E-05 | -3,54 |
| 111 | TGFB1I1 | A_23_P141055_riset1 | 2,13E-05 | -3,50 |
| 112 | LOC100130506;LOC100288911 | A_24_P486427_riset1 | 5,21E-04 | -3,50 |
| 113 | LAMA3 | LAMA3_riset2 | 1,49E-03 | -3,49 |
| 114 | C10ORF10 | A_24_P329795_riset1 | 2,20E-03 | -3,49 |
| 115 | TNS1 | A_24_P105733_riset1 | 7,74E-05 | -3,46 |
| 116 | TGM2 | A_32_P86763_riset1 | 8,99E-05 | -3,46 |
| 117 | OR5AP2 | A_23_P47464_riset1 | 3,24E-03 | -3,46 |
| 118 | BMP6 | BMP6_riset2 | 4,81E-10 | -3,45 |
| 119 | LHFP | A_23_P88069_riset1 | 1,05E-07 | -3,44 |
| 120 | MAK | A_24_P363278_riset1 | 1,78E-07 | -3,44 |
| 121 | ITGB5 | MIL_PPPID394307852_riset1 | 2,14E-04 | -3,43 |
| 122 | PDE1A | A_24_P208436_riset1 | 1,27E-03 | -3,43 |
| 123 | UPK3B | A_23_P328110_riset1 | 1,52E-05 | -3,43 |
| 124 | CACNA1A | A_24_P130559_riset1 | 6,52E-05 | -3,43 |
| 125 | KRT76 | A_23_P61498_riset1 | 1,98E-04 | -3,43 |
| 126 | IGF2 | MIL_PPPID397416219_riset1 | 4,67E-03 | -3,42 |
| 127 | CDC45L | A_23_P57379_riset1 | 2,29E-05 | -3,41 |
| 128 | ELA2A | A_23_P46238_riset1 | 5,62E-05 | -3,41 |
| 129 | BC037919 | A_32_P47538_riset1 | 9,33E-03 | -3,40 |
| 130 | FADS2 | A_23_P98580_riset1 | 7,47E-03 | -3,39 |
| 131 | IGF2;INS-IGF2 | A_23_P150609_riset1 | 4,46E-03 | -3,38 |
| 132 | COL17A1 | A_23_P501010_riset1 | 2,35E-03 | -3,38 |
| 133 | SLCO2A1 | A_32_P178549_riset1 | 1,68E-04 | -3,37 |
| 134 | GH1 | MIL_PPPID399806221_riset1 | 1,58E-03 | -3,36 |
| 135 | IFIT1 | A_23_P52266_riset1 | 5,79E-04 | -3,35 |
| 136 | LOC100128616 | A_32_P47870_riset1 | 6,55E-05 | -3,35 |
| 137 | SLC17A7 | A_23_P78750_riset1 | 1,47E-03 | -3,34 |
| 138 | UPB1 | A_23_P120822_riset1 | 3,01E-05 | -3,32 |
| 139 | KIFC3 | A_23_P54576_riset1 | 3,13E-04 | -3,31 |
| 140 | TUBB1 | A_24_P228550_riset1 | 4,67E-06 | -3,31 |
| 141 | TFPI | MIL_PPPID397416239_riset1 | 1,56E-07 | -3,27 |
| 142 | SERPING1 | A_23_P139114_riset1 | 1,99E-03 | -3,26 |
| 143 | CD177 | CD177_riset2 | 5,75E-03 | -3,26 |
| 144 | RTP1 | A_32_P528967_riset1 | 6,21E-04 | -3,25 |
| 145 | DEFA4 | A_23_P326080_riset1 | 1,30E-02 | -3,24 |
| 146 | CYP1A2 | CYP1A2_riset2 | 9,96E-05 | -3,23 |
| 147 | CCL21 | A_23_P112470_riset1 | 5,53E-04 | -3,23 |
| 148 | PTGER3 | MIL_PPPID397416191_riset1 | 6,63E-06 | -3,23 |
| 149 | A_24_P940041 | A_24_P940041_riset1 | 1,29E-03 | -3,22 |
| 150 | KLKB1 | A_23_P252236_riset1 | 6,57E-03 | -3,21 |
| 151 | RHCE;RHD | MIL_PPPID399806229_riset1 | 8,48E-04 | -3,21 |
| 152 | MPL | A_24_P156769_riset1 | 3,09E-05 | -3,21 |
| 153 | FOXO4 | FOXO4_riset2 | 9,21E-06 | -3,20 |
| 154 | DDEF2 | A_24_P362540_riset1 | 1,53E-08 | -3,19 |
| 155 | SDC2 | MIL_PPPID394453397_riset1 | 9,23E-04 | -3,19 |
| 156 | CKAP2L | A_32_P208011_riset1 | 4,89E-05 | -3,18 |
| 157 | ERG | A_23_P57323_riset1 | 5,44E-03 | -3,18 |
| 158 | RRM2 | A_24_P225616_riset1 | 2,84E-05 | -3,17 |
| 159 | ZNF385D | A_23_P113748_riset1 | 2,93E-03 | -3,17 |
| 160 | BG944303 | A_24_P937169_riset1 | 3,88E-04 | -3,17 |
| 161 | CD276 | CD276_riset2 | 3,41E-03 | -3,15 |
| 162 | KCNS1 | A_23_P321846_riset1 | 7,10E-04 | -3,15 |
| 163 | TSPAN7 | A_23_P114185_riset1 | 3,42E-03 | -3,14 |
| 164 | NOS1 | A_23_P204791_riset1 | 1,65E-03 | -3,14 |
| 165 | HRG | A_23_P321892_riset1 | 1,03E-02 | -3,14 |
| 166 | PDE6H | A_23_P64913_riset1 | 1,58E-03 | -3,10 |
| 167 | TACSTD2 | A_23_P149529_riset1 | 1,51E-02 | -3,09 |
| 168 | PLK3 | A_23_P51646_riset1 | 4,84E-03 | -3,09 |
| 169 | CDC42BPA | A_23_P256190_riset1 | 8,81E-05 | -3,08 |
| 170 | BAZ2B | A_23_P120048_riset1 | 1,61E-08 | -3,07 |
| 171 | EGF | A_23_P155979_riset1 | 6,02E-07 | -3,06 |
| 172 | LAMP3 | A_23_P29773_riset1 | 1,76E-03 | -3,06 |
| 173 | DHRS9 | A_23_P56559_riset1 | 3,35E-08 | -3,05 |
| 174 | PTPRF | A_32_P416233_riset1 | 3,12E-04 | -3,05 |
| 175 | MYL4 | A_24_P188218_riset1 | 1,34E-02 | -3,04 |
| 176 | ITGB3 | ITGB3_riset2 | 1,39E-04 | -3,04 |
| 177 | CRP | A_23_P339042_riset1 | 1,89E-04 | -3,00 |
| 178 | IFIT2 | A_23_P24004_riset1 | 1,84E-08 | -3,00 |
| 179 | ELA2 | A_23_P130961_riset1 | 1,53E-02 | -3,00 |
