# Supplementary material for: Sequential Targeting of CD52 and TNF Allows Early Minimization Therapy in Kidney Transplantation: From a Biomarker to Targeting in a Proof-Of-Concept Trial
Source: PLoS One. 2017 Jan 13;12(1):e0169624. doi: 10.1371/journal.pone.0169624 (PMC5234822; doi:10.1371/journal.pone.0169624)
Supplement: S6 Table — Only the first 15 annotations with the highest statistical significance are shown. (DOCX) [file pone.0169624.s010.docx]

Supplemental Table S6. Annotation enrichment of genes with significantly higher differential expression in pre-TX samples compared with samples collected three weeks after Tx. Only the first 15 annotations with the highest statistical significance are shown.

| **Annotation** | **transcriptional cell marker: T cells (pan) (Chtanova et al. J. Immunol. 2005)** | **transcriptional cell marker: memory T cells (Chtanova et al. J. Immunol. 2005)** | **transcriptional cell marker: T cells (pan) (Chtanova et al. J. Immunol. 2005)** | **Tcell receptor signaling** | **Downregulated by IL2 Pathway** | **Generation of second messenger molecules** | **B cell surface marker** | **T cell surface marker** | **B Cell Receptor Signaling** | **lymphocyte differentiation** | **transcriptional cell markers: B cells (Chtanova et al. J. Immunol. 2005)** | **external side of plasma membrane** | **role of mef2d in t-cell apoptosis** | **immune response-regulating cell surface receptor signaling pathway** | **Ribosome** |
| --- | --- | --- | --- | --- | --- | --- | --- | --- | --- | --- | --- | --- | --- | --- | --- |
| **Significance** | 2.97E-13 | 7.78E-12 | 3.58E-10 | 1.12E-07 | 6.43E-07 | 1.00E-06 | 1.90E-06 | 3.80E-05 | 4.30E-05 | 1.07E-04 | 1.74E-04 | 2.36E-04 | 3.42E-04 | 3.90E-04 | 4.00E-04 |
| **Enrichment** | 6.22 | 13.81 | 16.32 | 6.01 | 3.44 | 7.1 | 4.45 | 3.41 | 6.8 | 3.33 | 5.71 | 3.01 | 6.22 | 4.49 | 13.6 |
| **Count** | 24 | 11 | 8 | 31 | 24 | 10 | 15 | 14 | 19 | 21 | 7 | 21 | 8 | 11 | 12 |
| **Name** |  |  |  |  |  |  |  |  |  |  |  |  |  |  |  |
| ABLIM1 |  |  |  |  | **X** |  |  |  |  |  |  |  |  |  |  |
| AQP3 |  |  |  |  | **X** |  |  |  |  |  |  |  |  |  |  |
| BCKDHB |  |  |  |  | **X** |  |  |  |  |  |  |  |  |  |  |
| BCL2 |  |  |  |  |  |  |  |  | **X** | **X** |  |  |  |  |  |
| BLK |  |  |  |  |  |  |  |  | **X** |  | **X** |  |  |  |  |
| BLNK |  |  |  |  |  |  |  |  | **X** | **X** | **X** |  |  |  |  |
| BTLA |  |  |  |  |  |  |  |  |  |  |  | **X** |  | **X** |  |
| CARD11 |  |  |  | **X** |  |  |  |  | **X** | **X** |  |  |  |  |  |
| CCR5 |  |  |  |  |  |  |  |  |  |  |  | **X** |  |  |  |
| CCR7 |  |  |  |  | **X** |  |  |  |  |  |  |  |  |  |  |
| CCT3 |  |  |  |  |  |  |  |  |  |  |  |  |  |  | **X** |
| CD19 |  |  |  |  |  |  | **X** |  | **X** |  | **X** | **X** |  | **X** |  |
| CD2 |  |  |  | **X** | **X** |  | **X** | **X** |  | **X** |  | **X** |  |  |  |
| CD22 |  |  |  |  |  |  | **X** |  | **X** |  |  | **X** |  |  |  |
| CD28 | **X** |  | **X** | **X** |  |  | **X** | **X** |  | **X** |  | **X** |  |  |  |
| CD3D | **X** |  | **X** | **X** |  | **X** |  |  |  | **X** |  |  | **X** |  |  |
| CD3G | **X** |  | **X** | **X** |  | **X** |  |  |  |  |  |  | **X** |  |  |
| CD40LG | **X** |  | **X** | **X** |  |  |  |  |  | **X** |  |  |  |  |  |
| CD5 |  |  |  | **X** | **X** |  | **X** | **X** | **X** |  |  | **X** |  |  |  |
| CD52 |  |  |  |  | **X** |  |  | **X** |  |  |  |  |  |  |  |
| CD6 |  |  |  |  | **X** |  | **X** | **X** |  |  |  | **X** |  |  |  |
| CD70 | **X** |  |  |  | **X** |  | **X** | **X** |  |  |  |  |  |  |  |
| CD79A |  |  |  |  |  |  | **X** | **X** | **X** | **X** |  | **X** |  | **X** |  |
| CD79B |  |  |  |  |  |  | **X** |  | **X** |  | **X** | **X** |  | **X** |  |
| CD8A |  |  |  | **X** |  |  |  |  |  | **X** |  | **X** |  |  |  |
| CD96 | **X** | **X** |  |  |  |  | **X** | **X** |  |  |  |  |  |  |  |
| CLCF1 |  |  |  |  |  |  |  |  |  | **X** |  |  |  |  |  |
| CTLA4 | **X** |  |  | **X** |  |  | **X** | **X** |  | **X** |  | **X** |  |  |  |
| CXCR3 |  |  |  |  | **X** |  |  | **X** |  |  |  |  |  |  |  |
| DENND2D |  |  |  |  | **X** |  |  |  |  |  |  |  |  |  |  |
| DPP4 | **X** |  | **X** |  | **X** |  | **X** | **X** |  |  |  |  |  |  |  |
| DTX1 |  |  |  | **X** |  |  |  |  |  |  |  |  |  |  |  |
| EBI2 |  |  |  |  | **X** |  |  |  |  | **X** |  |  |  |  |  |
| EDG1 |  |  |  |  |  |  |  |  |  |  |  | **X** |  |  |  |
| EEF1A1;EEF1AL7 |  |  |  |  |  |  |  |  |  |  |  |  |  |  | **X** |
| FCER2 |  |  |  |  |  |  | **X** | **X** |  |  |  | **X** |  |  |  |
| FCGBP |  |  |  |  | **X** |  |  |  |  |  |  |  |  |  |  |
| FCRL2 |  |  |  |  |  |  |  |  |  |  | **X** |  |  |  |  |
| FCRL3 |  |  |  | **X** |  |  |  |  |  |  |  |  |  |  |  |
| FLT3LG | **X** | **X** |  |  |  |  |  |  |  | **X** |  | **X** |  |  |  |
| FYN |  |  |  | **X** |  |  |  |  | **X** |  |  |  | **X** | **X** |  |
| GZMK | **X** | **X** |  |  | **X** |  |  |  |  |  |  |  |  |  |  |
| RPSA |  |  |  |  |  |  |  |  |  |  |  |  |  |  | **X** |
| HLA-DOA |  |  |  | **X** |  | **X** |  |  |  | **X** |  |  |  |  |  |
| HLA-DQA2 |  |  |  | **X** |  | **X** |  |  |  |  |  |  |  |  |  |
| HLTF |  |  |  |  | **X** |  |  |  |  |  |  |  |  |  |  |
| ICOS | **X** |  | **X** | **X** |  |  |  |  |  |  |  | **X** |  |  |  |
| IGFBP3 | **X** | **X** |  |  |  |  |  |  |  |  |  |  |  |  |  |
| IL2RA | **X** |  |  | **X** |  |  | **X** | **X** |  | **X** |  | **X** |  |  |  |
| IL7R | **X** | **X** |  |  | **X** |  |  |  |  | **X** |  | **X** |  |  |  |
| ITGB7 |  |  |  |  | **X** |  |  |  |  |  |  |  |  |  |  |
| ITK |  |  |  | **X** |  | **X** |  |  | **X** |  |  |  |  |  |  |
| LAG3 | **X** |  |  |  |  |  |  |  |  |  |  | **X** |  |  |  |
| LAT |  |  |  | **X** | **X** | **X** |  |  |  |  |  |  | **X** |  |  |
| LAX1 |  |  |  | **X** |  |  |  |  |  |  |  |  |  | **X** |  |
| LCK |  |  |  | **X** |  | **X** |  |  | **X** | **X** |  |  | **X** | **X** |  |
| LEF1 | **X** | **X** |  |  |  |  |  |  |  |  |  |  |  |  |  |
| LY9 |  |  |  |  | **X** |  |  |  |  |  |  |  |  |  |  |
| MAL | **X** |  | **X** |  |  |  |  |  |  |  |  |  |  |  |  |
| MS4A1 |  |  |  |  |  |  | **X** | **X** |  |  | **X** | **X** |  |  |  |
| NEFL | **X** | **X** |  |  |  |  |  |  |  |  |  |  |  |  | **X** |
| NELL2 | **X** | **X** |  |  | **X** |  |  |  |  |  |  |  |  |  |  |
| NFATC2 |  |  |  | **X** |  |  |  |  | **X** |  |  |  |  |  |  |
| NR4A1 |  |  |  | **X** |  |  |  |  |  |  |  |  | **X** |  |  |
| NY-REN-7 | **X** | **X** |  |  |  |  |  |  |  |  |  |  |  |  |  |
| PLCG1 |  |  |  | **X** |  | **X** |  |  | **X** |  |  |  | **X** | **X** |  |
| PLEKHA1 |  |  |  |  |  |  |  |  | **X** |  |  |  |  | **X** |  |
| PRKCQ |  |  |  | **X** |  |  |  |  | **X** |  |  |  |  |  |  |
| PTCH1 |  |  |  |  | **X** |  |  |  |  |  |  |  |  |  |  |
| RASGRP1 |  |  |  | **X** |  |  |  |  |  |  |  |  |  |  |  |
| RCAN2 |  |  |  | **X** |  |  |  |  |  |  |  |  |  |  |  |
| RHOH |  |  |  |  |  |  |  |  |  | **X** |  |  |  |  |  |
| RORC |  |  |  |  |  |  |  |  |  | **X** |  |  |  |  |  |
| RPL14 |  |  |  |  |  |  |  |  |  |  |  |  |  |  | **X** |
| RPL23 |  |  |  |  |  |  |  |  |  |  |  |  |  |  | **X** |
| RPL35 |  |  |  |  |  |  |  |  |  |  |  |  |  |  | **X** |
| RPL9 |  |  |  |  |  |  |  |  |  |  |  |  |  |  | **X** |
| RPS21 |  |  |  |  |  |  |  |  |  |  |  |  |  |  | **X** |
| RPS27 |  |  |  |  |  |  |  |  |  |  |  |  |  |  | **X** |
| RPS27A |  |  |  | **X** |  |  |  |  |  |  |  |  |  |  | **X** |
| RPS6 |  |  |  |  |  |  |  |  | **X** | **X** |  |  |  |  | **X** |
| SLAMF1 |  |  |  |  |  |  |  |  |  |  |  | **X** |  |  |  |
| STAT4 |  |  |  | **X** |  |  |  |  |  |  |  |  |  |  |  |
| TCF7 | **X** | **X** |  |  | **X** |  |  |  |  |  |  |  |  |  |  |
| TCL1A |  |  |  |  |  |  |  |  | **X** |  | **X** |  |  |  |  |
| TNFRSF25 | **X** | **X** |  |  | **X** |  |  |  |  |  |  |  |  |  |  |
| TRA@ | **X** |  | **X** | **X** |  | **X** |  |  |  |  |  |  |  |  |  |
| TRAT1 |  |  |  | **X** |  |  |  |  |  |  |  |  |  | **X** |  |
| ZAP70 |  |  |  | **X** |  | **X** |  |  | **X** | **X** |  |  | **X** | **X** |  |
| ZBTB32 | **X** |  |  |  |  |  |  |  |  |  |  |  |  |  |  |
