# Supplementary material for: Sequential Targeting of CD52 and TNF Allows Early Minimization Therapy in Kidney Transplantation: From a Biomarker to Targeting in a Proof-Of-Concept Trial
Source: PLoS One. 2017 Jan 13;12(1):e0169624. doi: 10.1371/journal.pone.0169624 (PMC5234822; doi:10.1371/journal.pone.0169624)
Supplement: S7 Table — Complete list of 93 probes ranked according to median fold change (only fold changes ≥1.5 were included) with corresponding p values (two-tailed t test) and microarray probe ID. (DOCX) [file pone.0169624.s011.docx]

| Supplemental Table S7. List of genes significantly up-regulated in W3 samples of patients from Tacrolimus group compared to patients from Sirolimus group . Complete list of 93 probes ranked according to median fold change (only fold changes ≥1.5 were included) with corresponding p values (two-tailed t test) and microarray probe ID. | | | | |
| --- | --- | --- | --- | --- |
| **Rank** | **Gene Name** | **Probe ID** | **p** | **Fold change** |
| 1 | RAP1GAP | A_24_P36890_riset1 | 4,74E-02 | 12,751 |
| 2 | LOC284440 | A_24_P375168_riset1 | 2,36E-02 | 5,713 |
| 3 | TCL1A | MIL_PPPID394307780_riset1 | 3,65E-02 | 5,093 |
| 4 | TCL1A | A_23_P357717_riset1 | 6,49E-03 | 4,468 |
| 5 | IGF2;INS-IGF2 | A_23_P150609_riset1 | 2,73E-02 | 4,431 |
| 6 | IGF2 | MIL_PPPID397416219_riset1 | 4,68E-02 | 3,763 |
| 7 | CRYM | A_23_P77731_riset1 | 4,65E-03 | 3,620 |
| 8 | BRD4 | MIL_PPPID399806183_riset1 | 4,11E-02 | 3,502 |
| 9 | GRB14 | A_23_P154526_riset1 | 1,85E-02 | 3,475 |
| 10 | SERPINE1 | A_24_P158089_riset1 | 3,76E-02 | 3,246 |
| 11 | DOC2B;LOC653051 | A_24_P236753_riset1 | 3,59E-03 | 3,241 |
| 12 | HECW1 | A_32_P102474_riset1 | 3,34E-03 | 3,013 |
| 13 | NGFRAP1 | A_23_P45524_riset1 | 5,32E-03 | 2,955 |
| 14 | MYLK | A_24_P319923_riset1 | 1,92E-02 | 2,861 |
| 15 | KLHDC7B | A_23_P6535_riset1 | 2,26E-03 | 2,852 |
| 16 | GM2A | A_23_P144866_riset1 | 3,19E-04 | 2,816 |
| 17 | RAB30 | A_23_P139359_riset1 | 3,39E-02 | 2,798 |
| 18 | LAG3 | A_23_P116942_riset1 | 4,70E-02 | 2,784 |
| 19 | AKAP13 | A_24_P636130_riset1 | 8,70E-03 | 2,744 |
| 20 | SOX3 | A_24_P113725_riset1 | 4,43E-03 | 2,696 |
| 21 | A_24_P936172 | A_24_P936172_riset1 | 9,34E-03 | 2,609 |
| 22 | PRSS3 | A_23_P135257_riset1 | 4,59E-02 | 2,602 |
| 23 | RNASE1 | A_24_P403959_riset1 | 2,80E-03 | 2,571 |
| 24 | RP11-167P23.2 | A_24_P315014_riset1 | 1,42E-02 | 2,549 |
| 25 | C1QC | A_23_P125974_riset1 | 2,53E-02 | 2,513 |
| 26 | SEMG2 | A_23_P5968_riset1 | 2,59E-02 | 2,456 |
| 27 | HPCAL4 | A_24_P139665_riset1 | 1,93E-02 | 2,435 |
| 28 | PROS1 | A_23_P73114_riset1 | 1,96E-02 | 2,421 |
| 29 | RNF39 | A_24_P314931_riset1 | 8,68E-03 | 2,415 |
| 30 | TSPAN7 | A_23_P114185_riset1 | 4,55E-02 | 2,406 |
| 31 | PDE2A | A_23_P401106_riset1 | 7,75E-04 | 2,406 |
| 32 | PLXNB1 | A_23_P57961_riset1 | 3,24E-02 | 2,395 |
| 33 | FCER1G | A_23_P160849_riset1 | 1,36E-02 | 2,305 |
| 34 | BLK | BLK_riset2 | 3,29E-02 | 2,289 |
| 35 | ACOT7 | A_24_P205589_riset1 | 3,64E-02 | 2,251 |
| 36 | ALDH1L1 | A_24_P941870_riset1 | 2,10E-02 | 2,221 |
| 37 | CCL5 | A_23_P152837_riset1 | 3,28E-03 | 2,204 |
| 38 | STC2 | A_23_P110686_riset1 | 4,00E-03 | 2,184 |
| 39 | TNFRSF9 | A_23_P51936_riset1 | 2,30E-02 | 2,181 |
| 40 | PPP1R14C | A_23_P45011_riset1 | 2,96E-03 | 2,171 |
| 41 | C4ORF31 | A_23_P110266_riset1 | 4,16E-02 | 2,160 |
| 42 | IL21 | A_23_P167250_riset1 | 9,16E-03 | 2,150 |
| 43 | ELA2A | A_23_P46238_riset1 | 1,36E-02 | 2,145 |
| 44 | PTPRM | A_32_P204500_riset1 | 3,76E-02 | 2,074 |
| 45 | TSPAN1 | A_23_P160167_riset1 | 4,40E-02 | 2,028 |
| 46 | ACSBG1 | A_23_P54488_riset1 | 1,66E-02 | 2,005 |
| 47 | SYNGR3 | A_23_P88909_riset1 | 1,91E-02 | 1,973 |
| 48 | LOC619208 | A_32_P234495_riset1 | 4,60E-02 | 1,972 |
| 49 | HERC5 | HERC5_riset2 | 4,13E-02 | 1,959 |
| 50 | DHFR;DHFRP1 | A_24_P343095_riset1 | 4,36E-02 | 1,958 |
| 51 | IL31RA | IL31RA_riset2 | 3,05E-02 | 1,951 |
| 52 | PROZ | A_23_P140074_riset1 | 4,71E-02 | 1,950 |
| 53 | HGD | A_23_P250164_riset1 | 2,45E-02 | 1,944 |
| 54 | MTHFD1L | A_23_P214907_riset1 | 1,16E-02 | 1,941 |
| 55 | REG4 | A_23_P400310_riset1 | 7,34E-03 | 1,918 |
| 56 | RASL11B | A_23_P69738_riset1 | 2,77E-02 | 1,882 |
| 57 | FOLR1 | A_23_P53176_riset1 | 4,05E-02 | 1,877 |
| 58 | BC039524 | A_24_P839298_riset1 | 7,99E-04 | 1,877 |
| 59 | COL28A1 | MIL_PPPID399200250_riset1 | 6,20E-04 | 1,863 |
| 60 | INF2 | A_23_P218131_riset1 | 3,95E-02 | 1,860 |
| 61 | C1QA | C1QA_riset2_piqor | 3,21E-02 | 1,852 |
| 62 | SLC12A3 | A_24_P309912_riset1 | 1,31E-02 | 1,839 |
| 63 | CXCL13 | CXCL13_riset2 | 4,96E-03 | 1,832 |
| 64 | PROS1 | A_23_P84510_riset1 | 2,99E-02 | 1,824 |
| 65 | PVR | A_23_P141894_riset1 | 4,21E-02 | 1,823 |
| 66 | FXYD2 | A_23_P161769_riset1 | 3,71E-02 | 1,815 |
| 67 | SFTPA1B | MIL_PPPID399806186_riset1 | 4,86E-02 | 1,797 |
| 68 | A_24_P940354 | A_24_P940354_riset1 | 1,88E-02 | 1,796 |
| 69 | CDKN2A | A_23_P43484_riset1 | 4,45E-02 | 1,794 |
| 70 | CD3E | CD3E_riset2 | 3,70E-02 | 1,791 |
| 71 | DAP | A_23_P92687_riset1 | 4,99E-02 | 1,775 |
| 72 | AK025173 | A_24_P554331_riset1 | 1,28E-02 | 1,768 |
| 73 | LOC222159 | A_24_P935310_riset1 | 2,44E-02 | 1,756 |
| 74 | C5AR1 | A_23_P303058_riset1 | 4,53E-02 | 1,743 |
| 75 | TAF5L | A_23_P35309_riset1 | 4,48E-02 | 1,732 |
| 76 | C11ORF49 | A_23_P147605_riset1 | 3,42E-02 | 1,702 |
| 77 | IFNK | IFNK_riset2 | 4,09E-03 | 1,697 |
| 78 | SDC4 | A_23_P109034_riset1 | 4,20E-02 | 1,686 |
| 79 | PRO2964 | A_23_P430156_riset1 | 1,83E-02 | 1,674 |
| 80 | STOX1 | A_32_P51119_riset1 | 4,42E-02 | 1,664 |
| 81 | PARD6A | PARD6A_riset2 | 4,22E-02 | 1,641 |
| 82 | LOC339524 | A_32_P867953_riset1 | 2,04E-02 | 1,637 |
| 83 | LOC339524 | A_32_P112592_riset1 | 2,47E-02 | 1,619 |
| 84 | ZNF542 | A_24_P570583_riset1 | 4,35E-03 | 1,600 |
| 85 | IFI6 | A_23_P201459_riset1 | 1,11E-02 | 1,595 |
| 86 | IFI27L1 | A_23_P53976_riset1 | 3,72E-02 | 1,568 |
| 87 | DEFB1 | MIL_PPPID394307940_riset1 | 2,17E-02 | 1,562 |
| 88 | LOC100134674;MUC3B;LOC100134216;MUC3A | MIL_PPPID399200366_riset1 | 4,83E-03 | 1,546 |
| 89 | PFKM | A_24_P98914_riset1 | 3,83E-02 | 1,543 |
| 90 | ABI3 | A_23_P141429_riset1 | 1,22E-02 | 1,532 |
| 91 | ADAMDEC1 | A_23_P256425_riset1 | 2,21E-02 | 1,526 |
| 92 | HHIPL1 | A_24_P162254_riset1 | 4,91E-02 | 1,508 |
| 93 | STC2 | A_23_P416395_riset1 | 3,25E-02 | 1,505 |
