# Supplementary material for: Sequential Targeting of CD52 and TNF Allows Early Minimization Therapy in Kidney Transplantation: From a Biomarker to Targeting in a Proof-Of-Concept Trial
Source: PLoS One. 2017 Jan 13;12(1):e0169624. doi: 10.1371/journal.pone.0169624 (PMC5234822; doi:10.1371/journal.pone.0169624)
Supplement: S8 Table — Complete list of 151 probes ranked according to median fold change (only fold changes ≥1.5 were included) with corresponding p values (two-tailed t test) and microarray probe ID. (DOCX) [file pone.0169624.s012.docx]

| Supplemental Table S8. List of genes significantly down-regulated in W3 samples of patients from Tacrolimus group compared to patients from Sirolimus group . Complete list of 151 probes ranked according to median fold change (only fold changes ≥1.5 were included) with corresponding p values (two-tailed t test) and microarray probe ID.   \| **Rank** \| **Gene Name** \| **Probe ID** \| **p** \| **Fold change** \| \| --- \| --- \| --- \| --- \| --- \| \| 1 \| EMR4 \| MIL_PPPID399200170_riset1 \| 1,12E-02 \| -13,636 \| \| 2 \| IL8 \| A_32_P87013_riset1 \| 1,91E-03 \| -7,729 \| \| 3 \| MMP8 \| A_23_P24493_riset1 \| 2,22E-02 \| -7,389 \| \| 4 \| OLR1 \| A_24_P124624_riset1 \| 4,66E-03 \| -6,158 \| \| 5 \| ADORA3 \| A_23_P137931_riset1 \| 3,94E-03 \| -5,084 \| \| 6 \| ADAMTS7;LOC388194;LOC390660;LOC400406;LOC400419;LOC441730;LOC642935 \| A_32_P786651_riset1 \| 1,75E-02 \| -4,927 \| \| 7 \| CCL23 \| A_24_P133905_riset1 \| 3,89E-03 \| -4,921 \| \| 8 \| CYORF14 \| A_24_P216625_riset1 \| 6,42E-03 \| -3,701 \| \| 9 \| NDUFA5 \| A_23_P412041_riset1 \| 2,55E-02 \| -3,692 \| \| 10 \| SAG \| A_23_P5853_riset1 \| 2,80E-02 \| -3,657 \| \| 11 \| CAND1 \| A_24_P93656_riset1 \| 1,39E-02 \| -3,594 \| \| 12 \| IER3 \| A_23_P42257_riset1 \| 2,59E-02 \| -3,432 \| \| 13 \| SGOL2 \| A_23_P411335_riset1 \| 1,23E-03 \| -3,297 \| \| 14 \| SPP1 \| A_23_P7313_riset1 \| 8,50E-03 \| -3,236 \| \| 15 \| CLCF1 \| A_23_P138760_riset1 \| 2,38E-03 \| -3,227 \| \| 16 \| CLC \| A_23_P101683_riset1 \| 1,02E-03 \| -3,156 \| \| 17 \| IL12RB2 \| IL12RB2_riset2 \| 3,58E-03 \| -3,100 \| \| 18 \| KIT \| A_23_P110253_riset1 \| 2,29E-02 \| -3,077 \| \| 19 \| ATF6 \| A_24_P910360_riset1 \| 6,39E-03 \| -3,077 \| \| 20 \| A_32_P36481 \| A_32_P36481_riset1 \| 2,18E-02 \| -3,041 \| \| 21 \| ATP8B4 \| A_23_P163216_riset1 \| 7,36E-03 \| -2,983 \| \| 22 \| VMO1 \| A_23_P55356_riset1 \| 1,02E-02 \| -2,972 \| \| 23 \| PLCG1 \| MIL_PPPID397416315_riset1 \| 2,06E-02 \| -2,938 \| \| 24 \| GLT25D2 \| A_24_P62505_riset1 \| 1,33E-02 \| -2,903 \| \| 25 \| STYX \| MIL_PPPID397416215_riset1 \| 4,43E-03 \| -2,879 \| \| 26 \| ARV1 \| A_24_P144527_riset2 \| 4,41E-02 \| -2,877 \| \| 27 \| PPAT \| A_23_P80940_riset1 \| 1,95E-03 \| -2,839 \| \| 28 \| DDX43 \| A_23_P156445_riset1 \| 3,70E-03 \| -2,790 \| \| 29 \| IL5RA \| A_23_P500676_riset1 \| 3,46E-03 \| -2,777 \| \| 30 \| THC2317111 \| A_32_P82218_riset1 \| 9,41E-06 \| -2,630 \| \| 31 \| THC2374442 \| A_32_P56037_riset1 \| 1,75E-02 \| -2,597 \| \| 32 \| CMTM4 \| A_23_P391980_riset1 \| 7,26E-03 \| -2,586 \| \| 33 \| SASS6 \| A_24_P349151_riset1 \| 2,09E-02 \| -2,568 \| \| 34 \| UTY \| A_23_P329835_riset1 \| 1,99E-02 \| -2,505 \| \| 35 \| ZBTB44 \| A_23_P385826_riset1 \| 8,77E-03 \| -2,491 \| \| 36 \| GOLGA4 \| A_32_P170895_riset1 \| 2,52E-03 \| -2,377 \| \| 37 \| LOC642236 \| A_32_P201279_riset1 \| 6,13E-03 \| -2,367 \| \| 38 \| LPL \| A_23_P146233_riset1 \| 1,02E-02 \| -2,343 \| \| 39 \| LOXL1 \| A_23_P124084_riset1 \| 3,02E-02 \| -2,334 \| \| 40 \| CCT5 \| A_24_P328872_riset1 \| 6,13E-03 \| -2,326 \| \| 41 \| BC011455 \| A_24_P918891_riset1 \| 4,45E-02 \| -2,316 \| \| 42 \| GPR44 \| GPR44_riset2 \| 3,95E-02 \| -2,300 \| \| 43 \| ZNF174 \| A_24_P418878_riset1 \| 1,52E-02 \| -2,285 \| \| 44 \| SLC25A27 \| A_23_P81721_riset1 \| 2,53E-02 \| -2,231 \| \| 45 \| THC2391454 \| A_32_P62371_riset1 \| 4,05E-02 \| -2,204 \| \| 46 \| BHLHB2 \| A_24_P268676_riset1 \| 1,87E-03 \| -2,174 \| \| 47 \| AF136408 \| A_32_P509964_riset1 \| 1,05E-02 \| -2,150 \| \| 48 \| CRIP3 \| A_23_P156562_riset1 \| 3,05E-02 \| -2,085 \| \| 49 \| USP47 \| A_23_P113380_riset1 \| 3,48E-02 \| -2,065 \| \| 50 \| THAP5 \| A_24_P15586_riset1 \| 2,44E-02 \| -2,021 \| \| 51 \| MAPKAP1 \| A_23_P216894_riset1 \| 3,21E-02 \| -2,018 \| \| 52 \| TOX \| A_24_P226755_riset1 \| 4,09E-02 \| -2,002 \| \| 53 \| ZNF516 \| A_24_P39894_riset1 \| 4,91E-02 \| -2,002 \| \| 54 \| POLR3B \| A_23_P87732_riset1 \| 1,04E-02 \| -1,977 \| \| 55 \| ASB2 \| A_23_P205370_riset1 \| 1,95E-02 \| -1,964 \| \| 56 \| FOXK2 \| FOXK2_riset2 \| 1,67E-03 \| -1,956 \| \| 57 \| THC2378508 \| A_24_P813730_riset1 \| 1,96E-02 \| -1,943 \| \| 58 \| SUPT16H \| A_23_P151634_riset1 \| 3,82E-02 \| -1,936 \| \| 59 \| ABCA5 \| A_23_P78018_riset1 \| 1,31E-02 \| -1,929 \| \| 60 \| FAM54A \| A_23_P253752_riset1 \| 3,23E-02 \| -1,926 \| \| 61 \| C17orf100 \| A_32_P150391_riset1 \| 1,28E-03 \| -1,918 \| \| 62 \| BTLA \| MIL_PPPID399200354_riset1 \| 3,12E-02 \| -1,918 \| \| 63 \| PEX5 \| A_23_P48246_riset1 \| 2,07E-02 \| -1,917 \| \| 64 \| CSGALNACT1 \| A_23_P134835_riset1 \| 4,27E-02 \| -1,910 \| \| 65 \| LOC100128661 \| A_32_P32905_riset1 \| 1,56E-02 \| -1,906 \| \| 66 \| PCM1 \| A_24_P555510_riset1 \| 4,90E-03 \| -1,892 \| \| 67 \| DARS2 \| A_23_P148984_riset1 \| 3,20E-03 \| -1,876 \| \| 68 \| FKBP5 \| A_24_P38081_riset1 \| 1,96E-02 \| -1,870 \| \| 69 \| GALNT14 \| A_23_P67847_riset1 \| 1,46E-02 \| -1,864 \| \| 70 \| CPD \| A_24_P53282_riset1 \| 1,72E-02 \| -1,839 \| \| 71 \| PLK4 \| A_23_P155968_riset1 \| 2,35E-02 \| -1,826 \| \| 72 \| C16ORF55 \| A_24_P159335_riset1 \| 1,26E-02 \| -1,816 \| \| 73 \| TNFAIP2 \| A_23_P421423_riset1 \| 4,65E-02 \| -1,815 \| \| 74 \| ING1 \| A_23_P99437_riset1 \| 3,86E-02 \| -1,810 \| \| 75 \| SFRS18 \| A_23_P122615_riset1 \| 2,53E-02 \| -1,803 \| \| 76 \| SORL1 \| A_23_P87049_riset1 \| 2,52E-02 \| -1,802 \| \| 77 \| IL13RA1 \| IL13RA1_riset2 \| 6,96E-03 \| -1,797 \| \| 78 \| PPARA \| A_24_P570049_riset1 \| 3,46E-02 \| -1,794 \| \| 79 \| SESN1 \| A_24_P346339_riset1 \| 3,38E-02 \| -1,786 \| \| 80 \| ZNF41 \| A_23_P45234_riset1 \| 1,31E-02 \| -1,772 \| \| 81 \| GPX7 \| A_23_P73972_riset1 \| 4,26E-02 \| -1,766 \| \| 82 \| HS3ST3B1 \| A_24_P23625_riset1 \| 3,60E-02 \| -1,766 \| \| 83 \| FPRL1 \| A_23_P55649_riset1 \| 1,12E-02 \| -1,762 \| \| 84 \| CSF3R \| A_23_P126218_riset1 \| 2,00E-02 \| -1,758 \| \| 85 \| IL1RAP \| A_23_P170857_riset1 \| 2,47E-02 \| -1,754 \| \| 86 \| GFOD1 \| A_32_P15035_riset1 \| 3,51E-02 \| -1,753 \| \| 87 \| TP53INP1 \| TP53INP1_riset2 \| 3,00E-02 \| -1,751 \| \| 88 \| TGFBR2 \| A_23_P211957_riset1 \| 4,69E-02 \| -1,749 \| \| 89 \| NR4A2 \| A_23_P131208_riset1 \| 4,07E-02 \| -1,740 \| \| 90 \| SNX30 \| MIL_PPPID399200370_riset1 \| 2,01E-03 \| -1,737 \| \| 91 \| JAG1 \| A_23_P210763_riset1 \| 1,04E-02 \| -1,729 \| \| 92 \| LRRC28 \| A_23_P333150_riset1 \| 1,15E-02 \| -1,728 \| \| 93 \| IFNGR1 \| MIL_PPPID394307788_riset1 \| 3,31E-02 \| -1,719 \| \| 94 \| SHPRH \| A_23_P337790_riset1 \| 1,55E-02 \| -1,713 \| \| 95 \| HHEX \| A_23_P47034_riset1 \| 1,97E-02 \| -1,712 \| \| 96 \| CBX5 \| A_24_P620621_riset1 \| 2,71E-02 \| -1,712 \| \| 97 \| C10ORF128 \| A_23_P24097_riset1 \| 1,48E-02 \| -1,711 \| \| 98 \| CXCR4 \| A_23_P102000_riset1 \| 1,52E-02 \| -1,710 \| \| 99 \| TNPO1 \| A_24_P260440_riset1 \| 2,23E-02 \| -1,708 \| \| 100 \| LOC100170939 \| A_24_P710730_riset1 \| 1,04E-02 \| -1,693 \| \| 101 \| EDG1 \| A_23_P160117_riset1 \| 2,72E-02 \| -1,693 \| \| 102 \| F8 \| A_23_P217643_riset1 \| 4,17E-02 \| -1,679 \| \| 103 \| TMTC3 \| A_24_P141804_riset1 \| 4,78E-02 \| -1,674 \| \| 104 \| NR3C1 \| MIL_PPPID397416155_riset1 \| 4,39E-03 \| -1,665 \| \| 105 \| FYB \| A_24_P393740_riset1 \| 2,66E-02 \| -1,659 \| \| 106 \| CENPQ \| A_23_P70328_riset1 \| 4,64E-02 \| -1,657 \| \| 107 \| SSH2 \| MIL_PPPID394453393_riset1 \| 2,18E-02 \| -1,642 \| \| 108 \| ZNF24 \| A_23_P208041_riset1 \| 1,84E-03 \| -1,638 \| \| 109 \| SLC25A35 \| A_23_P366726_riset1 \| 4,81E-02 \| -1,636 \| \| 110 \| SERBP1 \| A_23_P337422_riset1 \| 1,29E-03 \| -1,635 \| \| 111 \| S100A9 \| A_23_P23044_riset1 \| 2,17E-02 \| -1,634 \| \| 112 \| MCL1 \| A_24_P336754_riset1 \| 1,79E-02 \| -1,630 \| \| 113 \| TSC1 \| A_24_P329635_riset1 \| 1,56E-03 \| -1,626 \| \| 114 \| ERLIN2 \| A_24_P372217_riset1 \| 3,42E-02 \| -1,625 \| \| 115 \| TF \| A_23_P212500_riset1 \| 4,91E-02 \| -1,625 \| \| 116 \| THC2441733 \| A_24_P734843_riset1 \| 2,04E-02 \| -1,622 \| \| 117 \| INSR \| MIL_PPPID397416339_riset1 \| 4,94E-02 \| -1,622 \| \| 118 \| BCKDHB \| A_24_P239664_riset1 \| 2,59E-02 \| -1,620 \| \| 119 \| THC2269190 \| A_32_P2883_riset1 \| 1,46E-02 \| -1,617 \| \| 120 \| ST3GAL6 \| A_23_P250800_riset1 \| 1,04E-02 \| -1,609 \| \| 121 \| NDUFC2 \| A_24_P364236_riset1 \| 4,62E-02 \| -1,605 \| \| 122 \| PPIF \| A_23_P202104_riset1 \| 1,01E-02 \| -1,589 \| \| 123 \| WDR73 \| A_23_P99917_riset1 \| 4,31E-03 \| -1,586 \| \| 124 \| RNF103 \| A_23_P56709_riset1 \| 3,23E-03 \| -1,583 \| \| 125 \| PDS5B \| A_23_P205098_riset1 \| 1,96E-02 \| -1,574 \| \| 126 \| MGEA5 \| MIL_PPPID399200218_riset1 \| 4,04E-02 \| -1,570 \| \| 127 \| UBR3 \| A_23_P5405_riset1 \| 5,85E-03 \| -1,565 \| \| 128 \| ZNF2 \| A_24_P28845_riset1 \| 3,36E-02 \| -1,562 \| \| 129 \| ZBTB16 \| A_23_P104804_riset1 \| 2,01E-02 \| -1,562 \| \| 130 \| FRG1B;LOC283788 \| A_24_P417526_riset1 \| 4,65E-02 \| -1,557 \| \| 131 \| THC2440839 \| A_32_P113317_riset1 \| 1,96E-02 \| -1,556 \| \| 132 \| PEX1 \| A_23_P20045_riset1 \| 1,00E-02 \| -1,555 \| \| 133 \| ZFP91 \| ZFP91_riset2 \| 1,26E-02 \| -1,553 \| \| 134 \| C3AR1 \| C3AR1_riset2 \| 4,33E-02 \| -1,551 \| \| 135 \| C14ORF106 \| A_23_P106162_riset1 \| 1,42E-02 \| -1,550 \| \| 136 \| SCYL3 \| A_23_P74320_riset1 \| 9,66E-03 \| -1,547 \| \| 137 \| SNX18 \| A_24_P180363_riset1 \| 2,90E-02 \| -1,546 \| \| 138 \| MRE11A \| A_24_P312671_riset1 \| 3,15E-02 \| -1,545 \| \| 139 \| SLC45A4 \| A_23_P384816_riset1 \| 1,64E-02 \| -1,533 \| \| 140 \| ZC3H11A \| A_32_P221076_riset1 \| 4,92E-02 \| -1,533 \| \| 141 \| NPEPPS \| A_23_P84836_riset1 \| 6,07E-03 \| -1,530 \| \| 142 \| CP110 \| A_24_P373286_riset1 \| 4,93E-02 \| -1,528 \| \| 143 \| PPWD1 \| A_23_P110433_riset1 \| 3,78E-02 \| -1,527 \| \| 144 \| TMEM157 \| A_23_P30283_riset1 \| 9,23E-03 \| -1,527 \| \| 145 \| TPR \| MIL_PPPID394453417_riset1 \| 2,59E-02 \| -1,522 \| \| 146 \| JARID1D \| MIL_PPPID394307968_riset1 \| 4,61E-02 \| -1,521 \| \| 147 \| CR610211 \| A_32_P181452_riset1 \| 4,70E-02 \| -1,518 \| \| 148 \| PELI2 \| A_23_P65532_riset1 \| 2,89E-03 \| -1,515 \| \| 149 \| SFT2D3 \| A_23_P5566_riset1 \| 1,92E-02 \| -1,509 \| \| 150 \| CEP110 \| A_23_P43580_riset1 \| 7,04E-03 \| -1,505 \| \| 151 \| SH3PXD2B \| A_32_P24585_riset1 \| 3,37E-02 \| -1,502 \| |
| --- | --- | --- | --- | --- | --- | --- | --- | --- | --- | --- | --- | --- | --- | --- | --- | --- | --- | --- | --- | --- | --- | --- | --- | --- | --- | --- | --- | --- | --- | --- | --- | --- | --- | --- | --- | --- | --- | --- | --- | --- | --- | --- | --- | --- | --- | --- | --- | --- | --- | --- | --- | --- | --- | --- | --- | --- | --- | --- | --- | --- | --- | --- | --- | --- | --- | --- | --- | --- | --- | --- | --- | --- | --- | --- | --- | --- | --- | --- | --- | --- | --- | --- | --- | --- | --- | --- | --- | --- | --- | --- | --- | --- | --- | --- | --- | --- | --- | --- | --- | --- | --- | --- | --- | --- | --- | --- | --- | --- | --- | --- | --- | --- | --- | --- | --- | --- | --- | --- | --- | --- | --- | --- | --- | --- | --- | --- | --- | --- | --- | --- | --- | --- | --- | --- | --- | --- | --- | --- | --- | --- | --- | --- | --- | --- | --- | --- | --- | --- | --- | --- | --- | --- | --- | --- | --- | --- | --- | --- | --- | --- | --- | --- | --- | --- | --- | --- | --- | --- | --- | --- | --- | --- | --- | --- | --- | --- | --- | --- | --- | --- | --- | --- | --- | --- | --- | --- | --- | --- | --- | --- | --- | --- | --- | --- | --- | --- | --- | --- | --- | --- | --- | --- | --- | --- | --- | --- | --- | --- | --- | --- | --- | --- | --- | --- | --- | --- | --- | --- | --- | --- | --- | --- | --- | --- | --- | --- | --- | --- | --- | --- | --- | --- | --- | --- | --- | --- | --- | --- | --- | --- | --- | --- | --- | --- | --- | --- | --- | --- | --- | --- | --- | --- | --- | --- | --- | --- | --- | --- | --- | --- | --- | --- | --- | --- | --- | --- | --- | --- | --- | --- | --- | --- | --- | --- | --- | --- | --- | --- | --- | --- | --- | --- | --- | --- | --- | --- | --- | --- | --- | --- | --- | --- | --- | --- | --- | --- | --- | --- | --- | --- | --- | --- | --- | --- | --- | --- | --- | --- | --- | --- | --- | --- | --- | --- | --- | --- | --- | --- | --- | --- | --- | --- | --- | --- | --- | --- | --- | --- | --- | --- | --- | --- | --- | --- | --- | --- | --- | --- | --- | --- | --- | --- | --- | --- | --- | --- | --- | --- | --- | --- | --- | --- | --- | --- | --- | --- | --- | --- | --- | --- | --- | --- | --- | --- | --- | --- | --- | --- | --- | --- | --- | --- | --- | --- | --- | --- | --- | --- | --- | --- | --- | --- | --- | --- | --- | --- | --- | --- | --- | --- | --- | --- | --- | --- | --- | --- | --- | --- | --- | --- | --- | --- | --- | --- | --- | --- | --- | --- | --- | --- | --- | --- | --- | --- | --- | --- | --- | --- | --- | --- | --- | --- | --- | --- | --- | --- | --- | --- | --- | --- | --- | --- | --- | --- | --- | --- | --- | --- | --- | --- | --- | --- | --- | --- | --- | --- | --- | --- | --- | --- | --- | --- | --- | --- | --- | --- | --- | --- | --- | --- | --- | --- | --- | --- | --- | --- | --- | --- | --- | --- | --- | --- | --- | --- | --- | --- | --- | --- | --- | --- | --- | --- | --- | --- | --- | --- | --- | --- | --- | --- | --- | --- | --- | --- | --- | --- | --- | --- | --- | --- | --- | --- | --- | --- | --- | --- | --- | --- | --- | --- | --- | --- | --- | --- | --- | --- | --- | --- | --- | --- | --- | --- | --- | --- | --- | --- | --- | --- | --- | --- | --- | --- | --- | --- | --- | --- | --- | --- | --- | --- | --- | --- | --- | --- | --- | --- | --- | --- | --- | --- | --- | --- | --- | --- | --- | --- | --- | --- | --- | --- | --- | --- | --- | --- | --- | --- | --- | --- | --- | --- | --- | --- | --- | --- | --- | --- | --- | --- | --- | --- | --- | --- | --- | --- | --- | --- | --- | --- | --- | --- | --- | --- | --- | --- | --- | --- | --- | --- | --- | --- | --- | --- | --- | --- | --- | --- | --- | --- | --- | --- | --- | --- | --- | --- | --- | --- | --- | --- | --- | --- | --- | --- | --- | --- | --- | --- | --- | --- | --- | --- | --- | --- | --- | --- | --- | --- | --- | --- | --- | --- | --- | --- | --- | --- | --- | --- | --- | --- | --- | --- | --- | --- | --- | --- | --- | --- | --- | --- | --- | --- | --- | --- | --- | --- | --- | --- | --- | --- | --- | --- | --- | --- | --- | --- | --- | --- | --- | --- | --- | --- | --- | --- | --- | --- | --- | --- | --- | --- | --- | --- | --- | --- | --- | --- | --- | --- | --- | --- | --- | --- | --- | --- | --- | --- | --- | --- | --- | --- | --- | --- | --- | --- | --- | --- | --- | --- | --- | --- | --- | --- | --- | --- | --- | --- | --- | --- | --- | --- | --- | --- | --- | --- | --- | --- | --- | --- | --- | --- | --- | --- | --- | --- | --- | --- | --- | --- | --- | --- | --- | --- | --- | --- | --- | --- | --- | --- | --- | --- | --- | --- |
