# Supplementary material for: Sequential Targeting of CD52 and TNF Allows Early Minimization Therapy in Kidney Transplantation: From a Biomarker to Targeting in a Proof-Of-Concept Trial
Source: PLoS One. 2017 Jan 13;12(1):e0169624. doi: 10.1371/journal.pone.0169624 (PMC5234822; doi:10.1371/journal.pone.0169624)
Supplement: S9 Table — Complete list of 134 probes ranked according to median fold change (only fold changes ≥1.5 were included) with corresponding p values (two-tailed t test) and microarray probe ID. (DOCX) [file pone.0169624.s013.docx]

| Supplemental Table S9. List of genes significantly up-regulated in M2-M12 samples of patients from tacrolimus group compared to patients from sirolimus group . Complete list of 134 probes ranked according to median fold change (only fold changes ≥1.5 were included) with corresponding p values (two-tailed t test) and microarray probe ID. | | | | |
| --- | --- | --- | --- | --- |
| **Rank** | **Gene Name** | **Probe ID** | **p** | **Fold change** |
| 1 | TCL1A | MIL_PPPID394307780_riset1 | 1,48E-03 | 13,85 |
| 2 | TCL1A | A_23_P357717_riset1 | 7,14E-04 | 12,52 |
| 3 | IGHM | A_24_P417352_riset1 | 1,28E-05 | 7,87 |
| 4 | CD79A | CD79A_riset2_piqor | 6,87E-04 | 6,65 |
| 5 | IGLC | MIL_PPPID399806199_riset1 | 8,35E-05 | 6,24 |
| 6 | CD200 | A_23_P121480_riset1 | 4,17E-04 | 6,20 |
| 7 | PCDH9 | A_24_P187218_riset1 | 1,74E-04 | 5,91 |
| 8 | IGHG1;IGHG2;IGHG3;IGHG4 | MIL_PPPID399806233_riset1 | 2,11E-04 | 5,81 |
| 9 | IGKC | MIL_PPPID399806182_riset1 | 9,52E-05 | 5,60 |
| 10 | BLK | BLK_riset2 | 2,33E-05 | 5,32 |
| 11 | THC2317432 | A_32_P124728_riset1 | 2,07E-05 | 5,20 |
| 12 | IGHA1 | A_23_P136026_riset1 | 3,78E-04 | 5,19 |
| 13 | ENST00000359488 | A_23_P21260_riset1 | 9,40E-05 | 5,19 |
| 14 | IGKV3-20 | A_23_P21800_riset1 | 5,38E-05 | 5,12 |
| 15 | MS4A1 | MIL_PPPID394453510_riset1 | 1,99E-03 | 4,95 |
| 16 | THC2438936 | A_32_P71876_riset1 | 4,44E-04 | 4,87 |
| 17 | ENST00000360623 | A_24_P24053_riset1 | 3,10E-04 | 4,79 |
| 18 | FCRL5 | A_23_P201211_riset1 | 9,13E-03 | 4,60 |
| 19 | LOC100132941 | A_24_P144346_riset1 | 1,66E-04 | 4,58 |
| 20 | CD22 | CD22_riset2 | 1,29E-04 | 4,56 |
| 21 | THC2385462 | A_32_P137819_riset1 | 4,88E-04 | 4,44 |
| 22 | HS3ST1 | A_23_P121657_riset1 | 9,66E-05 | 4,41 |
| 23 | ENST00000322032 | A_23_P61042_riset1 | 3,83E-03 | 4,24 |
| 24 | EBF1 | A_24_P156501_riset1 | 1,07E-05 | 4,01 |
| 25 | EBF1 | A_32_P197561_riset1 | 7,61E-05 | 3,94 |
| 26 | IGJ | A_23_P167168_riset1 | 1,51E-06 | 3,88 |
| 27 | LOC652494 | A_24_P100684_riset1 | 2,18E-04 | 3,87 |
| 28 | IGLL1 | A_24_P83102_riset1 | 3,00E-07 | 3,85 |
| 29 | ENST00000377226 | A_24_P494425_riset1 | 1,06E-04 | 3,81 |
| 30 | PLEKHG1 | A_24_P6517_riset1 | 3,01E-03 | 3,76 |
| 31 | FCRL2 | A_23_P160751_riset1 | 1,15E-04 | 3,53 |
| 32 | CD79B | A_23_P207201_riset1 | 1,48E-04 | 3,52 |
| 33 | IGLL3 | IGLL3_riset2 | 2,53E-07 | 3,48 |
| 34 | BTLA | MIL_PPPID399200354_riset1 | 9,09E-04 | 3,43 |
| 35 | CD19 | CD19_riset2 | 1,70E-04 | 3,40 |
| 36 | CCR7 | A_23_P343398_riset1 | 2,35E-02 | 3,30 |
| 37 | MGC29506 | A_23_P84596_riset1 | 1,59E-05 | 3,30 |
| 38 | AY062331 | A_24_P639701_riset1 | 6,51E-05 | 3,15 |
| 39 | FCER2 | A_23_P164773_riset1 | 7,19E-05 | 3,09 |
| 40 | IRF5 | A_24_P363609_riset1 | 4,89E-02 | 2,98 |
| 41 | BLNK | A_24_P64344_riset1 | 1,15E-04 | 2,92 |
| 42 | FLJ36131;LOC283767;LOC440233;LOC440243;LOC440320;LOC645202;LOC727832;LOC729272;LOC730027 | A_24_P50972_riset1 | 5,21E-04 | 2,90 |
| 43 | DTX1 | A_24_P290751_riset1 | 5,76E-04 | 2,82 |
| 44 | AFF3 | A_23_P373464_riset1 | 3,60E-04 | 2,81 |
| 45 | PI3 | A_23_P210465_riset1 | 2,36E-02 | 2,76 |
| 46 | CCR6 | A_24_P234921_riset1 | 4,09E-04 | 2,73 |
| 47 | FCGBP | A_23_P21495_riset1 | 9,23E-03 | 2,56 |
| 48 | MGC24039 | A_23_P366559_riset1 | 3,45E-04 | 2,52 |
| 49 | PNOC | A_23_P253321_riset1 | 4,67E-09 | 2,45 |
| 50 | MGC24039 | A_23_P379746_riset1 | 8,30E-06 | 2,41 |
| 51 | RAB30 | A_23_P139359_riset1 | 4,06E-08 | 2,40 |
| 52 | CLEC4C | CLEC4C_riset2 | 2,61E-03 | 2,39 |
| 53 | IGLV3-19 | A_24_P161764_riset1 | 1,91E-03 | 2,32 |
| 54 | HLA-DQA2 | A_24_P852756_riset1 | 3,82E-02 | 2,30 |
| 55 | AF471475 | A_23_P259763_riset1 | 2,43E-03 | 2,27 |
| 56 | P2RX5 | A_23_P413760_riset1 | 2,25E-05 | 2,25 |
| 57 | IL28RA | IL28RA_riset2 | 3,13E-03 | 2,21 |
| 58 | GNG7 | A_32_P136295_riset1 | 5,81E-06 | 2,16 |
| 59 | FCRLA | A_23_P46037_riset1 | 1,57E-06 | 2,13 |
| 60 | IGHD | A_24_P135384_riset1 | 1,51E-04 | 2,11 |
| 61 | COL6A2 | MIL_PPPID394307828_riset1 | 2,05E-02 | 2,05 |
| 62 | HBG1;HBG2 | A_23_P64539_riset1 | 1,13E-02 | 2,05 |
| 63 | GARNL4 | A_23_P412214_riset1 | 4,13E-02 | 2,05 |
| 64 | PVALB | A_23_P17844_riset1 | 5,00E-02 | 2,05 |
| 65 | SLC30A4 | A_32_P21255_riset1 | 6,80E-03 | 2,02 |
| 66 | BCL7A | A_24_P203056_riset1 | 1,80E-02 | 2,01 |
| 67 | CCL5 | A_23_P152837_riset1 | 1,38E-04 | 1,99 |
| 68 | CFH;CFHR1 | A_23_P114740_riset1 | 4,32E-03 | 1,98 |
| 69 | HLA-DOA | A_32_P356316_riset1 | 3,67E-03 | 1,92 |
| 70 | PTPRCAP | A_23_P98173_riset1 | 1,49E-04 | 1,91 |
| 71 | GM2A | A_23_P144866_riset1 | 9,88E-04 | 1,90 |
| 72 | CHI3L1 | A_23_P137665_riset1 | 3,46E-02 | 1,89 |
| 73 | AA789123 | A_32_P485325_riset1 | 2,63E-02 | 1,89 |
| 74 | CDKN2A | A_23_P43484_riset1 | 1,32E-03 | 1,88 |
| 75 | JUP | A_23_P501822_riset1 | 4,13E-05 | 1,86 |
| 76 | GPX3 | A_23_P133474_riset1 | 9,51E-04 | 1,86 |
| 77 | SCML4 | A_23_P8297_riset1 | 2,19E-03 | 1,86 |
| 78 | SLC47A1 | A_24_P142503_riset1 | 1,05E-04 | 1,85 |
| 79 | PAM | A_24_P97703_riset1 | 4,96E-03 | 1,84 |
| 80 | KLK1 | A_23_P16252_riset1 | 4,33E-03 | 1,84 |
| 81 | TLR3 | A_23_P29922_riset1 | 1,14E-02 | 1,83 |
| 82 | A_24_P918677 | A_24_P918677_riset1 | 6,80E-04 | 1,82 |
| 83 | TLR10 | MIL_PPPID399200190_riset1 | 3,02E-04 | 1,81 |
| 84 | PROC | A_23_P40096_riset1 | 7,74E-03 | 1,80 |
| 85 | PLGLA | A_23_P28607_riset1 | 3,85E-02 | 1,80 |
| 86 | IL6 | A_23_P71037_riset1 | 1,11E-04 | 1,78 |
| 87 | NELL2 | A_23_P10025_riset1 | 4,37E-02 | 1,78 |
| 88 | MYO1B | A_23_P361049_riset1 | 2,77E-06 | 1,78 |
| 89 | CRYM | A_23_P77731_riset1 | 6,33E-04 | 1,77 |
| 90 | A_24_P110487 | A_24_P110487_riset1 | 9,24E-05 | 1,76 |
| 91 | FADS3 | A_23_P64399_riset1 | 2,46E-07 | 1,74 |
| 92 | NEB | A_23_P146783_riset1 | 3,59E-03 | 1,73 |
| 93 | ST5 | A_23_P24884_riset1 | 2,76E-04 | 1,73 |
| 94 | RASA4 | A_24_P943263_riset1 | 1,40E-02 | 1,72 |
| 95 | THC2409569 | A_32_P93352_riset1 | 1,03E-06 | 1,72 |
| 96 | ABLIM1 | A_23_P202520_riset1 | 4,37E-04 | 1,71 |
| 97 | L3MBTL | A_23_P210445_riset1 | 1,22E-02 | 1,71 |
| 98 | LY9 | MIL_PPPID397416255_riset1 | 1,31E-02 | 1,69 |
| 99 | HLA-DOB | A_23_P30736_riset1 | 1,63E-02 | 1,68 |
| 100 | TRA@ | MIL_PPPID399200166_riset1 | 1,32E-02 | 1,68 |
| 101 | BLM | A_23_P88630_riset1 | 9,16E-04 | 1,68 |
| 102 | FA2H | A_23_P49448_riset1 | 1,80E-03 | 1,67 |
| 103 | POMC | A_23_P5875_riset1 | 1,71E-02 | 1,67 |
| 104 | CR613436 | A_32_P27135_riset1 | 2,11E-05 | 1,66 |
| 105 | STARD9 | A_32_P130641_riset1 | 2,81E-02 | 1,66 |
| 106 | STAG3 | A_23_P145657_riset1 | 2,06E-04 | 1,64 |
| 107 | IKZF3 | A_23_P376060_riset1 | 1,19E-02 | 1,62 |
| 108 | TRAF5 | TRAF5_riset2 | 2,06E-02 | 1,62 |
| 109 | LOC645638 | A_24_P691826_riset1 | 5,87E-03 | 1,62 |
| 110 | SCML4 | A_24_P930756_riset1 | 6,90E-03 | 1,61 |
| 111 | IL9R | IL9R_riset2 | 1,13E-02 | 1,61 |
| 112 | CD81 | A_23_P13423_riset1 | 2,63E-04 | 1,60 |
| 113 | THC2378994 | A_32_P117666_riset1 | 1,93E-03 | 1,60 |
| 114 | IGFBP4 | A_23_P38574_riset1 | 4,59E-02 | 1,60 |
| 115 | BI759100 | A_32_P139391_riset1 | 5,47E-04 | 1,59 |
| 116 | LOC23117 | A_32_P142664_riset1 | 9,13E-10 | 1,59 |
| 117 | CD5 | A_24_P364221_riset1 | 3,60E-02 | 1,58 |
| 118 | THC2349739 | A_32_P26422_riset1 | 4,52E-03 | 1,57 |
| 119 | CELSR1 | A_32_P157471_rev_riset1 | 1,07E-03 | 1,57 |
| 120 | PLEKHA4 | A_24_P408047_riset1 | 3,63E-04 | 1,56 |
| 121 | CD180 | A_23_P257815_riset1 | 1,81E-02 | 1,55 |
| 122 | GPNMB | A_23_P134426_riset1 | 3,06E-03 | 1,55 |
| 123 | ZHX2 | A_23_P168951_riset1 | 2,49E-05 | 1,55 |
| 124 | C11ORF24 | A_23_P12911_riset1 | 2,90E-04 | 1,55 |
| 125 | PRICKLE1 | A_23_P408285_riset1 | 2,61E-02 | 1,54 |
| 126 | IL4R | IL4R_1_riset2 | 4,82E-02 | 1,54 |
| 127 | KLHDC1 | A_23_P422766_riset1 | 4,93E-02 | 1,54 |
| 128 | BCKDHB | A_24_P239664_riset1 | 4,14E-02 | 1,53 |
| 129 | AKAP2 | AKAP2_riset2 | 1,96E-03 | 1,53 |
| 130 | SLC41A1 | A_24_P122732_riset1 | 1,37E-02 | 1,53 |
| 131 | STMN1 | A_23_P200866_riset1 | 1,05E-02 | 1,52 |
| 132 | DDIT4 | A_23_P104318_riset1 | 1,52E-03 | 1,52 |
| 133 | GPR92 | A_23_P204375_riset1 | 2,43E-03 | 1,52 |
| 134 | AUTS2 | A_23_P122906_riset1 | 1,01E-02 | 1,51 |
