# Supplementary material for: Sequential Targeting of CD52 and TNF Allows Early Minimization Therapy in Kidney Transplantation: From a Biomarker to Targeting in a Proof-Of-Concept Trial
Source: PLoS One. 2017 Jan 13;12(1):e0169624. doi: 10.1371/journal.pone.0169624 (PMC5234822; doi:10.1371/journal.pone.0169624)
Supplement: S10 Table — Complete list of 136 probes ranked according to median fold change (only fold changes ≥1.5 were included) with corresponding p values (two-tailed t test) and microarray probe ID. (DOCX) [file pone.0169624.s014.docx]

| Supplemental Table S10. List of genes significantly down-regulated in M2-M12 samples of patients from tacrolimus group compared to patients from Sirolimus group . Complete list of 136 probes ranked according to median fold change (only fold changes ≥1.5 were included) with corresponding p values (two-tailed t test) and microarray probe ID. | | | | |
| --- | --- | --- | --- | --- |
| **Rank** | **Gene Name** | **Probe ID** | **p** | **Fold change** |
| 1 | FN1 | MIL_PPPID394453454_riset1 | 2,37E-07 | -4,53 |
| 2 | IL8 | A_32_P87013_riset1 | 3,58E-02 | -3,67 |
| 3 | PLEK2 | A_23_P151506_riset1 | 6,29E-05 | -3,53 |
| 4 | C4A;C4B | MIL_PPPID394453482_riset1 | 7,47E-04 | -3,32 |
| 5 | LOC375010;LOC401131;LOC643166;LOC643579;LOC728295;LOC728364;LOC728384;LOC728759;LOC728783 | A_32_P79190_riset1 | 1,09E-07 | -3,23 |
| 6 | TGM2 | A_32_P86763_riset1 | 1,44E-06 | -3,19 |
| 7 | TNNT1 | MIL_PPPID394307796_riset1 | 2,76E-07 | -3,00 |
| 8 | MMP8 | A_23_P24493_riset1 | 5,17E-03 | -2,99 |
| 9 | FN1 | A_23_P209925_riset1 | 1,86E-05 | -2,97 |
| 10 | INHBA | A_23_P122924_riset1 | 1,38E-04 | -2,95 |
| 11 | FECH | MIL_PPPID394453409_riset1 | 1,25E-02 | -2,85 |
| 12 | CTNNAL1 | A_23_P157795_riset1 | 8,67E-04 | -2,70 |
| 13 | MAGEA10 | A_32_P17253_riset1 | 2,21E-04 | -2,58 |
| 14 | AF263545 | A_24_P926507_riset1 | 9,38E-03 | -2,52 |
| 15 | BNIP3L | BNIP3L_riset2 | 3,41E-03 | -2,50 |
| 16 | FLJ25371 | A_23_P386964_riset1 | 2,23E-02 | -2,41 |
| 17 | FN1 | A_24_P119745_riset1 | 1,47E-03 | -2,37 |
| 18 | VMO1 | A_23_P55356_riset1 | 8,76E-03 | -2,35 |
| 19 | FZD5 | A_24_P148503_riset1 | 2,27E-03 | -2,33 |
| 20 | SPTA1 | A_23_P63158_riset1 | 2,78E-03 | -2,33 |
| 21 | AQP1 | A_23_P19894_riset1 | 1,52E-05 | -2,29 |
| 22 | KANK2 | A_23_P50426_riset1 | 4,69E-03 | -2,25 |
| 23 | SAA1 | MIL_PPPID399806220_riset1 | 5,40E-04 | -2,22 |
| 24 | OLR1 | A_24_P124624_riset1 | 6,31E-03 | -2,21 |
| 25 | KLF1 | A_32_P135555_riset1 | 1,02E-02 | -2,19 |
| 26 | CYORF14 | A_24_P216625_riset1 | 1,24E-02 | -2,18 |
| 27 | HBB;HBD | A_23_P87346_riset1 | 1,37E-02 | -2,18 |
| 28 | ARHGEF12 | A_24_P175783_riset1 | 1,25E-02 | -2,16 |
| 29 | NEDD4L | A_23_P387856_riset1 | 8,48E-03 | -2,09 |
| 30 | GYPA | GYPA_riset2 | 1,63E-02 | -2,09 |
| 31 | ERG | A_23_P57323_riset1 | 4,91E-03 | -2,07 |
| 32 | ABCC4 | A_24_P16913_riset1 | 4,65E-03 | -2,04 |
| 33 | CNRIP1 | A_23_P329353_riset1 | 5,95E-04 | -2,00 |
| 34 | SPP1 | A_23_P7313_riset1 | 1,54E-03 | -1,99 |
| 35 | CHIT1 | A_23_P126278_riset1 | 3,00E-04 | -1,99 |
| 36 | GH1 | MIL_PPPID399806221_riset1 | 4,38E-02 | -1,95 |
| 37 | RFESD | A_23_P251647_riset1 | 1,38E-02 | -1,95 |
| 38 | SHOX | A_23_P22761_riset1 | 3,23E-02 | -1,95 |
| 39 | TMPRSS9 | A_23_P209176_riset1 | 1,30E-02 | -1,94 |
| 40 | LCN2 | A_23_P169437_riset1 | 2,07E-03 | -1,94 |
| 41 | SFTPC | A_23_P95213_riset1 | 1,36E-03 | -1,94 |
| 42 | THC2317292 | A_32_P5148_riset1 | 1,32E-03 | -1,93 |
| 43 | IFNA13 | IFNA13_riset2 | 3,99E-02 | -1,93 |
| 44 | AKR1C1 | MIL_PPPID399806218_riset1 | 5,39E-03 | -1,93 |
| 45 | FOXO3 | A_32_P493575_riset1 | 1,10E-03 | -1,91 |
| 46 | IL5RA | A_23_P500676_riset1 | 9,35E-04 | -1,91 |
| 47 | AKR1C1;AKR1C2 | A_24_P220947_riset1 | 2,04E-02 | -1,90 |
| 48 | HBM | A_23_P15055_riset1 | 3,56E-03 | -1,89 |
| 49 | BC042064 | A_32_P112677_riset1 | 1,54E-02 | -1,89 |
| 50 | MYO3A | A_23_P86411_riset1 | 1,36E-02 | -1,89 |
| 51 | HPR | A_23_P421493_riset1 | 1,77E-02 | -1,88 |
| 52 | PIP4K2A | A_23_P104344_riset1 | 3,73E-03 | -1,87 |
| 53 | NUSAP1 | A_24_P416079_riset2 | 7,19E-03 | -1,86 |
| 54 | TSPAN5 | A_23_P323930_riset1 | 1,26E-03 | -1,84 |
| 55 | FUT1 | A_23_P107963_riset1 | 2,43E-03 | -1,83 |
| 56 | EMR4 | MIL_PPPID399200170_riset1 | 2,59E-02 | -1,83 |
| 57 | CKAP2L | A_32_P208011_riset1 | 3,97E-02 | -1,82 |
| 58 | SNHG10 | A_32_P82475_riset1 | 2,20E-04 | -1,80 |
| 59 | GPT | A_23_P146339_riset1 | 1,57E-05 | -1,79 |
| 60 | NFIA | A_32_P131998_riset1 | 6,54E-03 | -1,77 |
| 61 | DARC | A_23_P115154_riset1 | 2,53E-02 | -1,77 |
| 62 | LOC643194 | A_24_P795230_riset1 | 1,37E-04 | -1,77 |
| 63 | MKRN1 | A_23_P59798_riset1 | 3,44E-03 | -1,77 |
| 64 | HP | MIL_PPPID399806176_riset1 | 1,97E-02 | -1,76 |
| 65 | ENTPD5 | A_23_P117580_riset1 | 1,92E-02 | -1,76 |
| 66 | TNFAIP6 | A_23_P165624_riset1 | 3,83E-03 | -1,76 |
| 67 | LOC643008 | A_32_P445010_riset1 | 1,82E-02 | -1,75 |
| 68 | FREQ | A_23_P217049_riset1 | 2,95E-02 | -1,74 |
| 69 | CXCL9 | A_23_P18452_riset1 | 2,70E-02 | -1,74 |
| 70 | C1ORF88 | A_23_P424900_riset1 | 1,46E-03 | -1,72 |
| 71 | MXI1 | MIL_PPPID394307804_riset1 | 5,64E-03 | -1,71 |
| 72 | CDC42BPA | A_23_P256190_riset1 | 1,70E-03 | -1,71 |
| 73 | GMPR | A_23_P257462_riset1 | 3,60E-02 | -1,70 |
| 74 | MAP3K7IP3 | A_23_P305033_riset1 | 7,15E-05 | -1,70 |
| 75 | FCGR1A | A_23_P63395_riset1 | 1,51E-03 | -1,69 |
| 76 | BC042064 | A_24_P838797_riset1 | 3,30E-02 | -1,69 |
| 77 | TNS1 | A_24_P105733_riset1 | 9,34E-04 | -1,69 |
| 78 | OLIG2 | A_23_P211079_riset1 | 1,12E-02 | -1,69 |
| 79 | LOXL1 | A_23_P124084_riset1 | 7,66E-04 | -1,68 |
| 80 | CDH1 | A_23_P206359_riset1 | 2,86E-03 | -1,67 |
| 81 | PBX1 | A_23_P62948_riset1 | 1,83E-02 | -1,66 |
| 82 | IL1RL1 | A_23_P51126_riset1 | 3,27E-03 | -1,66 |
| 83 | MAP4K5 | A_23_P205646_riset1 | 7,50E-04 | -1,66 |
| 84 | LRP2 | MIL_PPPID397416171_riset1 | 1,46E-02 | -1,66 |
| 85 | MPP1 | A_23_P171296_riset1 | 4,06E-03 | -1,66 |
| 86 | WNK1 | A_24_P769359_riset1 | 5,40E-03 | -1,65 |
| 87 | IL3RA | A_32_P217750_riset1 | 1,24E-02 | -1,65 |
| 88 | THC2377845 | A_32_P99804_riset1 | 6,60E-04 | -1,64 |
| 89 | CHST11 | A_23_P139919_riset1 | 5,07E-05 | -1,63 |
| 90 | CCDC18 | A_23_P51805_riset1 | 2,70E-03 | -1,63 |
| 91 | TRAK2 | A_23_P209426_riset1 | 2,60E-03 | -1,63 |
| 92 | PCAF | A_32_P159651_riset1 | 7,87E-03 | -1,62 |
| 93 | PPP3R1;WDR92 | A_24_P388252_riset1 | 3,01E-03 | -1,62 |
| 94 | AF087985 | A_32_P230595_riset1 | 2,74E-02 | -1,61 |
| 95 | SRRD;TFIP11 | A_32_P135382_riset1 | 3,73E-02 | -1,61 |
| 96 | PTGER3 | MIL_PPPID397416191_riset1 | 4,27E-03 | -1,61 |
| 97 | CDKN3 | A_23_P48669_riset1 | 3,38E-03 | -1,61 |
| 98 | COX7B | A_23_P159650_riset1 | 1,60E-02 | -1,61 |
| 99 | LAMA3 | LAMA3_riset2 | 4,05E-02 | -1,60 |
| 100 | SLC14A1 | A_23_P38757_riset1 | 3,11E-02 | -1,60 |
| 101 | TRAF4 | TRAF4_riset2 | 3,93E-02 | -1,60 |
| 102 | CCR2;FLJ78302 | A_23_P324885_riset1 | 4,81E-02 | -1,60 |
| 103 | DEPDC1 | A_23_P200310_riset1 | 7,56E-04 | -1,59 |
| 104 | CAMP | A_23_P253791_riset1 | 4,75E-02 | -1,57 |
| 105 | C10ORF10 | A_24_P329795_riset1 | 4,90E-03 | -1,57 |
| 106 | CREBL2 | A_23_P14026_riset1 | 6,00E-03 | -1,56 |
| 107 | PGM2L1 | A_32_P122703_riset1 | 9,20E-03 | -1,56 |
| 108 | EIF2C2 | A_23_P112159_riset1 | 5,61E-03 | -1,56 |
| 109 | THC2280867 | A_32_P122715_riset1 | 1,61E-02 | -1,56 |
| 110 | IL15 | MIL_PPPID394307956_riset1 | 1,53E-03 | -1,55 |
| 111 | LYNX1 | MIL_PPPID394453345_riset1 | 4,38E-02 | -1,55 |
| 112 | PGC | A_23_P7961_riset1 | 1,84E-03 | -1,55 |
| 113 | AMBP | A_23_P363754_riset1 | 3,60E-03 | -1,54 |
| 114 | P2RY14 | A_24_P165864_riset1 | 3,78E-02 | -1,54 |
| 115 | BCAT1 | A_24_P935986_riset1 | 2,45E-03 | -1,54 |
| 116 | PIP5K1B | A_32_P465742_riset1 | 1,25E-02 | -1,54 |
| 117 | FAHD1 | MIL_PPPID394453514_riset1 | 6,40E-03 | -1,54 |
| 118 | POLE2 | A_23_P163099_riset1 | 5,45E-03 | -1,54 |
| 119 | MAP2K3 | MAP2K3_riset2 | 1,35E-02 | -1,54 |
| 120 | STC1 | A_23_P314755_riset1 | 2,42E-02 | -1,54 |
| 121 | POU2F1 | A_23_P46070_riset1 | 8,20E-04 | -1,53 |
| 122 | BSG | A_24_P134266_riset1 | 3,06E-02 | -1,53 |
| 123 | TUSC1 | A_23_P373819_riset1 | 1,08E-02 | -1,53 |
| 124 | GPR84 | A_23_P25155_riset1 | 1,07E-02 | -1,53 |
| 125 | PIP4K2A | A_23_P104346_riset1 | 5,54E-03 | -1,53 |
| 126 | DOCK4 | A_23_P364580_riset1 | 4,71E-02 | -1,53 |
| 127 | PTN | A_23_P134213_riset1 | 7,11E-03 | -1,52 |
| 128 | JAG1 | A_23_P210763_riset1 | 2,31E-02 | -1,52 |
| 129 | LMNA | A_24_P162718_riset1 | 2,41E-02 | -1,52 |
| 130 | LTF | MIL_PPPID397416163_riset1 | 1,77E-02 | -1,52 |
| 131 | SLC2A4 | A_32_P151263_riset1 | 3,92E-03 | -1,51 |
| 132 | SLC22A2 | A_23_P111395_riset1 | 3,41E-02 | -1,51 |
| 133 | ORM2 | A_23_P9485_riset1 | 3,79E-02 | -1,51 |
| 134 | CCNI | A_23_P69521_riset1 | 2,57E-03 | -1,50 |
| 135 | C1ORF26 | A_23_P96931_riset1 | 3,71E-03 | -1,50 |
| 136 | EMP1 | A_23_P76488_riset1 | 2,89E-02 | -1,50 |
