# Supplementary material for: Sequential Targeting of CD52 and TNF Allows Early Minimization Therapy in Kidney Transplantation: From a Biomarker to Targeting in a Proof-Of-Concept Trial
Source: PLoS One. 2017 Jan 13;12(1):e0169624. doi: 10.1371/journal.pone.0169624 (PMC5234822; doi:10.1371/journal.pone.0169624)
Supplement: S11 Table — Only the first 15 annotations with the highest statistical significance are shown. (DOCX) [file pone.0169624.s015.docx]

Supplemental Table 11 Annotation enrichment analysis of genes with significantly higher differential expression in M2-M12 samples of patients treated with Tacrolimus relative to the Sirolimus group. Only the first 15 annotations with the highest statistical significance are shown.

| **Annotation** | **transcriptional cell markers: B cells (Chtanova et al. J. Immunol. 2005)** | **Immunoglobulin domain containing** | **part of antibody** | **genes upregulated in monocytes at late time points after IL-10 treatment (Jung et al., Eur. J. Immunol. 2004)** | **B cell receptor signaling** | **B cell surface marker** | **Downregulated by Notch Pathway** | **Hematopoietic cell lineage** | **Immunity** | **Dendritic cell surface marker** | **Upregulated by IL7 Pathway** | **ctcf: first multivalent nuclear factor** | **Signaling by GPCR** | **IL-4 Pathway** | **Chemokine receptors bind chemokines** |
| --- | --- | --- | --- | --- | --- | --- | --- | --- | --- | --- | --- | --- | --- | --- | --- |
| **Significance** | 1.61E-21 | 3.65E-07 | 3.65E-07 | 7.90E-05 | 1.42E-04 | 1.06E-03 | 1.11E-03 | 1.70E-03 | 2.62E-03 | 7.59E-03 | 1.15E-02 | 2.56E-02 | 3.90E-02 | 4.42E-02 | 4.94E-02 |
| **Enrichment** | 25.4 | 31.8 | 31.8 | 5.21 | 9.77 | 11.9 | 9.34 | 5.6 | 3.26 | 8.66 | 4.43 | 6.35 | 4.67 | 4.88 | 5.29 |
| **Count** | 16 | 22 | 10 | 11 | 11 | 10 | 5 | 6 | 12 | 5 | 6 | 3 | 6 | 6 | 3 |
| **Name** |  |  |  |  |  |  |  |  |  |  |  |  |  |  |  |
| BLK | **X** |  |  |  | **X** |  |  |  |  |  |  |  |  |  |  |
| BLNK | **X** |  |  |  | **X** |  |  |  |  |  |  |  |  |  |  |
| BTLA |  | **X** |  |  |  |  |  |  | **X** |  |  |  |  |  |  |
| CCL5 |  |  |  |  |  |  |  |  |  |  | **X** |  | **X** |  | **X** |
| CCR6 |  |  |  |  |  |  |  |  |  | **X** |  |  | **X** |  | **X** |
| CCR7 |  |  |  |  |  |  |  |  |  | **X** |  |  | **X** |  | **X** |
| CD180 |  |  |  |  |  | **X** |  |  | **X** | **X** |  |  |  |  |  |
| CD19 | **X** | **X** |  |  | **X** | **X** |  | **X** |  |  | **X** |  |  |  |  |
| CD200 |  | **X** |  |  |  |  |  |  |  |  |  |  |  |  |  |
| CD22 |  | **X** |  |  | **X** | **X** |  | **X** |  |  |  |  |  |  |  |
| CD5 |  |  |  |  | **X** | **X** |  | **X** |  |  |  |  |  |  |  |
| CD79A |  | **X** |  |  | **X** | **X** |  |  | **X** |  |  | **X** |  |  |  |
| CD79B | **X** | **X** |  |  | **X** | **X** | **X** |  | **X** |  |  | **X** |  |  |  |
| CD81 |  |  |  |  | **X** | **X** |  |  |  | **X** |  |  |  |  |  |
| CDKN2A |  |  |  |  |  |  |  |  |  |  |  | **X** |  |  |  |
| CFH;CFHR1 |  |  |  |  |  |  |  |  | **X** |  |  |  |  |  |  |
| CLEC4C |  |  |  |  |  |  |  |  | **X** |  |  |  |  |  |  |
| EBF1 |  |  |  |  |  |  | **X** |  |  |  | **X** |  |  |  |  |
| FCER2 |  |  |  |  |  | **X** |  | **X** |  |  | **X** |  |  | **X** |  |
| FCGBP |  |  |  | **X** |  |  |  |  |  |  |  |  |  |  |  |
| FCRL2 | **X** | **X** |  |  |  |  |  |  |  |  |  |  |  |  |  |
| FCRL5 |  | **X** |  |  |  | **X** |  |  |  |  |  |  |  |  |  |
| FCRLA |  | **X** |  |  |  |  |  |  |  |  |  |  |  |  |  |
| GNG7 |  |  |  |  |  |  |  |  |  |  |  |  | **X** |  |  |
| HBG1;HBG2 |  |  |  |  |  |  |  |  |  |  |  |  |  | **X** |  |
| HLA-DOA |  | **X** |  |  |  |  |  |  | **X** |  |  |  |  | **X** |  |
| HLA-DOB | **X** | **X** |  |  |  |  |  |  | **X** |  |  |  |  | **X** |  |
| HLA-DQA2 |  | **X** |  |  |  |  |  |  | **X** |  |  |  |  | **X** |  |
| HS3ST1 |  |  |  | **X** |  |  |  |  |  |  |  |  |  |  |  |
| IGFBP4 |  |  |  | **X** |  |  |  |  |  |  |  |  |  |  |  |
| IGHA1 | **X** | **X** | **X** | **X** |  |  |  |  |  |  |  |  |  |  |  |
| IGHD | **X** | **X** | **X** |  |  |  |  |  |  |  |  |  |  |  |  |
| IGHG1;IGHG2; IGHG3;IGHG4 | **X** | **X** | **X** | **X** |  |  |  |  |  |  |  |  |  |  |  |
| IGJ | **X** | **X** | **X** |  |  |  | **X** |  |  |  |  |  |  |  |  |
| IGKC | **X** | **X** | **X** |  |  |  |  |  |  |  |  |  |  |  |  |
| IGKV3-20 |  | **X** | **X** |  |  |  |  |  |  |  |  |  |  |  |  |
| IGLC |  |  | **X** | **X** |  |  |  |  |  |  |  |  |  |  |  |
| IGLL1 |  | **X** | **X** |  |  |  |  |  |  |  | **X** |  |  |  |  |
| IGLL3 | **X** | **X** | **X** |  |  |  |  |  |  |  |  |  |  |  |  |
| IL4R |  |  |  |  | **X** |  |  |  | **X** |  |  |  |  | **X** |  |
| IL6 |  |  |  | **X** |  |  |  |  |  |  |  |  |  |  |  |
| IL9R |  |  |  |  |  |  |  | **X** |  |  | **X** |  |  |  |  |
| JUP |  |  |  |  |  |  | **X** |  |  |  |  |  |  |  |  |
| LOC652494 | **X** | **X** | **X** | **X** | **X** |  | **X** |  |  |  |  |  |  |  |  |
| LY9 |  | **X** |  |  |  |  |  |  |  |  |  |  |  |  |  |
| MS4A1 | **X** |  |  | **X** |  | **X** |  | **X** |  |  |  |  |  |  |  |
| PNOC | **X** |  |  |  |  |  |  |  |  |  |  |  | **X** |  |  |
| POMC |  |  |  |  |  |  |  |  |  |  |  |  | **X** |  |  |
| TCL1A | **X** |  |  |  | **X** |  |  |  |  |  |  |  |  |  |  |
| TLR10 |  |  |  |  |  |  |  |  | **X** |  |  |  |  |  |  |
| TLR3 |  |  |  |  |  |  |  |  | **X** | **X** |  |  |  |  |  |
| TRAF5 |  |  |  | **X** |  |  |  |  |  |  |  |  |  |  |  |
| ZHX2 |  |  |  | **X** |  |  |  |  |  |  |  |  |  |  |  |
